# Basal tolerance but not plasticity gives invasive springtails the advantage in an assemblage setting

**DOI:** 10.1093/conphys/coaa049

**Published:** 2020-06-15

**Authors:** Laura M Phillips, Ian Aitkenhead, Charlene Janion-Scheepers, Catherine K King, Melodie A McGeoch, Uffe N Nielsen, Aleks Terauds, W P Amy Liu, Steven L Chown

**Affiliations:** 1 School of Biological Sciences, Monash University, Victoria 3800, Australia; 2 Iziko South African Museum, Cape Town 8001, South Africa; 3 Department of Biological Sciences, University of Cape Town, Rondebosch, Cape Town 7700, South Africa; 4 Australian Antarctic Division, Department of Agriculture, Water and the Environment, 203 Channel Highway, Kingston, Tasmania 7050, Australia; 5 Hawkesbury Institute for the Environment, Western Sydney University, Locked Bag 1797, Penrith, New South Wales, 2751, Australia

**Keywords:** Biological invasions, climate change, *CT_max_*, *CT_min_*, growth, water balance

## Abstract

As global climates change, alien species are anticipated to have a growing advantage relative to their indigenous counterparts, mediated through consistent trait differences between the groups. These insights have largely been developed based on interspecific comparisons using multiple species examined from different locations. Whether such consistent physiological trait differences are present within assemblages is not well understood, especially for animals. Yet, it is at the assemblage level that interactions play out. Here, we examine whether physiological trait differences observed at the interspecific level are also applicable to assemblages. We focus on the Collembola, an important component of the soil fauna characterized by invasions globally, and five traits related to fitness: critical thermal maximum, minimum and range, desiccation resistance and egg development rate. We test the predictions that the alien component of a local assemblage has greater basal physiological tolerances or higher rates, and more pronounced phenotypic plasticity than the indigenous component. Basal critical thermal maximum, thermal tolerance range, desiccation resistance, optimum temperature for egg development, the rate of development at that optimum and the upper temperature limiting egg hatching success are all significantly higher, on average, for the alien than the indigenous components of the assemblage. Outcomes for critical thermal minimum are variable. No significant differences in phenotypic plasticity exist between the alien and indigenous components of the assemblage. These results are consistent with previous interspecific studies investigating basal thermal tolerance limits and development rates and their phenotypic plasticity, in arthropods, but are inconsistent with results from previous work on desiccation resistance. Thus, for the Collembola, the anticipated advantage of alien over indigenous species under warming and drying is likely to be manifest in local assemblages, globally.

## Introduction

Although assemblages lie within a metacommunity setting, their dynamics are significantly mediated by the physical environments they encounter and by interactions among species. Because physiological traits modulate the effects of the environment on populations (Helmuth *et al*., 2005), knowing the range of trait variation for local assemblages, or significant components of them, can provide much insight into assemblage structure and dynamics (Albert *et al*., 2012; Leibold and Chase, 2018). The development of such understanding is especially important given the growing need to understand the mechanistic basis of a globally common pattern of high local turnover through time without large changes in the richness of assemblages (Blowes *et al*., 2019). Whether such dynamics into the future will include the rising dominance of assemblages by alien species, owing to the expectation that changing climates will generally benefit them (Hulme, 2017), is of much interest given the economic and conservation significance of biological invasions. Such invasions are among the most significant conservation concerns globally (McGeoch and Jetz, 2019).

Assessments of trait variation for significant proportions of the species in local assemblages are uncommon for animals (Chown and Gaston, 2016). Rather, the species compared are typically selected from different localities. Where assemblage investigations have been undertaken, the outcomes can be quite different to those involving interspecific comparisons (compare, for example, Diamond *et al*., 2012 with Kaspari *et al*., 2015). Hence, the general macroecological insight that interspecific and assemblage-level investigations are different and may provide complementary or even contrary insights (Chown and Gaston, 2016).

For plants, explorations of the extent to which indigenous and alien species differ in their characteristics at the assemblage level are increasing (e.g. van Kleunen *et al*., 2018; Mathakutha *et al*., 2019; Sandel and Low, 2019). These comparisons explicitly test the ‘ideal weed’ and ‘plasticity’ hypotheses, proposing that invasion success of a non-native species depends on its specific traits or enhanced phenotypic plasticity, respectively (Enders *et al*., 2020). By contrast, studies exploring whether the indigenous versus alien components of assemblages vary consistently in one or more physiological traits remain rare for animals. Most of the work on trait differences between indigenous and alien species is based on interspecific studies from animals collected across a wide range of localities (e.g. Moyle *et al*., 2013; Bradie and Leung, 2015; Jarošík *et al*., 2015; Allen *et al*., 2017; Janion-Scheepers *et al*., 2018) or for only a small component of a local assemblage (Stachowicz *et al*., 2002; Chown *et al*., 2007). These studies do not consider a range of species from a local setting. Yet, they are frequently used as a basis to forecast rising success of alien species under changing climates (e.g. Janion-Scheepers *et al*., 2018). Indeed, because of the availability of the data, interspecific comparisons remain among the most common macrophysiological approaches adopted. Thus, insights into whether assemblages might be dominated by alien species as climates continue to change, and what mechanisms might lie at the heart thereof, may at best be incomplete and at worst inaccurate. In consequence, much need exists to determine whether predictions made from interspecific studies are borne out at the assemblage level.

Here, we therefore examine the extent to which empirical outcomes from interspecific studies of the trait differences among indigenous and invasive animal species, i.e. tests of the ideal weed and plasticity hypotheses (Enders *et al*., 2020), are borne out by a comprehensive investigation of a local assemblage. We use Collembola as a model group. Springtails are important in belowground systems and mediate aboveground ecological outcomes (Bardgett and van der Putten, 2014). Understanding of physiological trait diversity in the group is growing rapidly (Van Dooremalen *et al*., 2013; Ellers *et al*., 2018; Jensen *et al*., 2019). How this diversity might be partitioned among indigenous and invasive species has been the subject of recent attention at the interspecific level (Janion-Scheepers *et al*., 2018). Recent work has been spurred by concerns over the extent of soil invasions globally, including among Collembola, and by suggestions that anthropogenic change will exacerbate the impacts of invaders on soil systems (Cicconardi *et al*., 2017; Coyle *et al*., 2017; Geisen *et al*., 2019).

We consider five physiological traits that have significant influences on fitness and are therefore frequently incorporated into models of the likely impacts of environmental change on organisms. These are critical thermal minimum and maximum (and the derived trait of tolerance range), desiccation resistance and egg development rate (Birkemoe and Leinaas, 2000; Kearney *et al*., 2013; Sinclair *et al*., 2016; Rozen-Rechels *et al*., 2019). The assemblage is that of sub-Antarctic Macquarie Island. We use this particular springtail assemblage because it is well surveyed both in terms of the species present and their abundances, is characterized by a range of alien species and is representative with regard to Collembola invasions of several islands globally (Cicconardi *et al*., 2017; Baird *et al*., 2019). Moreover, because it is an island assemblage, local factors are likely to be more important in determining dynamics than regional biotic influences (Leibold and Chase, 2018). The general climate of Macquarie Island and its change over the past 40 years are also relatively well understood (Adams, 2009; Bergstrom *et al*., 2015).

Specifically, we test two predictions based on general expectations (Daehler, 2003; Davidson *et al*., 2011; Hulme, 2017; Enders *et al*., 2020) and previous work on springtails (Chown *et al*., 2007; Janion *et al*., 2010; Janion-Scheepers *et al*., 2018). Compared with their indigenous counterparts, alien species should have (**Prediction 1**) greater basal physiological tolerances (for their definition see Chown and Nicolson, 2004) as suggested by the ideal weed hypothesis and (**Prediction 2**) more pronounced phenotypic plasticity as suggested from the phenotypic plasticity hypothesis.

## Materials and methods

### Site description and species sampling

Collembola were collected from Macquarie Island (54°30’ S, 158°57’ E) in March/April of 2016 and 2017 (Supplementary Table S1). The island is small (12 800 ha) with a cool (mean air temperature range 3.8°C to 6.6°C), wet (~954 mm annual precipitation) and windy oceanic climate; vegetation varies from coastal tussock to higher elevation fellfield areas (Selkirk *et al*., 1990).

Springtail collection involved beating into a tray and aspirating individuals from a variety of vegetation types and from the soil surface into 70 ml plastic pots with a saturated Plaster-of-Paris and charcoal powder base (9:1 mixture) (hereafter lined plastic pots). Vegetation from the collection site was placed in to the pots as a food source. Initial sorting into additional lined plastic pots in the laboratory at Macquarie Island was undertaken to initially separate species and ensures densities of ~75–100 animals per pot. Food sources were also placed into these new pots. Turf samples (10 cm^2^ surface area, 5 cm deep) were also collected to ensure that springtails from all layers of soil were included. Springtails in pots and turf samples were maintained at ~5°C and on a 12:12 light:dark (L:D) cycle during the 2-week transportation back to the laboratory in Melbourne. Here, springtails were extracted from turf samples into lined plastic pots over 3 weeks using Berlese–Tullgren funnels.

Springtails were identified using current available keys for Macquarie Island Collembola (e.g. Greenslade, 2006) [further verified with DNA barcoding (see Supplementary Table S2)] and sorted into species. DNA barcoding, involving the extraction and sequencing of 658 bp of the mitochondrial cytochrome oxidase subunit I gene (COI) was undertaken by the Canadian Centre for DNA Barcoding (CCDB, http://www.ccdb.ca/) at the Centre for Biodiversity Genomics, University of Guelph, Canada through the Barcode of Life Data Systems (BOLD, http://www.boldsystems.org/; Ratnasingham and Hebert, 2007) (see also Janion-Scheepers *et al*., 2018). A total of 91 individuals from 16 species was sequenced, with a minimum of three individuals for any one species (Supplementary Table S2). These sequences are available at the BOLD (www.boldsystems.org) under the larger project ‘sub-Antarctic Collembola’. Species were classified as either indigenous to the island or introduced by human activity (alien) based on previously published information (Greenslade, 2006; Phillips *et al*., 2017). Most of the alien species are widespread on the island and hence considered invasive (Terauds *et al*., 2011; Phillips *et al*., 2017).

### Colony maintenance

Springtail colonies were maintained in a controlled temperature room at 10°C ([10.15 ± 0.23°C], verified with iButton Hygrochron® data loggers, Maxim Integrated, San Jose, USA) on a 12:12 L:D cycle. Individuals were maintained by species at intermediate density (75–100 individuals) in 70 ml lined plastic pots to maintain a humid environment (>99% relative humidity). They were fed weekly on a diet of *Platanus* sp. bark (Hoskins *et al*., 2015) to allow for self-selection of nutrients, with the bark also providing some shelter for individuals. Pots were randomly re-arranged in the controlled temperature room during feeding and during experiments to minimize shelf effects.

For experiments measuring thermal and desiccation resistance, springtails were assessed at the F0 and the F2 generations. F0 springtails were used to ensure that as much information on the assemblage could be captured as possible, including springtail species that we failed to rear successfully under laboratory conditions. The F2 generations of springtails were examined to ensure that carry-over effects from the environment of collection, including parental effects, were minimized while also minimizing adaptation to laboratory conditions (Hoffmann and Sgrò, 2017). For investigations of egg development rate, only the F2 generation was used. Eggs were removed weekly from parental pots (F0 individuals) and transferred to new pots to establish the F1 generation. The same process was then used to generate the F2 generation from F1 parents (following Janion-Scheepers *et al*., 2018). In each case, eggs from multiple adults were randomly combined within generations to maintain genetic diversity. F2 springtails reached adulthood between 5 and 16 months after field caught (F0) springtails entered the laboratory.

### Critical thermal limits

Critical thermal limits provide a proxy for survival in active adult organisms (Lutterschmidt and Hutchinson, 1997), including in springtails (e.g. Everatt *et al*., 2013). The critical thermal maxima (*CT_max_*) and critical thermal minima (*CT_min_*) were determined for 16 species of springtails at F0 (9 alien, 7 indigenous, Supplementary Table S3), after they had been held at 10°C for 1 week to examine differences in basal thermal tolerance between the indigenous and alien groups (**Prediction 1**). At the F2 generation, 10 species were investigated (7 alien, 3 indigenous, Supplementary Table S3). These F2 species were also examined for adult (short-term, non-developmental) plasticity in critical thermal limits (**Prediction 2**). Adult phenotypic plasticity was assessed by acclimating F2 springtails to one of five temperature treatments for 7 days prior to experimentation (Supplementary Table S4). Three stable and two variable temperature acclimations were used. Much interest exists in understanding the extent to which fluctuating versus constant temperatures may alter estimates of phenotypic plasticity (Colinet *et al*., 2015). Recently, the importance of the influence of extreme temperature events on the evolution of thermal tolerance has been further emphasized, with the idea that extreme events disproportionately drive changes in such traits (Hoffmann, 2010; Kingsolver and Buckley, 2017). Stable temperatures were set at 5°C, 10°C and 15°C, and variable temperatures were set at 10°C with either a high (25°C) or a low (−5°C) extreme temperature spike that occurred for 1 h each day, with a 30-min temperature ramp up/down either side of the temperature extreme. The temperature spikes were based on extreme event temperatures from a long-term soil surface temperature record for the island (Leihy *et al*., 2018). Acclimation treatments were completed in controlled temperature cabinets (MIR-154-PE, Panasonic, Osaka, Japan) and rooms. Adults were held at the acclimation temperatures for 1 week (following Janion-Scheepers *et al*., 2018; Jensen *et al*., 2019).

Critical thermal limits were determined for individual adult springtails using established protocols (Janion-Scheepers *et al*., 2018). Springtails were contained within custom-built thermal stages (Monash University Instrument Facility, Clayton Campus, VIC, Australia) that were heated or cooled with programmable water baths (Grant Instruments, Cambridge model TXF200) at 0.05°C per minute. This rate was chosen for its environmental relevance, reflecting a commonly encountered rate of temperature change under microclimatic conditions (Allen *et al*., 2016). The floor of the stage was lined with saturated Plaster-of-Paris to minimize desiccation of springtails during experiments, and temperature of the stage floor was recorded using Omega thermometers (model: RDXL 12SD, Omega Engineering, Norwalk, USA) with type K thermocouples. A starting temperature of 10°C (rearing temperature) was used for all experiments. Springtails were observed every ~5°C until a behavioural change occurred (e.g. moving faster/slower) after which they were monitored every 1°C and then every 0.5°C after the *CT_max_*/*CT_min_* was reached for the first individual in the experiment. *CT_max_* and *CT_min_* were defined as the temperature at which a loss of righting response occurred for each individual (Everatt *et al*., 2013; Janion-Scheepers *et al*., 2018). Different sets of individuals were used for the *CT_max_* and *CT_min_* experiments. Three replicates, typically of 10–15 individuals, were completed per species per treatment (sample sizes in Supplementary Table S3). Because variation in critical thermal limits may be affected by differences in body mass (Ribeiro *et al*., 2012), a mean body mass for each species was determined by weighing a random sample of 50 adult individuals of each species using a high-resolution (0.1 μg) microbalance (Mettler-Toledo XP2U, Switzerland) (Supplementary Table S5).

### Desiccation resistance

Desiccation resistance was determined for 10 species of springtails at the F0 generation (5 alien, 5 indigenous, Supplementary Table S3) to examine differences in absolute desiccation resistance between alien and indigenous species (**Prediction 1**). In the F2 generation, 8 species were used (6 alien, 2 indigenous, Supplementary Table S3) to investigate plasticity in desiccation resistance in a cross-tolerance framework with temperature (**Prediction 2**). Short-term temperature acclimation has previously been shown to alter desiccation resistance in indigenous and alien springtails unequally, to the alien species’ advantage (Chown *et al*., 2007). Here, the effects of short-term temperature acclimations on desiccation resistance were examined at two acclimation and two test temperatures. F2 springtails were acclimated at either 10°C or 20°C in controlled temperature rooms for 7 days prior to the desiccation experiment that was conducted at either 10°C or 20°C.

An experimental protocol for desiccation resistance, measured as survival time at 76% relative humidity, was established based on previous methods (Kærsgaard *et al*., 2004; Chown *et al*., 2007). Individual springtails were contained within glass vials covered with fine mesh, which were then housed in sealed, 70 ml plastic pots containing 15 ml of saturated NaCl solution as a desiccant. Saturated NaCl was used as it provides a consistent relative humidity of 76% from 0°C to 20°C. Furthermore, it has been shown that springtails can survive between 1 and 24 h at this humidity (Chown *et al*., 2007). Each pot contained two glass vials with ~5 springtails per vial and an iButton Hygrochron® data logger (Maxim Integrated, San Jose, USA) to verify temperature and relative humidity. Throughout the experiment, conducted in controlled-temperature rooms, springtails were examined every 10 min under a Leica M80 microscope (Leica Microsystems Pty Ltd, Wetzlar, Germany), and time to death (minutes) was recorded for each individual. Typically, four replicates of 10 individuals were used per experiment, with some exceptions for F0 experiments due to low numbers of springtails available (see Supplementary Table S3 for sample sizes). Following the experiment, springtails were dried at 40°C for 24 h and then weighed in groups by replicate using a high-resolution (0.1 μg) microbalance (Mettler-Toledo XP2U, Switzerland) to obtain an estimate of individual dry body mass.

### Egg development and hatching success

Egg development time and hatching success were determined for eight species, including six alien and two indigenous species (Supplementary Table S3), at seven temperatures ranging from 0°C–30°C, in 5°C increments (Supplementary Table S4) (**Predictions 1 and 2**) following previous protocols (Birkemoe and Leinaas, 2000; Janion *et al*., 2010). Eggs laid by F2 adults at 10°C were collected and transferred to each respective development temperature within 24 h of laying. Eggs were transferred to 70 ml lined pots and kept in controlled temperature cabinets (MIR-154-PE, Panasonic) or rooms for the duration of development. Five replicate pots per temperature with 10 eggs per pot were used to provide a sample size of 50 eggs per temperature for each species. Eggs were checked daily for hatching. Days to hatching for each egg, and hatching success, measured as a percentage of eggs hatched within each pot, were recorded. Eggs were classified as unviable/dead if they were either visibly dead (shrivelled, dissolved or extremely discoloured) or if they had not hatched within 10 days (at 10°C–30°C) or within 14 days of the previously hatched egg within the same pot (at 0°C and 5°C).

### Statistical analyses

All analyses were undertaken using R v. 3.5.2 (R Core Team, 2018) implemented in R Studio v. 1.1.463. All code and data files are archived in the Monash Figshare repository (doi: http://dx.doi.org/10.26180/5e17b874b125c and doi: http://dx.doi.org/10.26180/5e17c3bc55197).

### Critical thermal limits

To test **Prediction 1**, basal critical thermal limit differences (*CT_max_*, *CT_min_*, *CT_range_* [difference between species mean *CT_max_* and mean *CT_min_*]) among alien and indigenous species at the F0 generation (10°C acclimation) were assessed using two approaches. The first explored differences in *CT_max_* and *CT_min_* among individuals from the two groups excluding species identity, assuming that individual trait variation is important in a community dynamics context (Albert *et al*., 2012), using a linear model with status as a fixed factor. Because individuals were not weighed, mass was not included as a covariate. Differences between the alien and indigenous groups were then assessed using species means (or differences in means for *CT_range_*) within a phylogenetically explicit framework using phylogenetic generalized least squares (PGLSs) (Garland and Ives, 2000), as implemented in the caper v. 0.5.2 package (Orme *et al*., 2013), including species mean mass as a covariate. Given small numbers of species, a Brownian motion model of evolution was used (Cooper *et al*., 2016) and a maximum likelihood approach estimated Pagel’s λ (Pagel, 1999) to indicate the degree of phylogenetic influence in the data. The phylogenetic tree used for the analyses was based on Janion-Scheepers *et al*. (2018) and with mtCOI data used to infer species relationships. For the final tree, branch lengths were assigned using Grafen’s method (Grafen, 1989), and the tree (Supplementary Figure S1) is available as a Newick file in the Monash Figshare repository (doi: http://dx.doi.org/10.26180/5e17b874b125c). Density plots made using the package ggplot2 were used to illustrate the range of variation in *CT_max_* and *CT_min_* for each species and across individuals in the full assemblage investigated.

Because differences in traits among F0 and F2 adults might arise for various reasons (Hoffmann and Sgrò, 2017), means of the critical thermal limit traits in the F0 and F2 generations, each acclimated for 1 week at 10°C, were compared among the 10 species common to both sets of trials. A PGLS approach using a reduced tree was initially used. Because Pagel’s λ was estimated as zero for *CT_max_*, *CT_min_* and *CT_range_* and because of the likely measurement variation of the traits, a ranged major axis model (RMA, Legendre and Legendre, 2012), implemented in the lmodel2 package, was used for each trait to determine whether the slope differed from 1 and the intercept from zero in each case by examining the 95% confidence intervals of the estimated values.

To test **Prediction 2**, the effects of acclimation to 5°C, 10°C and 15°C were examined for the F2 *CT_max_* and *CT_min_* trials by calculating an acclimation response ratio (ARR) (°C/°C) (Gunderson and Stillman, 2015). The ARR was calculated from the slope of the intraspecific relationship between acclimation temperature and critical thermal limits trait for each of the 10 species investigated based on individual data for each acclimation temperature. Systematic differences between the indigenous and alien species were investigated using a PGLS approach as above.

The impacts of a high (25°C) or low (−5°C) temperature spike for 1 h on a daily basis as an extreme event acclimation treatment were compared for each of the 10 species using linear models with temperature as a fixed factor and Tukey’s honest significant difference (Crawley, 2013).

### Desiccation resistance

Desiccation resistance was measured as individual survival time resulting in data that are bounded to the left at zero and right skewed (Supplementary Figure S2). To assess whether time to death differed between the F0 and F2 generations, five species for which F0 and F2 data were available at both acclimation and test temperatures of 10°C were each compared using a generalized linear model (GLM) assuming a quasipoisson distribution and a log link function because of the form of the data (O’Hara and Kotze, 2010). Because substantial differences between the F0 and F2 generations were found in one of the species, data from the F0 and F2 generations were not pooled for comparisons among indigenous and alien species, even though the two data series did not overlap completely in the available species.

To test **Prediction 1**, comparisons of the indigenous and alien species were made in two ways using the F0 data, in keeping with the previous approach. In the first, a GLM (assuming a quasipoisson distribution and a log link function) was used to compare the alien and indigenous assemblages (fixed factor), including an estimate of log_10_ dry body mass for each individual (from the individuals weighed at the end of the study) as a covariate. Thereafter, differences between the alien and indigenous groups were assessed using species means (here log_10_ of time to death to account for the skew in the data) using PGLS as described above, including species log_10_ mean dry mass as a covariate.

To test **Prediction 2**, the effects of thermal acclimation (fixed factor) on desiccation resistance were analysed for each species separately using a GLM assuming a quasipoisson distribution and a log link function. Acclimation at higher temperatures was expected to afford an extended survival time to the alien but not the indigenous species (Chown *et al*., 2007). Patterns of acclimation were compared visually for each of the groups and then status (alien or indigenous) included in a model (as above) with all species.

**Table 1:** Means and standard deviations for critical thermal limits at the assemblage level, including linear model comparison outcomes and species-level means, standard deviations and ranges for ARRs

**Assemblage level critical thermal limits**					
	**Indigenous**	**Alien**	**Linear model outcomes**		
	Mean ± SD	Mean ± SD	F	df	*P*
*CT_max_* (°C)	31.9 ± 2.0	36.1 ± 2.6	397.3	1512	<0.0001
*CT_min_* (°C)	−2.8 ± 1.0	−3.9 ± 1.6	86.7	1528	<0.0001
**Species critical thermal limit ARRs**					
	**Indigenous**	**Alien**			
	**Mean ± SD (range)**	**Mean ± SD (range)**			
ARR *CT_max_* (°C/°C)	0.049 ± 0.041	0.001 ± 0.017			
	(0.014–0.095)	(−0.018–0.027)			
					
ARR *CT_min_* (°C/°C)	0.070 ± 0.045	0.062 ± 0.037			
	(0.025–0.120)	(0.011–0.117)			

**Figure 1:**
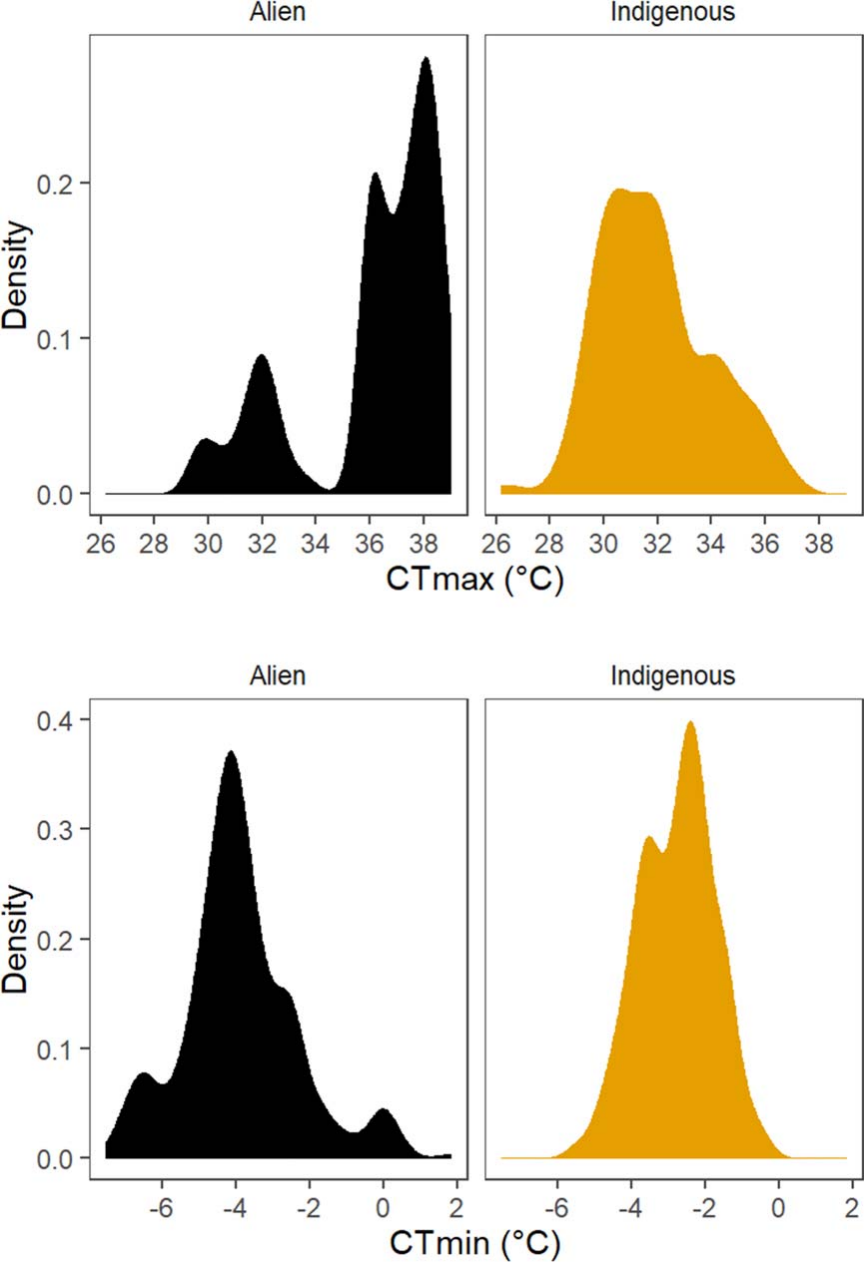
Density plots of thermal tolerance in individuals. (**A**) *CT_max_* and (**B**) *CT_min_* for the indigenous and alien assemblages of springtails from Macquarie Island measured in the F0 generation after 7 days at 10°C acclimation

### Egg development and hatching success

Egg development times for individuals of each species at each temperature were converted to development rates (1/days to hatching). Because single individuals were not examined across a range of temperatures (different eggs were assessed at each temperature), a function-valued trait approach (Gomulkiewicz *et al*., 2018) was not implemented. Mean values for development rate were obtained for each species at each temperature and plotted against temperature.

**Table 2:** Critical thermal limits for springtail species from Macquarie Island

**Species**	***n***	***CT***_***max***_ **± SD**	***n***	***CT***_***min***_ **± SD**	**CT**_***range***_
**Alien**					
*C. denticulata*	31	37.8 ± 0.4	30	−4.4 ± 0.7	42.3
*Desoria tigrina*	34	32.0 ± 0.5	35	−2.8 ± 0.6	34.8
*H. purpurescens*	31	36.1 ± 0.3	31	−4.5 ± 0.7	40.6
*H. viatica*	42	38.3 ± 0.5	51	−5.7 ± 1.2	44.0
*Lepidocyrtus* sp*.* nr. *violaceus*	30	37.8 ± 0.7	31	−3.6 ± 0.9	41.4
*Megalothorax* nr. *minimus*	29	31.0 ± 1.4	28	−0.6 ± 1.0	31.6
*Parisotoma notabilis*	31	36.2 ± 0.4	30	−3.7 ± 0.5	39.9
*Protaphorura fimata*	33	38.0 ± 0.5	33	−4.5 ± 0.5	42.5
*Proisotoma* sp.	32	37.0 ± 0.7	40	−3.8 ± 0.8	40.8
**Indigenous**					
*Folsomotoma punctata*	35	30.5 ± 1.2	35	−2.5 ± 0.4	33.0
*Katianna banzarei*	32	29.7 ± 0.6	29	−1.6 ± 0.5	31.3
*Lepidobrya mawsoni*	28	31.4 ± 1.1	33	−2.1 ± 0.8	33.5
*Mucrosomia caeca*	31	33.8 ± 0.8	30	−3.2 ± 0.6	37.0
*P. insularis*	33	31.3 ± 0.8	34	−3.6 ± 0.6	34.9
*Sminthurinus* cf. *tuberculatus*	31	35.3 ± 1.0	30	−3.7 ± 0.9	38.9
*Tullbergia bisetosa*	31	31.4 ± 1.1	30	−2.7 ± 1.2	34.1

*CT_max_*: critical thermal maximum; *CT_min_*: critical thermal minimum; *CT_range_*: mean *CT_max_* minus mean *CT_min_*; SD: standard deviation.

**Table 3:** Outcomes of the PGLSs analyses for assessment of differences between the indigenous and alien species groups for the traits investigated in this study. In each case, the difference between the alien and indigenous species groups are shown [status (indigenous)], and the full model statistics provided including a maximum likelihood estimate of Pagel’s λ (_ML_λ). ARR = acclimation response ratio

**CT values**	**Estimate ± S.E.**	**t**	***P***
***Thermal tolerance***			
***CT***_***max***_			
Intercept	36.02 ± 0.80	44.92	<0.0001
Status (indigenous)	−4.11 ± 1.21	−3.39	0.0045
	F_(1,14)_ = 11.47, R^2^ = 0.41, _ML_λ = 0.00		
***CT***_***min***_			
Intercept	−3.42 ± 0.71	−4.60	0.0004
Status (indigenous)	0.84 ± 0.45	1.87	0.083
	F_(1,14)_ = 3.49, R^2^ = 0.14, _ML_λ = 0.75		
***CT***_***range***_			
Intercept	39.76 ± 1.15	34.47	<0.0001
Status (indigenous)	−5.07 ± 1.74	−2.91	0.011
	F_(1,14)_ = 8.47, R^2^ = 0.33, _ML_λ = 0.00		
**ARR *CT***_***max***_			
Intercept	0.0076 ± 0.0097	0.784	0.456
Status (indigenous)	0.0410 ± 0.0176	2.323	0.049
	F_(1,8)_ = 5.40, R^2^ = 0.33, _ML_λ = 0.00		
**ARR *CT***_***min***_			
Intercept	0.0622 ± 0.0151	4.105	0.003
Status (indigenous)	0.0080 ± 0.0277	0.290	0.779
	F_(1,8)_ = 0.08, R^2^ = 0.0, _ML_λ = 0.00		
**Desiccation**			
**Log**_**10**_ **time to death**			
Intercept	2.265 ± 0.154	14.686	<0.0001
Status (indigenous)	−0.601 ± 0.244	−2.464	0.039
	F_(1,8)_ = 6.07, R^2^ = 0.360, _ML_λ = 0.00		
**Development rate**			
**Slope**			
Intercept	0.0049 ± 0.0005	10.131	<0.0001
Status (indigenous)	−0.0008 ± 0.0010	−0.863	0.422
	F_(1,6)_ = 0.774, R^2^ = 0.0, _ML_λ = 0.00		
**Slope (eV)**			
Intercept	0.754 ± 0.052	14.613	<0.0001
Status (indigenous)	0.140 ± 0.103	1.360	0.223
	F_(1,6)_ = 1.850, R^2^ = 0.108, _ML_λ = 0.00		
***T***_***opt***_			
Intercept	23.333 ± 1.128	20.679	<0.0001
Status (indigenous)	−5.833 ± 2.257	−2.585	0.041
	F_(1,6)_ = 6.682, R^2^ = 0.448, _ML_λ = 0.00		
***U***_***max***_			
Intercept	0.103 ± 0.011	9.621	<0.0001
Status (indigenous)	−0.040 ± 0.021	−1.878	0.109
	F_(1,6)_ = 3.527, R^2^ = 0.265, _ML_λ = 0.00		
**Hatching success (ULT50)**			
Intercept	23.567 ± 0.943	24.989	<0.0001
Status (indigenous)	−5.617 ± 1.886	−2.978	0.025
	F_(1,6)_ = 8.867, R^2^ = 0.529, _ML_λ = 0.00		

*CT_max_*: critical thermal maximum; *CT_min_*: critical thermal minimum; *CT_range_*: mean *CT_max_* minus mean *CT_min_*; *T_opt_*: optimum temperature: *U_max_*: development rate at the optimum temperature; S.E.: standard error.

**Table 4:** Outcomes of a linear model examining the effects on *CT_max_* and *CT_min_* of week-long acclimation treatments of 5°C, 10°C, 15°C, 1 h at −5°C per day (with a background temperature of 10°C) and 1 h per day at 25°C (with a background temperature of 10°C). The full model outcome is shown, along with Tukey HSD contrasts for the −5°C extreme vs. 5°C and the 25°C extreme vs. 15°C (for boxplots, see Supplementary Figs S4 and S5). The error values are standard error

**Species**	***CT***_***max***_		
**Alien**	**−5**°**C extreme vs. 5**°**C**	**25**°**C extreme vs. 15**°**C**	**Full model statistics**
*C. denticulata*	−0.4 ± 0.1°C, t = −2.8, *P* = 0.050	−0.2 ± 0.1°C, t = −1.4, *P* = 0.650	F_(4,186)_ = 2.73, *P* = 0.030
*H. purpurescens*	0.1 ± 0.1°C, t = 1.4, *P* = 0.612	0.3 ± 0.1°C, t = 3.0, *P* = 0.026	F_(4,180)_ = 7.20, *P* < 0.0001
*H. viatica*	−0.02 ± 0.1°C, t = −0.1, *P* = 1.0	0.1 ± 0.1°C, t = 1.3, *P* = 0.682	F_(4,157)_ = 1.63, *P* = 0.168
*L.* nr. *violaceus*	−0.4 ± 0.1°C, t = −2.9, *P* = 0.037	0.5 ± 0.1°C, t = 3.6, *P* = 0.003	F_(4,156)_ = 18.68, *P* < 0.0001
*P. notabilis*	−0.1 ± 0.1°C, t = −1.6, *P* = 0.523	−0.01 ± 0.1°C, t = −0.1, *P* = 1.0	F_(4,185)_ = 0.62, *P* = 0.648
*P. fimata*	0.3 ± 0.1°C, t = 2.9, *P* = 0.038	0.2 ± 0.1°C, t = 1.5, *P* = 0.540	F_(4,186)_ = 5.49, *P* = 0.001
*Proisotoma* sp.	−0.7 ± 0.2°C, t = −4.3, *P* < 0.001	−0.4 ± 0.2°C, t = −2.3, *P* = 0.158	F_(4,192)_ = 10.52, *P* < 0.0001
**Indigenous**			
*M. caeca*	0.4 ± 0.1°C, t = 3.0, *P* = 0.026	−0.3 ± 0.1°C, t = −2.3, *P* = 0.159	F_(4,195)_ = 16.22, *P* < 0.0001
*P. insularis*	0.2 ± 0.1°C, t = 1.5, *P* = 0.568	0.2 ± 0.1°C, t = 1.7, *P* = 0.450	F_(4,154)_ = 5.83, *P* < 0.001
*T. bisetosa*	−0.3 ± 0.2°C, t = −1.3, *P* = 0.686	0.1 ± 0.2°C, t = 0.3, *P* = 0.998	F_(4,154)_ = 1.67, *P* = 0.159
**Species**	***CT***_***min***_		
**Alien**	**−5**°**C extreme vs. 5**°**C**	**25**°**C extreme vs. 15**°**C**	
*C. denticulata*	0.3 ± 0.2°C, t = 1.3, *P* = 0.711	−0.1 ± 0.2°C, t = −0.6, *P* = 0.972	F_(4,176)_ = 5.65, *P* < 0.001
*H. purpurescens*	0.02 ± 0.2°C, t = 0.1, *P* = 1.0	−0.3 ± 0.2°C, t = −1.7, *P* = 0.469	F_(4,173)_ = 0.84, *P* = 0.503
*H. viatica*	0.3 ± 0.2°C, t = 1.5, *P* = 0.576	−0.3 ± 0.2°C, t = −1.6, *P* = 0.513	F_(4,158)_ = 2.45, *P* = 0.048
*L.* nr. *violaceus*	0.4 ± 0.1°C, t = 2.6, *P* = 0.085	−0.4 ± 0.2°C, t = −2.7, *P* = 0.059	F_(4,155)_ = 17.83, *P* < 0.0001
*P. notabilis*	−0.6 ± 0.1°C, t = −5.6, *P* < 0.0001	−0.4 ± 0.1°C, t = −4.1, *P* < 0.001	F_(4,179)_ = 28.3, *P* < 0.0001
*P. fimata*	0.3 ± 0.1°C, t = 2.5, *P* = 0.104	−0.4 ± 0.1°C, t = −3.0, *P* = 0.026	F_(4,181)_ = 11.01, *P* < 0.0001
*Proisotoma* sp.	−0.1 ± 0.1°C, t = −1.2, p = 0.742	0.2 ± 0.1°C, t = 1.5, *P* = 0.582	F_(4,196)_ = 6.21, *P* = 0.0001
**Indigenous**			
*M. caeca*	0.7 ± 0.1°C, t = 5.6, *P* < 0.0001	−0.1 ± 0.1°C, t = −1.0, *P* = 0.847	F_(4,190)_ = 25.36, *P* < 0.0001
*P. insularis*	−0.3 ± 0.1°C, t = −2.6, *P* = 0.089	−0.2 ± 0.1°C, t = −1.3, *P* = 0.699	F_(4,155)_ = 5.40, *P* < 0.001
*T. bisetosa*	0.6 ± 0.2°C, t = 3.1, *P* = 0.020	0.1 ± 0.2°C, t = 0.5, *P* = 0.985	F_(4,148)_ = 4.77, *P* = 0.001

Maximum development rate (*U_max_*) and the temperature at which this rate was realized (*T_opt_*) were extracted from the means data. **Prediction 1** was tested by inspecting the curves and selecting the appropriate mean values and temperatures, following previous approaches which have sought not to fit curves to the empirical data (Jarošík *et al*., 2015; Sørensen *et al*., 2018). Further to test **Prediction 1**, hatching success (as a proportion) was plotted against rearing temperature. This revealed that hatching success did not decline to zero at the lowest temperatures investigated in all of the species. Therefore, low temperature variation in hatching success was not investigated. Rather only the high temperature at which hatching success declined to 50% of the sample population, known as the upper lethal temperature 50 (ULT50) was estimated using a GLM assuming a binomial distribution and using a logit link function, with ULT50 values calculated from the fitted models using the mass package (Crawley, 2013).

To test **Prediction 2**, for each species the slope of the relationship or the temperature sensitivity of development was calculated in two ways. First, a linear model was used to estimate the slope of the relationship between mean rate (1/days to hatching) at a given temperature and that temperature (°C) for each species. Data above the optimum temperature of the relationship [i.e. the temperature at maximum rate (see Sørensen *et al*., 2018)] were not used. Second, following a range of previous approaches (e.g. Dell *et al*., 2011), the natural logarithm of rate was plotted against 1/kT, where k = Boltzmann’s constant (8.617^*^10^−5^ ev.K^−1^) and T is temperature in Kelvin.

A PGLS approach, as described previously, was implemented to investigate differences between alien (six species) and indigenous groups (two species) in each of these four traits (slope, *U_max_*, *T_opt_*, ULT50). Here, Pagel’s λ was always estimated as zero. Because one of the alien species, *Hypogastrura purpurescens*, was found to be quite different to the others with regards to these variables, linear models used to assess differences between the alien and indigenous groups excluded this species.

## Results

### Critical thermal limits

The alien assemblage had, on average, a higher *CT_max_* and lower *CT_min_* than the indigenous assemblage (Table 1), although the alien assemblage was bimodal for *CT_max_* (Fig. 1), largely owing to low values for *Proisotoma* sp. (Supplementary Figure S3). The PGLS models, based on species means (Table 2), revealed significant and substantial differences among the alien and indigenous species in *CT_max_* (4.1°C) and *CT_range_* (5.1°C), but not in *CT_min_*, with substantial phylogenetic signal in *CT_min_* only (Table 3). Species mean mass was not a significant covariate for any of the traits and was omitted in the final models.

The F0 and F2 generation data did not differ among the 10 species as indicated by the slopes and intercepts of the RMA regressions not being different from 1 and 0, respectively, for *CT_max_* (slope: 1.0, 95% C.I.s: 0.91 to 1.10; intercept: 0.24, 95% C.I.s: −3.43 to 3.57), *CT_min_* (slope: 0.87, 95% C.I.s: 0.51 to 1.47; intercept: −0.65, 95% C.I.s: −2.04 to 1.65) and *CT_range_* (slope: 0.98, 95% C.I.s: 0.82 to 1.17; intercept: 1.21, 95% C.I.s: −6.12 to 7.40).

ARRs (in °C/°C) did not differ between the alien and indigenous groups for *CT_min_*, and only marginally so for *CT_max_* (Tables 1, 3). Thus, the ARR for these two traits is similar for the two groups of species, though the ARR for *CT_min_* (0.065 ± 0.038°C/°C) is significantly larger than the ARR for *CT_max_* (0.020 ± 0.031°C/°C) (linear model F_(1,18)_ = 8.29, *P* = 0.01) (summary data in Supplementary Table S6).

The extreme event treatments of either a low (−5°C) or high (25°C) temperature spike for 1 h each day had limited and variable effects across the species, especially compared with either the constant 5°C acclimation in the former case and the constant 15°C in the latter (Table 4; Supplementary Figures S4 and S5).

### Desiccation resistance

In four of the five species for which data were available for both F0 and F2, no differences in time to death were found between the generations (GLM: *Ceratophysella denticulata* t = −0.788, *P* = 0.433; *Protaphorura fimata* t = 0.592, *P* = 0.556; *Mucrosomia caeca* t = −0.296, *P* = 0.768; *Parisotoma insularis* t = 0.363, *P* = 0.718), whereas in the fifth, *Proisotoma* sp., the F2 generation had a substantially and significantly longer time to death than the F0 generation (F0: 80 ± 32 min (median = 80), F2: 99 ± 28 min (median = 90); t = 2.99, *P* = 0.005). Thus, for the remainder of the investigations, the F0 and F2 generations were analysed separately.

**Figure 2:**
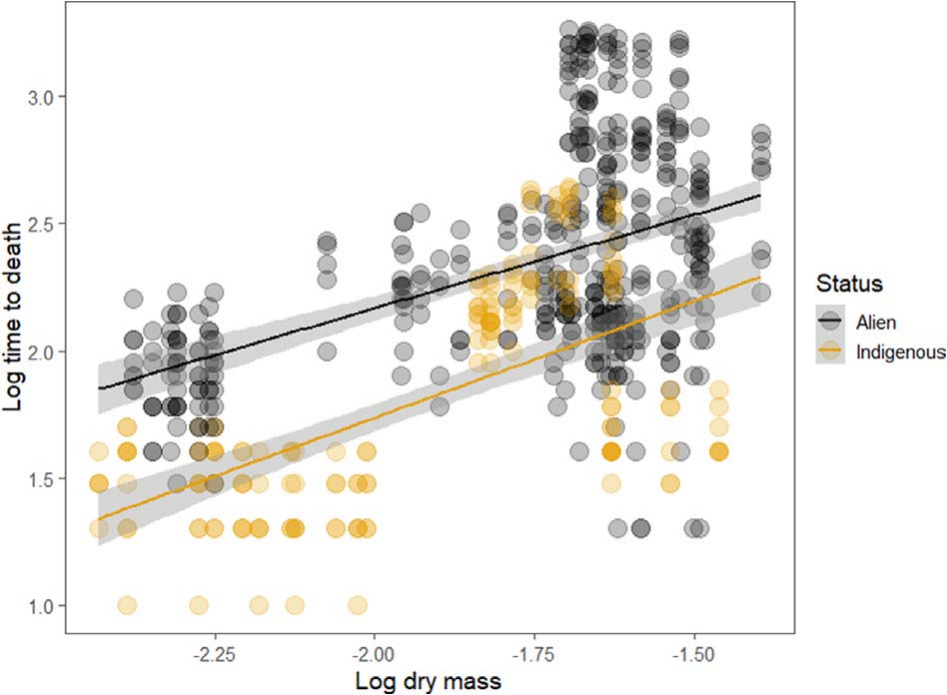
Relationship between time to death (log10, minutes) and dry body mass (log10, mg) for individuals of the F0 generation of springtail species from Macquarie Island subject to desiccation trials, indicating substantially greater desiccation resistance, on average, of the alien over the indigenous species. The fitted lines are from a linear model fitted in ggplot2

For the F0 generation and using individual data, large and significant differences were found between the alien and indigenous species in time to death [alien mean: 326 ± 413 min (median: 140); indigenous mean: 115 ± 112 (median 60)], including with dry mass as a covariate (Table 5; Fig. 2). The PGLS models using species means also showed significant and substantial differences among the alien and indigenous species in time to death but with no phylogenetic signal in the data (Tables 3, 6).

**Figure 3:**
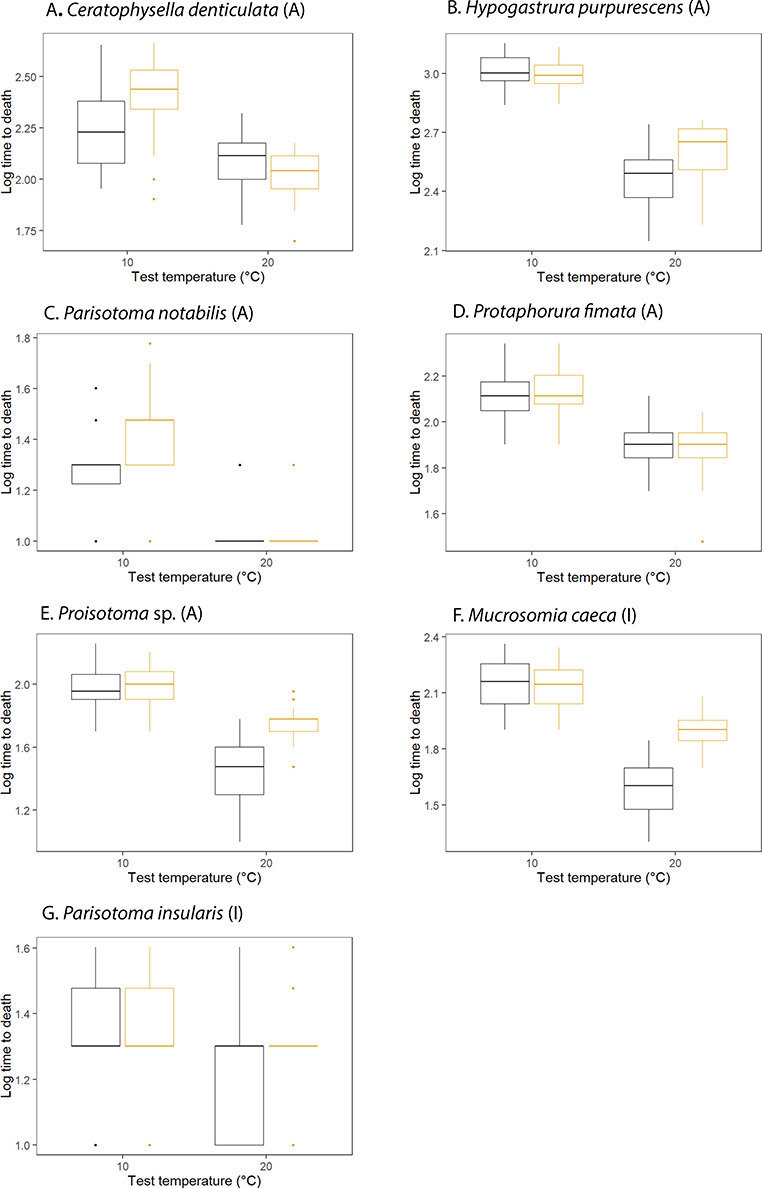
Boxplots illustrating the effects of different acclimation treatments [10°C (black) or 20°C (orange) for 1 week] on desiccation resistance (provided here as log_10_ time to death in minutes) measured under 76% relative humidity at a test temperature of either 10°C or 20°C. Summary data are available in Supplementary Table S9

Acclimation treatments in the F2 generation revealed that, as expected, time to death was shorter at the higher test temperatures, but that pre-exposure to an acclimation of 20°C frequently resulted in improved desiccation resistance either at 20°C (three species) or at 10°C (two species), although in two species no effects of acclimation were found (Fig. 3; Table 7). Similar responses were found among the alien and in the indigenous species [e.g. in Fig. 3 compare *Proisotoma* sp. (alien) with *M. caeca* (indigenous)], with the GLM supporting this interpretation given no interactions among status, acclimation and test temperatures ( Supplementary Table S7).

### Egg development and hatching success

**Figure 4:**
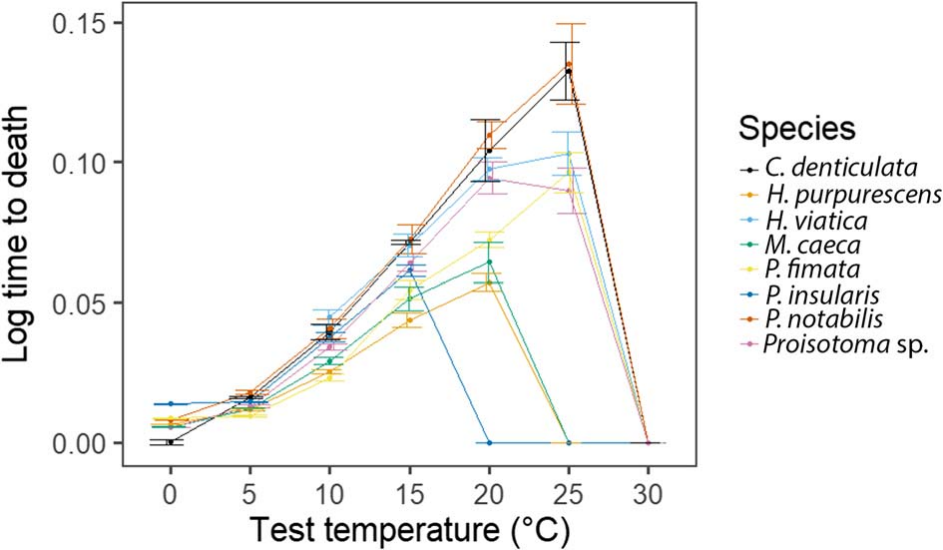
Mean egg development rate (1/days to hatching) between 0°C and 30°C for each of the species investigated here (indigenous species are *M. caeca* and *P. insularis*, the remainder are alien). Where values are zero, this typically indicates no development or very low hatching success with some development in the case of the values at 0°C. Summary data in Supplementary Table S10

Despite considerable differences in the form of the development rate–temperature curves among species (Fig. 4) in the PGLS analyses only *T_opt_* differed between the alien and indigenous groups, with indigenous species having the lower value (Table 3). Excluding *H. pupurescens*, an outlier among the alien species (Table 8), resulted in rate-temperature slopes which still did not differ between the groups [slope: estimate (indigenous) = −0.0013 ± 0.0006, t = −2.34, *P* = 0.066; slope eV: estimate (indigenous) = 0.138 ± 0.116, t = 1.196, *P* = 0.285]. Both *T_opt_* [estimate (indigenous) = −6.5 ± 2.1, t = −3.047, *P* = 0.029] and *U_max_* [estimate (indigenous) = −0.050 ± 0.015, t = −3.304, *P* = 0.021] were, however, lower in the indigenous than in the alien species group.

In all of the species, hatching success had declined to zero by 30°C, the highest temperature investigated. On average, the temperature at which hatching success had declined to 50% (ULT50) (Table 8), was significantly lower (by ~5.7°C) for the indigenous than for the alien species (Table 3), with *H. purpurescens* an outlier among the alien species.

## Discussion

In this springtail assemblage from Macquarie Island, the outcomes of the tests of the two predictions are clear. **Prediction 1** [from the ideal weed hypothesis (Enders *et al*., 2020)], of greater basal physiological tolerance in the alien than in the indigenous species, is supported. On average, *CT_max_* is higher, *CT_range_* is broader, desiccation resistance is greater and egg development *T_opt_* and *U_max_* and the ULT50 for egg hatching success are higher in the alien than in the indigenous species. Only *CT_min_* is indistinguishable between these two groups at the species level. At the individual level, however, the difference in *CT_min_* between alien and indigenous species is clear. By contrast, **Prediction 2**, of greater phenotypic plasticity in the alien than in the indigenous species [from the phenotypic plasticity hypothesis (Enders *et al*., 2020)] is not supported. Acclimation responses for *CT_max_*, *CT_min_* and desiccation resistance and the slopes of the rate-temperature relationships for egg development do not differ between the alien and indigenous species groups.

**Table 5:** Outcome of a generalized linear model (quasipoisson distribution, log link) comparing individual time to death following desiccation among the alien and indigenous assemblages for the F0 generation trial

	**Estimate ± S.E.**	**t**	***P***
Intercept	9.748 ± 0.512	19.030	<0.0001
Status (indigenous)	−0.892 ± 0.136	−6.537	<0.0001
Log_10_ dry mass	2.265 ± 0.299	7.579	<0.0001

Residual deviance 63 376; df = 323; quasipoisson dispersion parameter = 236.2617

**Table 6:** Mean time to death under desiccating conditions of 76% humidity at 10°C after acclimation for 1 week at 10°C for the springtail species investigated here. Data for the F0 generation are shown with the exception of two species [marked (F2)]

**Species**	**n**	**Mean ± SD**	**Median**	**Range**
**Alien**				
*C. denticulata*	30	205 ± 85	180	100–380
*Desoria tigrina*	26	116 ± 27	120	40–160
*H. purpurescens* (F2)	40	1052 ± 203	1005	690–1420
*H. viatica*	36	1064 ± 402	970	420–1740
*Lepidocyrtus* sp. nr. *violaceus*	21	233 ± 111	260	40–410
*Parisotoma notabilis* (F2)	36	20 ± 8	20	10–40
*P. fimata*	32	132 ± 35	135	50–190
*Proisotoma sp.*	38	80 ± 32	80	30–170
**Indigenous**				
*M. caeca*	34	149 ± 34	140	90–220
*P. insularis*	23	24 ± 9	20	10–40
*Sminthurinus* cf. *tuberculatus*	30	285 ± 103	255	150–440
*T. bisetosa*	26	47 ± 13	40	30–70

Differences in basal tolerance, but not in phenotypic plasticity, of critical thermal limits specifically, are largely in keeping with previous work. The most extensive interspecific study to date (Janion-Scheepers *et al*., 2018) found that indigenous springtail species are characterized by critical thermal maxima that are on average 3°C lower than those of their alien counterparts and a *CT_range_* difference of about the same magnitude, with no difference in *CT_min_* and ARR between the groups. In this Macquarie Island assemblage, the differences in *CT_max_* and *CT_range_* are slightly larger (4°C and 5°C, respectively), but otherwise the findings accord closely. Moreover, irrespective of the acclimation conditions, we were unable to effect much change in the value of *CT_max_* in any of the species we investigated, reflected also by the low ARR values for this trait. Longer-term laboratory selection experiments have also been unable to do so in both alien and indigenous springtail species (Janion-Scheepers *et al*., 2018).

**Table 7:** Outcomes of the generalized linear models (quasipoisson distribution, log link) estimating the effects of acclimation and treatment temperature on time to death (as a measure of desiccation resistance) in each of the species examined in this study

**Species**	**Estimate ± S.E.**	**t**	***P***
**Alien**			
*C. denticulata*			
Intercept	5.245 ± 0.049	107.72	<0.0001
Acclimation (20)	0.371 ± 0.064	5.812	<0.0001
Test temperature (20)	−0.379 ± 0.077	−4.899	<0.0001
Acclimation: test	−0.566 ± 0.116	−4.874	<0.0001
Residual deviance 3702.8; df = 171; quasipoisson dispersion parameter = 21.57			
*H. purpurescens*			
Intercept	6.958 ± 0.029	241.82	<0.0001
Acclimation (20)	−0.047 ± 0.042	−1.122	0.264
Test temperature (20)	−1.211 ± 0.061	−19.964	<0.0001
Acclimation: test	0.330 ± 0.082	4.020	<0.0001
Residual deviance 5442.4; df = 152; quasipoisson dispersion parameter = 34.82			
*Parisotoma notabilis*			
Intercept	2.996 ± 0.064	46.94	<0.0001
Acclimation (20)	0.353 ± 0.079	4.449	<0.0001
Test temperature (20)	−0.553 ± 0.102	−5.414	<0.0001
Acclimation: test	−0.393 ± 0.140	−2.803	0.006
Residual deviance 448.0; df = 156; quasipoisson dispersion parameter = 2.932			
*P. fimata*			
Intercept	4.919 ± 0.031	160.73	<0.0001
Acclimation (20)	0.002 ± 0.043	0.040	0.968
Test temperature (20)	−0.556 ± 0.050	−11.142	<0.0001
Acclimation: test	−0.008 ± 0.070	−0.115	0.908
Residual deviance 754.9; df = 155; quasipoisson dispersion parameter = 4.87			
*Proisotoma* sp.			
Intercept	4.603 ± 0.038	120.16	<0.0001
Acclimation (20)	0.036 ± 0.053	0.681	0.497
Test temperature (20)	−1.152 ± 0.081	−14.192	<0.0001
Acclimation: test	0.547 ± 0.103	5.299	<0.0001
Residual deviance 1046.2; df = 165; quasipoisson dispersion parameter = 6.294			
**Indigenous**			
*M. caeca*			
Intercept	4.985 ± 0.035	144.55	<0.0001
Acclimation (20)	−0.033 ± 0.049	−0.673	0.502
Test temperature (20)	−1.303 ± 0.078	−16.78	<0.0001
Acclimation: test	0.757 ± 0.097	7.796	<0.0001
Residual deviance 1168.7; df = 159; quasipoisson dispersion parameter = 7.30			
*P. insularis*			
Intercept	3.209 ± 0.056	57.27	<0.0001
Acclimation (20)	0.0001 ± 0.0001	0.0	1.0
Test temperature (20)	−0.253 ± 0.085	−2.957	0.004
Acclimation: test	0.002 ± 0.121	0.097	0.923
Residual deviance 479.9; df = 152; quasipoisson dispersion parameter = 3.107			

These findings of limited plasticity and adaptability in *CT_max_* over the shorter term are in keeping with previous investigations of other organisms (Hoffmann *et al*., 2013; Gunderson and Stillman, 2015; MacLean *et al*., 2019). Such similarity does not help to explain, however, why such substantial interspecific variation exists in the basal *CT_max_* of springtails. Here, for example, the largest difference in *CT_max_* among species is 8.6°C, whereas the largest difference in *CT_min_* is 5.1°C. In the broad-scale interspecific study (Janion-Scheepers *et al*., 2018), *CT_max_* varied among species by 11.6° and *CT_min_* by 13°C, only slightly more. Variation in basal *CT_max_* that either exceeds or is similar to variation in basal *CT_min_* in springtails (see also Jensen *et al*., 2019 for among-population variation) is different to findings for many insects and for other terrestrial ectotherms generally, but not unlike the situation found for marine ectotherms (Sunday *et al*., 2011; Araújo *et al*., 2013). Clearly, some of this difference must reside in the reasons for the evolution of much higher *CT_max_* in springtail species that succeed when introduced outside their native range. One reason may be that such species tend also to experience regular disturbances, which might be associated with broader tolerance ranges (Coyle *et al*., 2017). Another may be that variation in *CT_max_* at the assemblage level is much greater than interspecific analyses tend to reveal (e.g. Kaspari *et al*., 2015; Kühsel and Blüthgen, 2015), which would have substantial implications for assessments of response to global climate change. Yet a third might be that the introduced species all come from regions, such as continental Europe, where thermal variation is much greater and much more predictably so than for the sub-Antarctic (Chown *et al*., 2004), resulting in greater physiological tolerance ranges than in the indigenous species. This latter hypothesis requires further investigation with information that enables the exact localities of origin of the introduced species to be identified—information that is slowly becoming available (e.g. Baird *et al*., 2020).

In the case of desiccation resistance, the variation found among species in the time to death at 76% humidity is largely consistent with findings from other species, often examined under less extreme desiccating conditions (e.g. Kærsgaard *et al*., 2004; Elnitsky *et al*., 2008; Sørensen and Holmstrup, 2011). That we found a positive effect of thermal acclimation at 20°C accords with the only previous investigation of such cross-tolerance effects for springtails (Chown *et al*., 2007). However, here, a similar effect for the indigenous *M. caeca* and no effects for the indigenous *P. insularis* and the alien *P. fimata*, differ from the outcomes of that work. There, acclimation at 5°C tended to improve performance of the indigenous species at that temperature, whereas acclimation to 15°C generally reduced it, with little difference among acclimation treatments at a 15°C test temperature. By contrast, acclimation to 15°C improved desiccation resistance at both the 5°C and 15°C test temperatures. Here, no such consistent differences among the indigenous and alien species were found. Basal desiccation resistance (measured as survival time) was on average, however, higher in the alien species, contrary to the previous work that found no such differences (Chown *et al*., 2007). Thus, differences in desiccation resistance among indigenous and alien springtail species cannot yet be generalized.

Intriguingly, despite the importance of water balance in determining the activity and distribution of ectotherms, and especially arthropods (Chown *et al*., 2011; Rozen-Rechels *et al*., 2019), and forecasts for substantial changes in water availability globally (Sarojini *et al*., 2016), few studies have focussed on determining the extent of differences among indigenous and alien species in traits related to water balance. In mosquitoes, desiccation tolerant eggs are associated with species that have become alien but not those that are invasive (Juliano and Lounibos, 2005). By contrast, in freshwater molluscs, the two groups of species do not differ in desiccation resistance (Collas *et al*., 2014).

**Table 8:** Performance curve statistics for egg development rate (1/days to hatching): slope of the linear part of the curve [estimate ± s.e. (n)], the slope given as electron volts from the equation ln rate vs. 1/kT (eV), the temperature of the fastest rate recorded (*T_opt_*), the development rate at that temperature (*U_max_*) [mean ± SD (*n*)] and the upper temperature where hatching success declined to 50% (HS ULT50) in springtails from Macquarie Island

**Species**	**Slope ± S.E. (*n*)**	**eV**	***T***_***opt***_	***U***_***max***_ **± SD**	**HS ULT50 ± S.E.**
**Alien**					
*C. denticulata*	0.00595 ± 0.00019 (5)	0.742	25	0.13266 ± 0.01027 (36)	24.5 ± 0.8
*H. purpurescens*	0.00266 ± 0.00026 (5)	0.744	20	0.05716 ± 0.00323 (31)	19.4 ± 0.5
*H. viatica*	0.00530 ± 0.00010 (3)	0.560	25	0.10304 ± 0.00783 (45)	26.3 ± 0.5
*Parisotoma notabilis*	0.00607 ± 0.00027 (3)	0.721	25	0.13516 ± 0.01454 (33)	25.0 ± 0.5
*P. fimata*	0.00445 ± 0.00027 (5)	0.826	25	0.09641 ± 0.00717 (42)	22.8 ± 0.7
*Proisotoma* sp.	0.00549 ± 0.00031 (4)	0.928	20	0.09441 ± 0.00578 (46)	23.5 ± 0.5
**Indigenous**					
*M. caeca*	0.00359 ± 0.00024 (4)	0.791	20	0.06440 ± 0.00717 (34)	19.4 ± 0.5
*P. insularis*	0.00468 ± 0.00003 (3)	0.997	15	0.06135 ± 0.00206 (44)	16.5 ± 0.4

Variation in development rate parameters has been the subject of two major studies contrasting indigenous and alien species. In the first (Jarošík *et al*., 2015), the sum of effective temperatures [1/slope of the rate-temperature relationship, SET)] and the lower development threshold (LDT) for insects across either part of the life cycle, or the full cycle, were compared among indigenous and invasive species. No significant differences were found for SET, but LDTs were lower for the invasive species (Jarošík *et al*., 2015). In the second study, of seven springtail species (Janion *et al*., 2010), the slopes of the rate-temperature relationships for egg development rate did not differ, although on average development rates were higher for the alien species, with lower hatching success at the higher temperatures for the indigenous species. Our results are largely consistent with these outcomes, so extending the findings for assemblage-level assessments. We found no significant differences between groups in the slope of the egg development-rate temperature relationship but a higher *T_opt_*, higher *U_max_* (when excluding *H. purpurescens*) and higher ULT50 in the indigenous compared with the invasive species. Although we did not calculate LDT (simply: -intercept/slope of the linear part of the rate-temperature relationship—see Jarošík *et al*., 2015), examination of the rate-temperature relationships and these values (Supplementary Table S8) suggests that no differences between the groups are to be expected. Thus, the egg development work here supports suggestions that the temperature sensitivity of development, a form of phenotypic plasticity (Ghalambor *et al*., 2007), does not differ between the two groups of species and in magnitude is in keeping with the variation previously found for arthropods (Irlich *et al*., 2009; Dell *et al*., 2011). Yet, it also shows that alien species typically have increased capacity to complete their development at higher temperatures and to do so at faster rates than their indigenous counterparts.

The insights presented here on assemblage level variation in traits among indigenous and alien species are necessary to clarify (i) how interactions among species will play out (Leibold and Chase, 2018) and (ii) how future changes to systems because of either local disturbances (such as urbanization, see e.g. Diamond *et al*., 2017), or global climate alterations (Sarojini *et al*., 2016), will differ from place to place and therefore assemblage to assemblage, so affecting indigenous-alien species interactions (Hulme, 2017). In this regard, two caveats apply to our study.

First, although we were able to investigate the most abundant species in the assemblage (see Terauds *et al*., 2011), we were not able to investigate the entire assemblage. At most, we included species representing 85% of the indigenous and >95% of the alien assemblage by abundance (Terauds *et al*., 2011). Nonetheless, the Macquarie Island assemblage now consists of 22 indigenous and 12 alien springtail species. That we found little difference between the F0 and F2 generations in critical thermal limits, and for most species also little difference among generations in desiccation resistance, suggests that examining recently captured individuals may not be as significant a concern as has been suggested (Hoffmann and Sgrò, 2017), especially when attempting to estimate the full suite of assemblage traits. The importance of investigating additional, and especially rare, species will depend on how important they are in the structure and functioning of the system in question, with evidence from other systems suggesting that rare species can be important and should be considered (Winfree *et al*., 2018; Dee *et al*., 2019).

Second, we did not differentiate between species that live above ground, in the litter, or deeper in the soil or among the major orders of springtails: the Symphypleona, Poduromorpha and Entomobryomorpha (Bellinger *et al*., 2019), as is often done (Janion *et al*., 2010; Bokhorst *et al*., 2012; Ellers *et al*., 2018). In part, we did not have sufficient species to undertake a full factorial design to enable us to do so, though the PGLS analyses mitigated these effects to some extent. We also think, however, that consideration at the assemblage level as a whole of how the distribution of individuals with different trait values might play out into the future is important at the local scale. Changes in response traits (Naeem and Wright, 2003) will take place through either differential survival or differential reproduction of individuals, altering the overall composition of the assemblage and its effects on ecosystem structure and functioning. Such whole-of-assemblage considerations of individuals from a trait perspective are becoming more common (e.g. Salo *et al*., 2020). Theory (e.g. Albert *et al*., 2012; Leibold and Chase, 2018) also suggests that they require further consideration when the outcome of the interactions between indigenous and alien species in a particular assemblage, under expected conditions of change, is being investigated.

Overall, our investigations have revealed that while basal trait values differ on average between the indigenous and alien species groups of Collembola, with the latter having the advantage under higher temperatures and drier conditions, phenotypic plasticity does not differ between them. These outcomes suggest that as local climates become warmer and, in some places, drier with global change, the conservation problems associated with biological invasions (McGeoch and Jetz, 2019) will increase, especially in soils (Coyle *et al*., 2017).

## Supplementary Material

suppl_data_coaa049
