# Supplementary material for: Basal tolerance but not plasticity gives invasive springtails the advantage in an assemblage setting
Source: Conserv Physiol. 2020 Jun 15;8(1):coaa049. doi: 10.1093/conphys/coaa049 (PMC7294889; doi:10.1093/conphys/coaa049)

**Basal tolerance but not plasticity gives invasive springtails the advantage in an assemblage setting**

**Laura M. Phillips^1^, Ian Aitkenhead^1^, Charlene Janion-Scheepers^2,3^, Catherine K. King^4^, Melodie A. McGeoch^1^, Uffe N. Nielsen^5^, Aleks Terauds^4^, W.P. Amy Liu^1^, Steven L. Chown^1^**

^1^School of Biological Sciences, Monash University, Victoria 3800, Australia

^2^Iziko South African Museum, Cape Town, 8001, South Africa

^3^Department of Biological Sciences, University of Cape Town, Rondebosch, Cape Town, 7700, South Africa

^4^Australian Antarctic Division, Department of Agriculture, Water and the Environment, 203 Channel Highway, Kingston, Tasmania 7050, Australia

^5^Hawkesbury Institute for the Environment, Western Sydney University, Locked Bag 1797, Penrith 2751, New South Wales, Australia

Corresponding author: School of Biological Sciences, Monash University, Victoria 3800, Australia. Tel: +61 3 99050097. Email: [steven.chown@monash.edu](mailto:steven.chown@monash.edu)

Lay summary

Whether invasive alien species will come to dominate animal assemblages as climates change, mediated by physiological trait differences, is a major conservation question. Here we show that basal physiological tolerances are higher for alien than indigenous assemblage members, but that phenotypic plasticity does not differ between them. A warmer, drier world will probably benefit alien species.

**As global climates change, alien species are anticipated to have a growing advantage relative to their indigenous counterparts, mediated through consistent trait differences between the groups. These insights have largely been developed based on interspecific comparisons using multiple species examined from different locations. Whether such consistent physiological trait differences are present within assemblages is not well understood, especially for animals. Yet it is at the assemblage level that interactions play out. Here we examine whether physiological trait differences observed at the interspecific level are also applicable to assemblages. We focus on the Collembola, an important component of the soil fauna characterised by invasions globally, and five traits related to fitness: critical thermal maximum, minimum and range, desiccation resistance, and egg development rate. We test the predictions that the alien component of a local assemblage has greater basal physiological tolerances or higher rates, and more pronounced phenotypic plasticity, than the indigenous component. Basal critical thermal maximum, thermal tolerance range, desiccation resistance, optimum temperature for egg development, the rate of development at that optimum, and the upper temperature limiting egg hatching success are all significantly higher, on average, for the alien than the indigenous components of the assemblage. Outcomes for critical thermal minimum are variable. No significant differences in phenotypic plasticity exist between the alien and indigenous components of the assemblage. These results are consistent with previous interspecific studies investigating basal thermal tolerance limits and development rates, and their phenotypic plasticity, in arthropods, but are inconsistent with results from previous work on desiccation resistance. Thus, for the Collembola, the anticipated advantage of alien over indigenous species under warming and drying is likely to be manifest in local assemblages, globally.**

**Key words:** Biological invasions, climate change, *CT_max_*, *CT_min_*, ectotherm, growth, water balance

**Introduction**

Although assemblages lie within a metacommunity setting, their dynamics are significantly mediated by the physical environments they encounter and by interactions among species. Because physiological traits modulate the effects of the environment on populations (Helmuth et al., 2005), knowing the range of trait variation for local assemblages, or significant components of them, can provide much insight into assemblage structure and dynamics (Albert et al., 2012; Leibold and Chase, 2018). The development of such understanding is especially important given the growing need to understand the mechanistic basis of a globally common pattern of high local turnover through time without large changes in the richness of assemblages (Blowes et al., 2019). Whether such dynamics into the future will include the rising dominance of assemblages by alien species, owing to the expectation that changing climates will generally benefit them (Hulme, 2017), is of much interest given the economic and conservation significance of biological invasions. Such invasions are among the most significant conservation concerns globally (McGeoch and Jetz, 2019).

Assessments of trait variation for significant proportions of the species in local assemblages are uncommon for animals (Chown and Gaston, 2016). Rather, the species compared are typically selected from different localities. Where assemblage investigations have been undertaken, the outcomes can be quite different to those involving interspecific comparisons (compare, for example, Diamond et al., 2012 with Kaspari et al., 2015). Hence the general macroecological insight that interspecific and assemblage-level investigations are different and may provide complementary or even contrary insights (Chown and Gaston, 2016).

For plants, explorations of the extent to which indigenous and alien species differ in their characteristics at the assemblage level are increasing (e.g., van Kleunen et al., 2018; Mathakutha et al., 2019; Sandel and Low, 2019). These comparisons explicitly test the ‘ideal weed’ and ‘plasticity’ hypotheses, proposing that invasion success of a non-native species depends on its specific traits, or enhanced phenotypic plasticity, respectively (Enders et al., 2020). By contrast, studies exploring whether the indigenous versus alien components of assemblages vary consistently in one or more physiological traits remain rare for animals. Most of the work on trait differences between indigenous and alien species is based on interspecific studies from animals collected across a wide range of localities (e.g., Moyle et al., 2013; Bradie and Leung, 2015; Jarošík et al., 2015; Allen et al., 2017; Janion-Scheepers et al., 2018), or for only a small component of a local assemblage (Stachowicz et al., 2002; Chown et al., 2007). These studies do not consider a range of species from a local setting. Yet they are frequently used as a basis to forecast rising success of alien species under changing climates (e.g., Janion-Scheepers et al., 2018). Indeed, because of the availability of the data, interspecific comparisons remain among the most common macrophysiological approaches adopted. Thus, insights into whether assemblages might be dominated by alien species as climates continue to change, and what mechanisms might lie at the heart thereof, may at best be incomplete, and at worst inaccurate. In consequence, much need exists to determine whether predictions made from interspecific studies are borne out at the assemblage level.

Here we therefore examine the extent to which empirical outcomes from interspecific studies of the trait differences among indigenous and invasive animal species, i.e. tests of the ideal weed and plasticity hypotheses (Enders et al., 2020), are borne out by a comprehensive investigation of a local assemblage. We use Collembola as a model group. Springtails are important in belowground systems and mediate aboveground ecological outcomes (Bardgett and van der Putten, 2014). Understanding of physiological trait diversity in the group is growing rapidly (Van Dooremalen et al., 2013; Ellers et al., 2018; Jensen et al., 2019). How this diversity might be partitioned among indigenous and invasive species has been the subject of recent attention at the interspecific level (Janion-Scheepers et al., 2018). Recent work has been spurred by concerns over the extent of soil invasions globally, including among Collembola, and by suggestions that anthropogenic change will exacerbate the impacts of invaders on soil systems (Cicconardi et al., 2017; Coyle et al., 2017; Geisen et al., 2019).

We consider five physiological traits that have significant influences on fitness and are therefore frequently incorporated into models of the likely impacts of environmental change on organisms. These are critical thermal minimum and maximum (and the derived trait of tolerance range), desiccation resistance, and egg development rate (Birkemoe and Leinaas, 2000; Kearney et al., 2013; Sinclair et al., 2016; Rozen-Rechels et al., 2019). The assemblage is that of sub-Antarctic Macquarie Island. We use this particular springtail assemblage because it is well surveyed both in terms of the species present and their abundances, is characterised by a range of alien species, and is representative with regard to Collembola invasions of several islands globally (Cicconardi et al., 2017; Baird et al., 2019). Moreover, because it is an island assemblage, local factors are likely to be more important in determining dynamics than regional biotic influences (Leibold and Chase, 2018). The general climate of Macquarie Island and its change over the past 40 years are also relatively well understood (Adams, 2009; Bergstrom et al., 2015).

Specifically, we test two predictions, based on general expectations (Daehler, 2003; Davidson et al., 2011; Hulme, 2017; Enders et al., 2020), and previous work on springtails (Chown et al., 2007; Janion et al., 2010; Janion-Scheepers et al., 2018). Compared with their indigenous counterparts, alien species should have (**Prediction 1**) greater basal physiological tolerances (for their definition see Chown and Nicolson, 2004) as suggested by the ideal weed hypothesis, and (**Prediction 2**) more pronounced phenotypic plasticity as suggested from the phenotypic plasticity hypothesis.

**Materials and methods**

Site description and species sampling

Collembola were collected from Macquarie Island (54°30' S, 158°57' E) in March/April of 2016 and 2017 (Supplementary Table S1). The island is small (12 800 ha), with a cool (mean air temperature range 3.8°C to 6.6°C), wet (~954 mm annual precipitation) and windy oceanic climate; vegetation varies from coastal tussock to higher elevation fellfield areas (Selkirk et al., 1990).

Springtail collection involved beating into a tray and aspirating individuals from a variety of vegetation types and from the soil surface into 70 ml plastic pots with a saturated Plaster-of-Paris and charcoal powder base (9:1 mixture) (hereafter lined plastic pots). Vegetation from the collection site was placed in to the pots as a food source. Initial sorting into additional lined plastic pots in the laboratory at Macquarie Island was undertaken to initially separate species and ensure densities of ~ 75-100 animals per pot. Food sources were also placed into these new pots. Turf samples (10 cm^2^ surface area, 5 cm deep) were also collected to ensure that springtails from all layers of soil were included. Springtails in pots and turf samples were maintained at ~5°C and on a 12:12 light:dark (L:D) cycle during the two-week transportation back to the laboratory in Melbourne. Here, springtails were extracted from turf samples into lined plastic pots over three weeks using Berlese-Tullgren funnels.

Springtails were identified using current available keys for Macquarie Island Collembola (e.g. Greenslade, 2006) (further verified with DNA barcoding (see Supplementary Table S2)) and sorted into species. DNA barcoding, involving the extraction and sequencing of 658 bp of the mitochondrial cytochrome oxidase subunit I gene (COI), was undertaken by the Canadian Centre for DNA Barcoding (CCDB, http://www.ccdb.ca/) at the Centre for Biodiversity Genomics, University of Guelph, Canada through the Barcode of Life Data Systems (BOLD, <http://www.boldsystems.org/>; Ratnasingham and Hebert, 2007) (see also Janion-Scheepers et al., 2018). A total of 91 individuals from 16 species was sequenced, with a minimum of three individuals for any one species (Supplementary Table S2). These sequences are available at the Barcode of Life Data Systems (BOLD) (www.boldsystems.org) under the larger Project ‘sub-Antarctic Collembola’. Species were classified as either indigenous to the island, or introduced by human activity (alien) based on previously published information (Greenslade, 2006; Phillips et al., 2017). Most of the alien species are widespread on the island and hence considered invasive (Terauds et al., 2011; Phillips et al., 2017).

Colony maintenance

Springtail colonies were maintained in a controlled temperature room at 10°C ([10.15 ± 0.23°C], verified with iButton Hygrochron® data loggers, Maxim Integrated, San Jose, USA) on a 12:12 L:D cycle. Individuals were maintained by species at intermediate density (75 - 100 individuals) in 70 ml lined plastic pots to maintain a humid environment (>99% relative humidity). They were fed weekly on a diet of *Platanus* sp. bark (Hoskins et al., 2015) to allow for self-selection of nutrients, with the bark also providing some shelter for individuals. Pots were randomly re-arranged in the controlled temperature room during feeding and during experiments to minimise shelf effects.

For experiments measuring thermal and desiccation resistance, springtails were assessed at the F0 and the F2 generations. F0 springtails were used to ensure that as much information on the assemblage could be captured as possible, including springtail species that we failed to rear successfully under laboratory conditions. The F2 generations of springtails were examined to ensure that carry-over effects from the environment of collection, including parental effects, were minimised, while also minimising adaptation to laboratory conditions (Hoffmann and Sgrò, 2017). For investigations of egg development rate, only the F2 generation was used. Eggs were removed weekly from parental pots (F0 individuals) and transferred to new pots to establish the F1 generation. The same process was then used to generate the F2 generation from F1 parents (following Janion-Scheepers et al., 2018). In each case, eggs from multiple adults were randomly combined within generations to maintain genetic diversity. F2 springtails reached adulthood between 5 and 16 months after field caught (F0) springtails entered the laboratory.

Critical thermal limits

Critical thermal limits provide a proxy for survival in active adult organisms (Lutterschmidt and Hutchinson, 1997), including in springtails (e.g., Everatt et al., 2013). The critical thermal maxima (*CT_max_*) and critical thermal minima (*CT_min_*) were determined for 16 species of springtails at F0 (9 alien, 7 indigenous, Supplementary Table S3), after they had been held at 10°C for one week, to examine differences in basal thermal tolerance between the indigenous and alien groups (**Prediction 1**). At the F2 generation, 10 species were investigated (7 alien, 3 indigenous, Supplementary Table S3). These F2 species were also examined for adult (short-term, non-developmental) plasticity in critical thermal limits (**Prediction 2**). Adult phenotypic plasticity was assessed by acclimating F2 springtails to one of five temperature treatments for seven days prior to experimentation (Supplementary Table S4). Three stable and two variable temperature acclimations were used. Much interest exists in understanding the extent to which fluctuating versus constant temperatures may alter estimates of phenotypic plasticity (Colinet et al., 2015). Recently, the importance of the influence of extreme temperature events on the evolution of thermal tolerance has been further emphasized, with the idea that extreme events disproportionately drive changes in such traits (Hoffmann, 2010; Kingsolver and Buckley, 2017). Stable temperatures were set at 5°C, 10°C and 15°C, and variable temperatures were set at 10°C with either a high (25°C) or a low (-5°C) extreme temperature spike that occurred for one hour each day, with a 30-minute temperature ramp up/down either side of the temperature extreme. The temperature spikes were based on extreme event temperatures from a long-term soil surface temperature record for the island (Leihy et al., 2018). Acclimation treatments were completed in controlled temperature cabinets (MIR-154-PE, Panasonic, Osaka, Japan) and rooms. Adults were held at the acclimation temperatures for one week (following Janion-Scheepers et al., 2018; Jensen et al., 2019).

Critical thermal limits were determined for individual adult springtails using established protocols (Janion-Scheepers et al., 2018). Springtails were contained within custom-built thermal stages (Monash University Instrument Facility, Clayton Campus, VIC, Australia) that were heated or cooled with programmable water baths (Grant Instruments, Cambridge model TXF200) at 0.05°C per minute. This rate was chosen for its environmental relevance, reflecting a commonly encountered rate of temperature change under microclimatic conditions (Allen et al., 2016). The floor of the stage was lined with saturated Plaster-of-Paris to minimise desiccation of springtails during experiments, and temperature of the stage floor was recorded using Omega thermometers (model: RDXL 12SD, Omega Engineering, Norwalk, USA) with type K thermocouples. A starting temperature of 10°C (rearing temperature) was used for all experiments. Springtails were observed every ~ 5°C until a behavioural change occurred (e.g., moving faster/slower) after which they were monitored every 1°C, and then every 0.5°C after the *CT_max_*/*CT_min_* was reached for the first individual in the experiment. *CT_max_* and *CT_min_* were defined as the temperature at which a loss of righting response occurred for each individual (Everatt et al., 2013; Janion-Scheepers et al., 2018). Different sets of individuals were used for the *CT_max_* and *CT_min_* experiments. Three replicates, typically of 10-15 individuals, were completed per species per treatment (sample sizes in Supplementary Table S3). Because variation in critical thermal limits may be affected by differences in body mass (Ribeiro et al., 2012), a mean body mass for each species was determined by weighing a random sample of 50 adult individuals of each species using a high-resolution (0.1 μg) microbalance (Mettler-Toledo XP2U, Switzerland) (Supplementary Table S5).

Desiccation resistance

Desiccation resistance was determined for 10 species of springtails at the F0 generation (5 alien, 5 indigenous, Supplementary Table S3) to examine differences in absolute desiccation resistance between alien and indigenous species (**Prediction 1**). In the F2 generation, 8 species were used (6 alien, 2 indigenous, Supplementary Table S3) to investigate plasticity in desiccation resistance in a cross-tolerance framework with temperature (**Prediction 2**). Short-term temperature acclimation has previously been shown to alter desiccation resistance in indigenous and alien springtails unequally, to the alien species’ advantage (Chown et al., 2007). Here, the effects of short-term temperature acclimations on desiccation resistance were examined at two acclimation and two test temperatures. F2 springtails were acclimated at either 10°C or 20°C in controlled temperature rooms for seven days prior to the desiccation experiment which was conducted at either 10°C or 20°C.

An experimental protocol for desiccation resistance, measured as survival time at 76% relative humidity, was established based on previous methods (Kærsgaard et al., 2004; Chown et al., 2007). Individual springtails were contained within glass vials covered with fine mesh, which were then housed in sealed, 70 mL plastic pots containing 15 mL of saturated NaCl solution as a desiccant. Saturated NaCl was used as it provides a consistent relative humidity of 76% from 0°C to 20°C. Furthermore, it has been shown that springtails can survive between one and 24 hours at this humidity (Chown et al., 2007). Each pot contained 2 glass vials with ~ 5 springtails per vial, and an iButton Hygrochron® data logger (Maxim Integrated, San Jose, USA) to verify temperature and relative humidity. Throughout the experiment, conducted in controlled-temperature rooms, springtails were examined every 10 minutes under a Leica M80 microscope (Leica Microsystems Pty Ltd, Wetzlar, Germany), and time to death (minutes) was recorded for each individual. Typically, four replicates of 10 individuals were used per experiment, with some exceptions for F0 experiments due to low numbers of springtails available (see Supplementary Table S3 for sample sizes). Following the experiment, springtails were dried at 40°C for 24 hours and then weighed in groups by replicate using a high-resolution (0.1 μg) microbalance (Mettler-Toledo XP2U, Switzerland) to obtain an estimate of individual dry body mass.

Egg development and hatching success

Egg development time and hatching success were determined for eight species, including six alien and two indigenous species (Supplementary Table S3), at seven temperatures ranging from 0°C – 30°C, in 5°C increments (Supplementary Table S4) (**Predictions** **1 and 2**) following previous protocols (Birkemoe and Leinaas, 2000; Janion et al., 2010). Eggs laid by F2 adults at 10°C were collected and transferred to each respective development temperature within 24 hours of laying. Eggs were transferred to 70 ml lined pots and kept in controlled temperature cabinets (MIR-154-PE, Panasonic) or rooms for the duration of development. Five replicate pots per temperature with 10 eggs per pot were used to provide a sample size of 50 eggs per temperature for each species. Eggs were checked daily for hatching. Days to hatching for each egg, and hatching success, measured as a percentage of eggs hatched within each pot, were recorded. Eggs were classified as unviable/dead if they were either visibly dead (shrivelled, dissolved, or extremely discoloured), or if they hadn’t hatched within 10 days (at 10°C – 30°C) or within 14 days of the previously hatched egg within the same pot (at 0°C and 5°C).

Statistical analyses

All analyses were undertaken using R v. 3.5.2 (R Core Team, 2018) implemented in R Studio v. 1.1.463. All code and data files are archived in the Monash Figshare repository (doi: 10.26180/5e17b874b125c and doi: 10.26180/5e17c3bc55197).

*Critical thermal limits*

To test **Prediction 1**, basal critical thermal limit differences (*CT_max_*, *CT_min_*, *CT_range_* [difference between species mean *CT_max_* and mean *CT_min_*]) among alien and indigenous species at the F0 generation (10°C acclimation) were assessed using two approaches. The first explored differences in *CT_max_* and *CT_min_* among individuals from the two groups excluding species identity, assuming that individual trait variation is important in a community dynamics context (Albert et al., 2012), using a linear model with status as a fixed factor. Because individuals were not weighed, mass was not included as a covariate. Differences between the alien and indigenous groups were then assessed using species means (or mean differences for *CT_range_*) within a phylogenetically explicit framework using Phylogenetic Generalised Least Squares (PGLS) (Garland and Ives, 2000), as implemented in the caper v. 0.5.2 package (Orme et al., 2013), including species mean mass as a covariate. Given small numbers of species, a Brownian Motion model of evolution was used (Cooper et al., 2016) and a maximum likelihood approach estimated Pagel’s λ (Pagel, 1999) to indicate the degree of phylogenetic influence in the data. The phylogenetic tree used for the analyses was based on Janion-Scheepers et al. (2018) and with mtCOI data used to infer species relationships. For the final tree, branch lengths were assigned using Grafen’s method (Grafen, 1989), and the tree (Supplementary Figure S1) is available as a Newick file in the Monash Figshare repository (doi: 10.26180/5e17b874b125c). Density plots made using the package ggplot2 were used to illustrate the range of variation in *CT_max_* and *CT_min_* for each species and across individuals in the full assemblage investigated.

Because differences in traits among F0 and F2 adults might arise for various reasons (Hoffmann and Sgrò, 2017), means of the critical thermal limit traits in the F0 and F2 generations, each acclimated for one week at 10°C, were compared among the ten species common to both sets of trials. A PGLS approach using a reduced tree was initially used. Because Pagel’s λ was estimated as zero for *CT_max_*, *CT_min_* and *CT_range_*, and because of the likely measurement variation of the traits, a ranged major axis model (RMA, Legendre and Legendre, 2012), implemented in the lmodel2 package, was used for each trait to determine whether the slope differed from 1 and the intercept from zero in each case by examining the 95% confidence intervals of the estimated values.

To test **Prediction 2**, the effects of acclimation to 5°C, 10°C and 15°C were examined for the F2 *CT_max_* and *CT_min_* trials by calculating an acclimation response ratio (ARR) (°C/°C) (Gunderson and Stillman, 2015). The ARR was calculated from the slope of the intraspecific relationship between acclimation temperature and critical thermal limits trait for each of the ten species investigated, based on individual data for each acclimation temperature. Systematic differences between the indigenous and alien species were investigated using a PGLS approach as above.

The impacts of a high (25°C) or low (-5°C) temperature spike for one hour on a daily basis as an extreme event acclimation treatment were compared for each of the ten species using linear models with temperature as a fixed factor and Tukey’s Honest Significant Difference (Crawley, 2013).

*Desiccation resistance*

Desiccation resistance was measured as individual survival time resulting in data that are bounded to the left at zero and right skewed (Supplementary Figure S2). To assess whether time to death differed between the F0 and F2 generations, five species for which F0 and F2 data were available at both acclimation and test temperatures of 10°C were each compared using a generalised linear model (GLM) assuming a quasipoisson distribution and a log link function because of the form of the data (O’Hara and Kotze, 2010). Because substantial differences between the F0 and F2 generations were found in one of the species, data from the F0 and F2 generations were not pooled for comparisons among indigenous and alien species, even though the two data series did not overlap completely in the available species.

To test **Prediction 1**, comparisons of the indigenous and alien species were made in two ways using the F0 data, in keeping with the previous approach. In the first, a GLM (assuming a quasipoisson distribution and a log link function) was used to compare the alien and indigenous assemblages (fixed factor), including an estimate of log_10_ dry body mass for each individual (from the individuals weighed at the end of the study) as a covariate. Thereafter, differences between the alien and indigenous groups were assessed using species means (here log_10_ of time to death to account for the skew in the data) using PGLS as described above, including species log_10_ mean dry mass as a covariate.

To test **Prediction 2**, the effects of thermal acclimation (fixed factor) on desiccation resistance were analysed for each species separately using a GLM assuming a quasipoisson distribution and a log link function. Acclimation at higher temperatures was expected to afford an extended survival time to the alien but not the indigenous species (Chown et al., 2007). Patterns of acclimation were compared visually for each of the groups, and then status (alien or indigenous) included in a model (as above) with all species.

*Egg development and hatching success*

Egg development times for individuals of each species at each temperature were converted to development rates (1/days to hatching). Because single individuals were not examined across a range of temperatures (different eggs were assessed at each temperature), a function-valued trait approach (Gomulkiewicz et al., 2018) was not implemented. Mean values for development rate were obtained for each species at each temperature and plotted against temperature.

Maximum development rate (*U_max_*) and the temperature at which this rate was realised (*T_opt_*) were extracted from the means data. **Prediction 1** was tested by inspecting the curves and selecting the appropriate mean values and temperatures, following previous approaches which have sought not to fit curves to the empirical data (Jarošík et al., 2015; Sørensen et al., 2018). Further to test **Prediction 1**, hatching success (as a proportion) was plotted against rearing temperature. This revealed that hatching success did not decline to zero at the lowest temperatures investigated in all of the species. Therefore, low temperature variation in hatching success was not investigated. Rather only the high temperature at which hatching success declined to 50% of the sample population, known as the Upper Lethal Temperature 50 (ULT50) was estimated using a generalized linear model assuming a binomial distribution and using a logit link function, with ULT50 values calculated from the fitted models using the Mass package (Crawley, 2013).

To test **Prediction 2**, for each species the slope of the relationship, or the temperature sensitivity of development, was calculated in two ways. First, a linear model was used to estimate the slope of the relationship between mean rate (1/days to hatching) at a given temperature and that temperature (°C) for each species. Data above the optimum temperature of the relationship (i.e. the temperature at maximum rate (see Sørensen et al., 2018)) were not used. Second, following a range of previous approaches (e.g., Dell et al., 2011), the natural logarithm of rate was plotted against 1/kT, where k = Boltzmann’s constant (8.617*10^-5^ ev.K^-1^) and T is temperature in Kelvin.

A PGLS approach, as described previously, was implemented to investigate differences between alien (six species) and indigenous groups (two species) in each of these four traits (slope, *U_max_*, *T_opt_*, ULT50). Here, Pagel’s λ was always estimated as zero. Because one of the alien species, *Hypogastrura purpurescens*, was found to be quite different to the others with regards to these variables, linear models used to assess differences between the alien and indigenous groups excluded this species.

**Results**

Critical thermal limits

The alien assemblage had, on average, a higher *CT_max_* and lower *CT_min_* than the indigenous assemblage (Table 1), although the alien assemblage was bimodal for *CT_max_* (Figure 1), largely owing to low values for *Proisotoma* sp. (Supplementary Figure S3). The PGLS models, based on species means (Table 2), revealed significant and substantial differences among the alien and indigenous species in *CT_max_* (4.1°C) and *CT_range_* (5.1°C), but not in *CT_min_*, with substantial phylogenetic signal in *CT_min_* only (Table 3). Species mean mass was not a significant covariate for any of the traits and was omitted in the final models.

The F0 and F2 generation data did not differ among the ten species as indicated by the slopes and intercepts of the RMA regressions not being different from 1 and 0, respectively, for *CT_max_* (Slope: 1.0, 95% C.I.s: 0.91 to 1.10; Intercept: 0.24, 95% C.I.s: -3.43 to 3.57), *CT_min_* (Slope: 0.87, 95% C.I.s: 0.51 to 1.47; Intercept: -0.65, 95% C.I.s: -2.04 to 1.65) and *CT_range_* (Slope: 0.98, 95% C.I.s: 0.82 to 1.17; Intercept: 1.21, 95% C.I.s: -6.12 to 7.40).

Acclimation Response Ratios (ARR in °C/°C) did not differ between the alien and indigenous groups for *CT_min_*, and only marginally so for *CT_max_* (Tables 1, 3). Thus, the ARR for these two traits is similar for the two groups of species, though the ARR for *CT_min_* (0.065 ± 0.038°C/°C) is significantly larger than the ARR for *CT_max_* (0.020 ± 0.031°C/°C) (linear model F_(1,18)_ = 8.29, p = 0.01) (summary data in Supplementary Table S6).

The extreme event treatments of either a low (-5°C) or high (25°C) temperature spike for one hour each day had limited and variable effects across the species, especially compared with either the constant 5°C acclimation in the former case, and the constant 15°C in the latter (Table 4; Supplementary Figures S4 and S5).

Desiccation resistance

In four of the five species for which data were available for both F0 and F2, no differences in time to death were found between the generations (GLM: *Ceratophysella denticulata* t = -0.788, p = 0.433; *Protaphorura fimata* t = 0.592, p = 0.556; *Mucrosomia caeca* t = -0.296, p = 0.768; *Parisotoma insularis* t = 0.363, p = 0.718), whereas in the fifth, *Proisotoma* sp., the F2 generation had a substantially and significantly longer time to death than the F0 generation (F0: 80 ± 32 minutes (median = 80), F2: 99 ± 28 minutes (median = 90); t = 2.99, p = 0.005). Thus, for the remainder of the investigations, the F0 and F2 generations were analysed separately.

For the F0 generation and using individual data, large and significant differences were found between the alien and indigenous species in time to death (Alien mean: 326 ± 413 minutes (median: 140); Indigenous mean: 115 ± 112 (median 60)), including with dry mass as a covariate (Table 5; Figure 2). The PGLS models using species means also showed significant and substantial differences among the alien and indigenous species in time to death, but with no phylogenetic signal in the data (Tables 3, 6).

Acclimation treatments in the F2 generation revealed that, as expected, time to death was shorter at the higher test temperatures, but that pre-exposure to an acclimation of 20°C frequently resulted in improved desiccation resistance either at 20°C (three species) or at 10°C (two species), although in two species no effects of acclimation were found (Figure 3; Table 7). Similar responses were found among the alien and in the indigenous species (e.g., in Figure 3 compare *Proisotoma* sp. (alien) with *M. caeca* (indigenous)), with the GLM supporting this interpretation given no interactions among status, acclimation and test temperatures (Supplementary Table S7).

Egg development and hatching success

Despite considerable differences in the form of the development rate – temperature curves among species (Figure 4), in the PGLS analyses only *T_opt_* differed between the alien and indigenous groups, with indigenous species having the lower value (Table 3). Excluding *H. pupurescens*, an outlier among the alien species (Table 8), resulted in rate-temperature slopes which still did not differ between the groups (slope: estimate (indigenous) = -0.0013 ± 0.0006, t = -2.34, p = 0.066; slope eV: estimate (indigenous) = 0.138 ± 0.116, t = 1.196, p = 0.285). Both *T_opt_* (estimate (indigenous) = -6.5 ± 2.1, t = -3.047, p = 0.029) and *U_max_* (estimate (indigenous) = -0.050 ± 0.015, t = -3.304, p = 0.021) were, however, lower in the indigenous than in the alien species group.

In all of the species, hatching success had declined to zero by 30°C, the highest temperature investigated. On average, the temperature at which hatching success had declined to 50% (ULT50) (Table 8), was significantly lower (by ~5.7°C) for the indigenous than for the alien species (Table 3), with *H. purpurescens* an outlier among the alien species.

**Discussion**

In this springtail assemblage from Macquarie Island, the outcomes of the tests of the two predictions are clear. **Prediction 1** (from the ideal weed hypothesis (Enders et al., 2020)), of greater basal physiological tolerance in the alien than in the indigenous species, is supported. On average, *CT_max_* is higher, *CT_range_* is broader, desiccation resistance is greater, and egg development *T_opt_* and *U_max_* and the ULT50 for egg hatching success, are higher in the alien than in the indigenous species. Only *CT_min_* is indistinguishable between these two groups at the species level. At the individual level, however, the difference in *CT_min_* between alien and indigenous species is clear. By contrast, **Prediction 2**, of greater phenotypic plasticity in the alien than in the indigenous species (from the phenotypic plasticity hypothesis (Enders et al., 2020)) is not supported. Acclimation responses for *CT_max_*, *CT_min_* and desiccation resistance, and the slopes of the rate-temperature relationships for egg development, do not differ between the alien and indigenous species groups.

Differences in basal tolerance, but not in phenotypic plasticity, of critical thermal limits specifically, are largely in keeping with previous work. The most extensive interspecific study to date (Janion-Scheepers et al., 2018) found that indigenous springtail species are characterised by critical thermal maxima which are on average 3°C lower than those of their alien counterparts, and a *CT_range_* difference of about the same magnitude, with no difference in *CT_min_* and acclimation response ratios between the groups. In this Macquarie Island assemblage, the differences in *CT_max_* and *CT_range_* are slightly larger (4°C and 5°C, respectively), but otherwise the findings accord closely. Moreover, irrespective of the acclimation conditions, we were unable to effect much change in the value of *CT_max_* in any of the species we investigated, reflected also by the low ARR values for this trait. Longer-term laboratory selection experiments have also been unable to do so in both alien and indigenous springtail species (Janion-Scheepers et al., 2018).

These findings of limited plasticity and adaptability in *CT_max_* over the shorter term are in keeping with previous investigations of other organisms (Hoffmann et al., 2013; Gunderson and Stillman, 2015; MacLean et al., 2019). Such similarity does not help to explain, however, why such substantial interspecific variation exists in the basal *CT_max_* of springtails. Here, for example, the largest difference in *CT_max_* among species is 8.6°C, whereas the largest difference in *CT_min_* is 5.1°C. In the broad-scale interspecific study (Janion-Scheepers et al., 2018), *CT_max_* varied among species by 11.6° and *CT_min_* by 13°C, only slightly more. Variation in basal *CT_max_* which either exceeds or is similar to variation in basal *CT_min_* in springtails (see also Jensen et al., 2019 for among-population variation) is different to findings for many insects and for other terrestrial ectotherms generally, but not unlike the situation found for marine ectotherms (Sunday et al., 2011; Araújo et al., 2013). Clearly, some of this difference must reside in the reasons for the evolution of much higher *CT_max_* in springtail species which succeed when introduced outside their native range. One reason may be that such species tend also to experience regular disturbances, which might be associated with broader tolerance ranges (Coyle et al., 2017). Another may be that variation in *CT_max_* at the assemblage level is much greater than interspecific analyses tend to reveal (e.g., Kaspari et al., 2015; Kühsel and Blüthgen, 2015), which would have substantial implications for assessments of response to global climate change. Yet a third might be that the introduced species all come from regions, such as continental Europe, where thermal variation is much greater and much more predictably so, than for the sub-Antarctic (Chown et al., 2004), resulting in greater physiological tolerance ranges than in the indigenous species. This latter hypothesis requires further investigation with information which enables the exact localities of origin of the introduced species to be identified – information that is slowly becoming available (e.g., Baird et al., 2020).

In the case of desiccation resistance, the variation found among species in the time to death at 76% humidity is largely consistent with findings from other species, often examined under less extreme desiccating conditions (e.g., Kærsgaard et al., 2004; Elnitsky et al., 2008; Sørensen and Holmstrup, 2011). That we found a positive effect of thermal acclimation at 20°C accords with the only previous investigation of such cross-tolerance effects for springtails (Chown et al., 2007). However, here, a similar effect for the indigenous *M. caeca* and no effects for the indigenous *P. insularis* and the alien *P. fimata*, differ from the outcomes of that work. There, acclimation at 5°C tended to improve performance of the indigenous species at that temperature, whereas acclimation to 15°C generally reduced it, with little difference among acclimation treatments at a 15°C test temperature. By contrast, acclimation to 15°C improved desiccation resistance at both the 5°C and 15°C test temperatures. Here, no such consistent differences among the indigenous and alien species were found. Basal desiccation resistance (measured as survival time) was on average, however, higher in the alien species, contrary to the previous work which found no such differences (Chown et al., 2007). Thus, differences in desiccation resistance among indigenous and alien springtail species cannot yet be generalised.

Intriguingly, despite the importance of water balance in determining the activity and distribution of ectotherms, and especially arthropods (Chown et al., 2011; Rozen-Rechels et al., 2019), and forecasts for substantial changes in water availability globally (Sarojini et al., 2016), few studies have focussed on determining the extent of differences among indigenous and alien species in traits related to water balance. In mosquitoes, desiccation tolerant eggs are associated with species that have become alien, but not those that are invasive (Juliano and Lounibos, 2005). By contrast, in freshwater molluscs, the two groups of species do not differ in desiccation resistance (Collas et al., 2014).

Variation in development rate parameters has been the subject of two major studies contrasting indigenous and alien species. In the first (Jarošík et al., 2015), the sum of effective temperatures (1/slope of the rate-temperature relationship, SET)) and the lower development threshold (LDT) for insects across either part of the life cycle, or the full cycle, were compared among indigenous and invasive species. No significant differences were found for SET, but LDTs were lower for the invasive species (Jarošík et al., 2015). In the second study, of seven springtail species (Janion et al., 2010), the slopes of the rate-temperature relationships for egg development rate did not differ, although on average development rates were higher for the alien species, with lower hatching success at the higher temperatures for the indigenous species. Our results are largely consistent with these outcomes, so extending the findings for assemblage-level assessments. We found no significant differences between groups in the slope of the egg development-rate temperature relationship, but a higher *T_opt_*, higher *U_max_* (when excluding *H. purpurescens*) and higher ULT50 in the indigenous compared with the invasive species. Although we did not calculate LDT (simply: -intercept/slope of the linear part of the rate-temperature relationship – see Jarošík et al., 2015), examination of the rate-temperature relationships and these values (Supplementary Table S8) suggests that no differences between the groups are to be expected. Thus, the egg development work here supports suggestions that the temperature sensitivity of development, a form of phenotypic plasticity (Ghalambor et al., 2007), does not differ between the two groups of species, and in magnitude is in keeping with the variation previously found for arthropods (Irlich et al., 2009; Dell et al., 2011). Yet it also shows that alien species typically have increased capacity to complete their development at higher temperatures, and to do so at faster rates, than their indigenous counterparts.

The insights presented here on assemblage level variation in traits among indigenous and alien species are necessary to clarify i) how interactions among species will play out (Leibold and Chase, 2018), and ii) how future changes to systems, because of either local disturbances (such as urbanisation, see e.g., Diamond et al., 2017), or global climate alterations (Sarojini et al., 2016), will differ from place to place, and therefore assemblage to assemblage, so affecting indigenous-alien species interactions (Hulme, 2017). In this regard, two caveats apply to our study.

First, although we were able to investigate the most abundant species in the assemblage (see Terauds et al., 2011), we were not able to investigate the entire assemblage. At most, we included species representing 85% of the indigenous and >95% of the alien assemblage, by abundance (Terauds et al., 2011). Nonetheless, the Macquarie Island assemblage now consists of 22 indigenous and 12 alien springtail species. That we found little difference between the F0 and F2 generations in critical thermal limits, and for most species also little difference among generations in desiccation resistance, suggests that examining recently captured individuals may not be as significant a concern as has been suggested (Hoffmann and Sgrò, 2017), especially when attempting to estimate the full suite of assemblage traits. The importance of investigating additional, and especially rare, species will depend on how important they are in the structure and functioning of the system in question, with evidence from other systems suggesting that rare species can be important and should be considered (Winfree et al., 2018; Dee et al., 2019).

Second, we did not differentiate between species that live above ground, in the litter, or deeper in the soil, or among the major orders of springtails: the Symphypleona, Poduromorpha and Entomobryomorpha (Bellinger et al., 2019), as is often done (Janion et al., 2010; Bokhorst et al., 2012; Ellers et al., 2018). In part, we did not have sufficient species to undertake a full factorial design to enable us to do so, though the PGLS analyses mitigated these effects to some extent. We also think, however, that consideration at the assemblage level as a whole of how the distribution of individuals with different trait values might play out into the future is important at the local scale. Changes in response traits (Naeem and Wright, 2003) will take place through either differential survival or differential reproduction of individuals, altering the overall composition of the assemblage and its effects on ecosystem structure and functioning. Such whole-of-assemblage considerations of individuals from a trait perspective are becoming more common (e.g., Salo et al., 2020). Theory (e.g., Albert et al., 2012; Chase and Leibhold, 2018) also suggests that they require further consideration when the outcome of the interactions between indigenous and alien species in a particular assemblage, under expected conditions of change, are being investigated.

Overall, our investigations have revealed that while basal trait values differ on average between the indigenous and alien species groups of Collembola, with the latter having the advantage under higher temperatures and drier conditions, phenotypic plasticity does not differ between them. These outcomes suggest that as local climates become warmer and, in some places, drier with global change, the conservation problems associated with biological invasions (McGeoch and Jetz, 2019) will increase, especially in soils (Coyle et al., 2017).

**Supplementary material**

Supplementary material is available at Conservation Physiology online.

**Acknowledgments**

Rebecca Hallas manages the laboratory in which most of this work was done. The staff of the Australian Antarctic Division provided support in the field at Macquarie Island, and in Australia. Jodie Weller and Gwen Fenton helped ensure the success of this project in the face of a major setback. Collections at Macquarie Island were made with the permission of the Tasmanian Parks and Wildlife Service under collection permits FA15328, FA17039, FL16017. We thank M. Potapov, W. Weiner and L. Deharveng for assistance with species identifications, and three anonymous reviewers for their helpful comments.

**Funding**

This work was supported by Australian Antarctic Science Grant [4307] and by Australian Research Council Discovery Project [DP190100341].

**References**

Adams N (2009) Climate trends at Macquarie Island and expectations of future change in the sub-Antarctic. *Pap Proc R Soc Tasmania* 143: 1-8.

Albert CH, de Bello F, Boulangeat I, Pellet G, Lavorel S, Thuiller W (2012) On the importance of intraspecific variability for the quantification of functional diversity. *Oikos* 121: 116-126.

Allen JL, Chown SL, Janion-Scheepers C, Clusella-Trullas S (2016) Interactions between rates of temperature change and acclimation affect latitudinal patterns of warming tolerance. *Conserv Physiol* 4: cow053.

Allen WL, Street SE, Capellini I (2017) Fast life history traits promote invasion success in amphibians and reptiles. *Ecol Lett* 20: 222-230.

Araújo MB, Ferri-Yáñez F, Bozinovic F, Marquet PA, Valladares F, Chown SL (2013) Heat freezes niche evolution. *Ecol Lett* 16: 1206-1219.

Baird HP, Janion‐Scheepers C, Stevens MI, Leihy RI, Chown SL (2019) The ecological biogeography of indigenous and introduced Antarctic springtails. *J Biogeogr* 46: 1959-1973.

Baird HP, Moon KL, Janion-Scheepers C, Chown SL (2020) Springtail phylogeography highlights biosecurity risks of repeated invasions and intraregional transfers among remote islands. *Evol Appl* doi: 10.1111/eva.12913.

Bardgett RD, van der Putten WH (2014) Belowground biodiversity and ecosystem functioning. *Nature* 515: 505-511.

Bellinger PF, Christiansen KA, Janssens F (2019) Checklist of the Collembola of the World. http://www.collembola.org

Bergstrom DM, Bricher PK, Raymond B, Terauds A, Doley D, McGeoch MA, Whinam J, Glen M, Yuan Z, Kiefer K *et al.* (2015) Rapid collapse of a sub-Antarctic alpine ecosystem: the role of climate and pathogens. *J Appl Ecol* 52: 774-783.

Birkemoe T, Leinaas HP (2000) Effects of temperature on the development of an Arctic Collembola (*Hypogastrura tullbergi*). *Funct Ecol* 14: 693-700.

Blowes SA, Supp SR, Antão LH, Bates AE, Bruelheide H, Chase JM, Moyes F, Magurran AE, McGill BJ, Myers-Smith IH, Winter M, Bjorkman AD, Bowler DE, Byrnes JEK, Gonzalez A, Hines JE, Isbell F, Jones HP, Navarro LM, Thompson PL, Vellend M, Waldock C, Dornelas M (2019) The geography of biodiversity change in marine and terrestrial assemblages. *Science* 366: 339-345.

Bokhorst S, Phoenix GK, Bjerke JW, Callaghan TV, Huyer-Brugman F, Berg MP (2012) Extreme winter warming events more negatively impact small rather than large soil fauna: shift in community composition explained by traits not taxa. *Global Change Biol* 18: 1152-1162.

Bradie J, Leung B (2015) Pathway-level models to predict non-indigenous species establishment using propagule pressure, environmental tolerance and trait data. *J Appl Ecol* 52: 100-109.

Chown SL, Gaston KJ (2016) Macrophysiology - progress and prospects. *Funct Ecol* 30: 330-344.

Chown SL, Slabber S, McGeoch MA, Janion C, Leinaas HP (2007) Phenotypic plasticity mediates climate change responses among invasive and indigenous arthropods. *Proc R Soc B* 274: 2661-2667.

Chown SL, Nicolson SW (2004) Insect Physiological Ecology. Mechanisms and Patterns. Oxford University Press, Oxford.

Chown SL, Sinclair BJ, Leinaas HP, Gaston KJ (2004) Hemispheric asymmetries in biodiversity - a serious matter for ecology. *PLoS Biol* 2: 1701-1707.

Chown SL, Sørensen JG, Terblanche JS (2011) Water loss in insects: an environmental change perspective. *J Insect Physiol* 57: 1070-1084.

Cicconardi F, Borges PAV, Strasberg D, Oromi P, Lopez H, Perez-Delgado AJ, Casquet J, Caujape-Castells J, Fernandez-Palacios JM, Thebaud C *et al.* (2017) MtDNA metagenomics reveals large-scale invasion of belowground arthropod communities by introduced species. *Mol Ecol* 26: 3104-3115.

Colinet H, Sinclair BJ, Vernon P, Renault D (2015) Insects in fluctuating thermal environments. *Annu Rev Entomol* 60: 123-140.

Collas FPL, Koopman KR, Hendriks AJ, van der Velde G, Verbrugge LNH, Leuven RSEW (2014) Effects of desiccation on native and non-native molluscs in rivers. *Freshw Biol* 59: 41-55.

Cooper N, Thomas GH, Venditti C, Meade A, Freckleton RP (2016) A cautionary note on the use of Ornstein Uhlenbeck models in macroevolutionary studies. *Biol J Linn Soc* 118: 64-77.

Coyle DR, Nagendra UJ, Taylor MK, Campbell JH, Cunard CE, Joslin AH, Mundepi A, Phillips CA, Callaham MA (2017) Soil fauna responses to natural disturbances, invasive species, and global climate change: current state of the science and a call to action. *Soil Biol Biochem* 110: 116-133.

Crawley MJ (2013) The R Book. John Wiley & Sons, Chichester.

Daehler CC (2003) Performance comparisons of co-occurring native and alien invasive plants: implications for conservation and restoration. *Annu Rev Ecol Evol Syst* 34: 183-211.

Davidson AM, Jennions M, Nicotra AB (2011) Do invasive species show higher phenotypic plasticity than native species and, if so, is it adaptive? A meta-analysis. *Ecol Lett* 14: 419-431.

Dee LE, Cowles J, Isbell F, Pau S, Gaines SD, Reich PB (2019) When do ecosystem services depend on rare species? *Trends in Ecology and Evolution* 34: 746-758.

Dell AI, Pawar S, Savage VM (2011) Systematic variation in the temperature dependence of physiological and ecological traits. *Proc Natnl Acad Sci USA* 108: 10591-10596.

Diamond SE, Chick L, Perez A, Strickler SE, Martin RA (2017) Rapid evolution of ant thermal tolerance across an urban-rural temperature cline. *Biol J Linn Soc* 121: 248-257.

Diamond SE, Sorger DM, Hulcr J, Pelini SL, Toro ID, Hirsch C, Oberg E, Dunn RR (2012) Who likes it hot? A global analysis of the climatic, ecological, and evolutionary determinants of warming tolerance in ants. *Global Change Biol* 18: 448-456.

Ellers J, Berg MP, Dias ATC, Fontana S, Ooms A, Moretti M (2018) Diversity in form and function: vertical distribution of soil fauna mediates multidimensional trait variation. *J Animal Ecol* 87: 933-944.

Elnitsky MA, Benoit JB, Denlinger DL, Lee RE (2008) Desiccation tolerance and drought acclimation in the Antarctic collembolan *Cryptopygus antarcticus*. *J Insect Physiol* 54: 1432-1439.

Enders M, Havemann F, Ruland F, Bernard-Verdier M, Catford JA, Gómez-Aparicio L, Haider S, Heger T, Kueffer C, Kühn I, Meyerson LA, Musseau C, Novoa A, Ricciardi A, Sagouis A, Schittko C, Strayer DL, Vilà M, Essl F, Hulme PE, van Kleunen M, Kumschick S, Lockwood JL, Mabey AL, McGeoch MA, Palma E, Pyšek P, Saul W-C, Yannelli FA, Jeschke JM (2020) A conceptual map of invasion biology: integrating hypotheses into a consensus network. *Global Ecol Biogeogr* doi: 10.1111/geb.13082.

Everatt MJ, Bale JS, Convey P, Worland MR, Hayward SA (2013) The effect of acclimation temperature on thermal activity thresholds in polar terrestrial invertebrates. *J Insect Physiol* 59: 1057-1064.

Garland T, Ives AR (2000) Using the past to predict the present: confidence intervals for regression equations in phylogenetic comparative methods. *Am Nat* 155: 346-364.

Geisen S, Wall DH, van der Putten WH (2019) Challenges and opportunities for soil biodiversity in the Anthropocene. *Curr Biol* 29: R1036-R1044.

Ghalambor CK, McKay JK, Carroll SP, Reznick DN (2007) Adaptive versus non-adaptive phenotypic plasticity and the potential for contemporary adaptation in new environments. *Funct Ecol* 21: 394-407.

Gomulkiewicz R, Kingsolver JG, Carter PA, Heckman N (2018) Variation and evolution of function-valued traits. *Annu Rev Ecol Evol Syst* 49: 139-164.

Grafen A (1989). The phylogenetic regression. *Phil Trans R Soc B* 326: 119-157.

Greenslade P (2006) The Invertebrates of Macquarie Island. Australian Antarctic Division, Kingston, Australia.

Gunderson AR, Stillman JH (2015) Plasticity in thermal tolerance has limited potential to buffer ectotherms from global warming. *Proc R Soc B* 282: 20150401.

Helmuth B, Kingsolver JG, Carrington E (2005) Biophysics, physiological ecology, and climate change: does mechanism matter? *Annu Rev Physiol* 67: 177-201.

Hoffmann AA (2010) Physiological climatic limits in *Drosophila*: patterns and implications. *J Exp Biol* 213: 870-880.

Hoffmann AA, Chown SL, Clusella-Trullas S (2013) Upper thermal limits in terrestrial ectotherms: how constrained are they? *Funct Ecol* 27: 934-949.

Hoffmann AA, Sgrò CM (2017) Comparative studies of critical physiological limits and vulnerability to environmental extremes in small ectotherms: how much environmental control is needed? *Integr Zool* 13: 355-371.

Hoskins JL, Janion-Scheepers C, Chown SL, Duffy GA (2015) Growth and reproduction of laboratory-reared neanurid Collembola using a novel slime mould diet. *Sci Rep* 5: 11957.

Hulme PE (2017) Climate change and biological invasions: evidence, expectations, and response options. *Biol Rev* 92: 1297-1313.

Irlich UM, Terblanche JS, Blackburn TM, Chown SL (2009) Insect rate-temperature relationships: environmental variation and the metabolic theory of ecology. *Am Nat* 174: 819-835.

Janion C, Leinaas HP, Terblanche JS, Chown SL (2010) Trait means and reaction norms: the consequences of climate change/invasion interactions at the organism level. *Evol Ecol* 24: 1365-1380.

Janion-Scheepers C, Phillips L, Sgro CM, Duffy GA, Hallas R, Chown SL (2018) Basal resistance enhances warming tolerance of alien over indigenous species across latitude. *Proc Natnl Acad Sci USA* 115: 145-150.

Jarošík V, Kenis M, Honěk A, Skuhrovec J, Pyšek P (2015) Invasive insects differ from non-invasive in their thermal requirements. *PLoS ONE* 10: e0131072.

Jensen A, Alemu T, Alemneh T, Pertoldi C, Bahrndorff S (2019) Thermal acclimation and adaptation across populations in a broadly distributed soil arthropod. *Funct Ecol* 33: 833-845.

Juliano SA, Lounibos PL (2005) Ecology of invasive mosquitoes: effects on resident species and on human health. *Ecol Lett* 8: 558-574

Kærsgaard CW, Holmstrup M, Malte H, Bayley M (2004) The importance of cuticular permeability, osmolyte production and body size for the desiccation resistance of nine species of Collembola. *J Insect Physiol* 50: 5-15.

Kaspari M, Clay NA, Lucas J, Yanoviak SP, Kay A (2015) Thermal adaptation generates a diversity of thermal limits in a rainforest ant community. *Global Chang Biol* 21: 1092-1102.

Kearney MR, Simpson SJ, Raubenheimer D, Kooijman SALM (2013) Balancing heat, water and nutrients under environmental change: a thermodynamic niche framework. *Funct Ecol* 27: 950–966.

Kingsolver JG, Buckley LB (2017) Quantifying thermal extremes and biological variation to predict evolutionary responses to changing climate. *Phil Trans R Soc B* 372: 20160147.

Kühsel S, Blüthgen N (2015) High diversity stabilizes the thermal resilience of pollinator communities in intensively managed grasslands. *Nature Comms* 6: 7989.

Legendre P, Legendre L (2012) Numerical Ecology. Elsevier, Amsterdam.

Leibold MA, Chase, JM (2018) Metacommunity Ecology. Princeton University Press, Princeton

Leihy RI, Duffy GA, Nortje E, Chown SL (2018) High resolution temperature data for ecological research and management on the Southern Ocean islands. *Sci Data* 5: 180177.

Lutterschmidt WI, Hutchison VH (1997) The critical thermal maximum: history and critique. *Can J Zool* 75: 1561-1574.

MacLean HJ, Sorensen JG, Kristensen TN, Loeschcke V, Beedholm K, Kellermann V, Overgaard J (2019) Evolution and plasticity of thermal performance: an analysis of variation in thermal tolerance and fitness in 22 *Drosophila* species. *Phil Trans R Soc B* 374: 20180548.

Mathakutha R, Steyn C, le Roux PC, Blom IJ, Chown SL, Daru BH, Ripley BS, Louw A, Greve M (2019) Invasive species differ in key functional traits from native and non‐invasive alien plant species. *J Vegetat Sci* 30: 994-1006.

McGeoch MA, Jetz W (2019) Measure and reduce the harm caused by biological invasions. *One Earth* 1: 171-174.

Moyle PB, Kiernan JD, Crain PK, Quinones RM (2013) Climate change vulnerability of native and alien freshwater fishes of California: a systematic assessment approach. *PLoS ONE* 8: e63883.

Naeem S, Wright JP (2003) Disentangling biodiversity effects on ecosystem functioning: Deriving solutions to a seemingly insurmountable problem. *Ecol Lett* 6: 567-579.

O’Hara RB, Kotze DJ (2010) Do not log-transform count data. *Methods Ecol Evol* 1: 118-122.

Orme D, Freckleton RP, Thomas GH, Petzoldt T, Fritz SA, Isaac NJB (2013) CAPER: comparative analyses of phylogenetics and evolution in R. *Methods Ecol Evol* 3: 145-151

Pagel M (1999). Inferring the historical patterns of biological evolution. *Nature* 401: 877-884.

Phillips L, Janion-Scheepers C, Houghton M, Terauds A, Potapov M, Chown SL (2017) Range expansion of two invasive springtails on sub-Antarctic Macquarie Island. *Polar Biol* 40: 2137-2142.

R Core Team (2018) R: A language and environment for statistical computing. R Foundation for Statistical Computing, Vienna, Austria. Available at https://www.r-project.org/.

Ratnashingham S, Hebert PDN (2007) BOLD: The Barcode of Life Data System (www.barcodinglife.org). *Mol Ecol Notes* 7: 355-364.

Ribeiro PL, Camacho A, Navas CA (2012) Considerations for assessing maximum critical temperatures in small ectothermic animals: insights from leaf-cutting ants. *PLoS ONE* 7: e32083.

Rozen-Rechels D, Dupoué A, Lourdais O, Chamaillé-Jammes S, Meylan S, Clobert J, Le Galliard JF (2019) When water interacts with temperature: ecological and evolutionary implications of thermo-hydroregulation in terrestrial ectotherms. *Ecol Evol* 9: 10029-10043.

Salo T, Mattila J, Eklöf J (2020) Long‐term warming affects ecosystem functioning through species turnover and intraspecific trait variation. *Oikos* 129: 283-295.

Sandel B, Low R (2019) Intraspecific trait variation, functional turnover and trait differences among native and exotic grasses along a precipitation gradient. *J Vegetat* Sci 30: 633-643.

Sarojini BB, Stott PA, Black E (2016) Detection and attribution of human influence on regional precipitation. *Nature Clim Change* 6: 669-675.

Selkirk PM, Seppelt RD, Selkirk DR (1990) Subantarctic Macquarie Island: Environment and Biology. Cambridge University Press, Cambridge.

Sinclair BJ, Marshall KE, Sewell MA, Levesque DL, Willett CS, Slotsbo S, Dong Y, Harley CD, Marshall DJ, Helmuth BS *et al.* (2016) Can we predict ectotherm responses to climate change using thermal performance curves and body temperatures? *Ecol Lett* 19: 1372-1385.

Sørensen JG, Holmstrup M (2011) Cryoprotective dehydration is widespread in Arctic springtails. *J Insect Physiol* 57: 1147-1153.

Sørensen JG, White CR, Duffy GA, Chown SL (2018) A widespread thermodynamic effect, but maintenance of biological rates through space across life's major domains. *Proc R Soc B* 285: 20181775.

Stachowicz JJ, Terwin JR, Whitlatch RB, Osman RW (2002) Linking climate change and biological invasions: ocean warming facilitates nonindigenous species invasions. *Proc Natnl Acad Sci USA* 99: 15497-15500.

Sunday JM, Bates AE, Dulvy NK (2011) Global analysis of thermal tolerance and latitude in ectotherms. *Proc R Soc B* 278: 1823-1830.

Terauds A, Chown SL, Bergstrom DM (2011) Spatial scale and species identity influence the indigenous–alien diversity relationship in springtails. *Ecology* 92: 1436-1447.

van Dooremalen C, Berg MP, Ellers J (2013) Acclimation responses to temperature vary with vertical stratification: implications for vulnerability of soil-dwelling species to extreme temperature events. *Global Change Biol* 19: 975-984.

van Kleunen M, Bossdorf O, Dawson W (2018) The ecology and evolution of alien plants. *Annu Rev Ecol Evol Syst* 49: 25-47.

Winfree R, Reilly JR, Bartomeus I, Cariveau DP, Williams NM, Gibbs J (2018) Species turnover promotes the importance of bee diversity for crop pollination at regional scales. *Science* 359: 791-793.

**Table 1:** Means and standard deviations for critical thermal limits at the assemblage level, including linear model comparison outcomes, and species-level means, standard deviations and ranges for acclimation response ratios (ARR).

| **Assemblage level critical thermal limits** | | | | | |
| --- | --- | --- | --- | --- | --- |
|  | **Indigenous** | **Alien** | **Linear model outcomes** | | |
|  | Mean ± s.d. | Mean ± s.d. | F | df | p |
| *CT_max_* (°C) | 31.9 ± 2.0 | 36.1 ± 2.6 | 397.3 | 1, 512 | < 0.0001 |
| *CT_min_* (°C) | -2.8 ± 1.0 | -3.9 ± 1.6 | 86.7 | 1, 528 | < 0.0001 |
|  |  |  |  |  |  |
| **Species critical thermal limit acclimation response ratios** | | | | | |
|  | **Indigenous** | **Alien** |  |  |  |
|  | **Mean ± s.d.**  **(range)** | **Mean ± s.d.**  **(range)** |  |  |  |
| ARR *CT_max_* (°C/°C) | 0.049 ± 0.041 | 0.001 ± 0.017 |  |  |  |
|  | (0.014 – 0.095) | (-0.018 – 0.027) |  |  |  |
|  |  |  |  |  |  |
| ARR *CT_min_* (°C/°C) | 0.070 ± 0.045 | 0.062 ± 0.037 |  |  |  |
|  | (0.025 – 0.120) | (0.011 – 0.117) |  |  |  |

**Table 2:** Critical thermal limits for springtail species from Macquarie Island.

| **Species** | **n** | ***CT_max_* ± S.D.** | **n** | ***CT_min_* ± S.D.** | **CT*_range_*** |
| --- | --- | --- | --- | --- | --- |
| **Alien** |  |  |  |  |  |
| *Ceratophysella denticulata* | 31 | 37.8 ± 0.4 | 30 | -4.4 ± 0.7 | 42.3 |
| *Desoria tigrina* | 34 | 32.0 ± 0.5 | 35 | -2.8 ± 0.6 | 34.8 |
| *Hypogastrura purpurescens* | 31 | 36.1 ± 0.3 | 31 | -4.5 ± 0.7 | 40.6 |
| *Hypogastrura viatica* | 42 | 38.3 ± 0.5 | 51 | -5.7 ± 1.2 | 44.0 |
| *Lepidocyrtus* sp*.* nr. *violaceus* | 30 | 37.8 ± 0.7 | 31 | -3.6 ± 0.9 | 41.4 |
| *Megalothorax* nr. *minimus* | 29 | 31.0 ± 1.4 | 28 | -0.6 ± 1.0 | 31.6 |
| *Parisotoma notabilis* | 31 | 36.2 ± 0.4 | 30 | -3.7 ± 0.5 | 39.9 |
| *Protaphorura fimata* | 33 | 38.0 ± 0.5 | 33 | -4.5 ± 0.5 | 42.5 |
| *Proisotoma* sp. | 32 | 37.0 ± 0.7 | 40 | -3.8 ± 0.8 | 40.8 |
|  |  |  |  |  |  |
| **Indigenous** |  |  |  |  |  |
| *Folsomotoma punctata* | 35 | 30.5 ± 1.2 | 35 | -2.5 ± 0.4 | 33.0 |
| *Katianna banzarei* | 32 | 29.7 ± 0.6 | 29 | -1.6 ± 0.5 | 31.3 |
| *Lepidobrya mawsoni* | 28 | 31.4 ± 1.1 | 33 | -2.1 ± 0.8 | 33.5 |
| *Mucrosomia caeca* | 31 | 33.8 ± 0.8 | 30 | -3.2 ± 0.6 | 37.0 |
| *Parisotoma insularis* | 33 | 31.3 ± 0.8 | 34 | -3.6 ± 0.6 | 34.9 |
| *Sminthurinus* cf. *tuberculatus* | 31 | 35.3 ± 1.0 | 30 | -3.7 ± 0.9 | 38.9 |
| *Tullbergia bisetosa* | 31 | 31.4 ± 1.1 | 30 | -2.7 ± 1.2 | 34.1 |

*CT_max_*: critical thermal maximum; *CT_min_*: critical thermal minimum; *CT_range_*: mean *CT_max_* minus mean *CT_min_*; S.D.: standard deviation.

**Table 3:** Outcomes of the Phylogenetic Generalised Least Squares analyses for assessment of differences between the indigenous and alien species groups for the traits investigated in this study. In each case the difference between the alien and indigenous species groups are shown (status (indigenous)), and the full model statistics provided including a maximum likelihood estimate of Pagel’s λ (_ML_λ). ARR = acclimation response ratio.

| **CT values** | **Estimate ± S.E.** | **t** | **P** |
| --- | --- | --- | --- |
| ***Thermal tolerance*** |  |  |  |
| ***CT_max_*** |  |  |  |
| Intercept | 36.02 ± 0.80 | 44.92 | < 0.0001 |
| Status (indigenous) | -4.11 ± 1.21 | -3.39 | 0.0045 |
|  | F_(1,14)_ = 11.47, R^2^ = 0.41, _ML_λ = 0.00 | |  |
| ***CT_min_*** |  |  |  |
| Intercept | -3.42 ± 0.71 | -4.60 | 0.0004 |
| Status (indigenous) | 0.84 ± 0.45 | 1.87 | 0.083 |
|  | F_(1,14)_ = 3.49, R^2^ = 0.14, _ML_λ = 0.75 | |  |
| ***CT_range_*** |  |  |  |
| Intercept | 39.76 ± 1.15 | 34.47 | < 0.0001 |
| Status (indigenous) | -5.07 ± 1.74 | -2.91 | 0.011 |
|  | F_(1,14)_ = 8.47, R^2^ = 0.33, _ML_λ = 0.00 | |  |
| **ARR *CT_max_*** |  |  |  |
| Intercept | 0.0076 ± 0.0097 | 0.784 | 0.456 |
| Status (indigenous) | 0.0410 ± 0.0176 | 2.323 | 0.049 |
|  | F_(1,8)_ = 5.40, R^2^ = 0.33, _ML_λ = 0.00 | |  |
| **ARR *CT_min_*** |  |  |  |
| Intercept | 0.0622 ± 0.0151 | 4.105 | 0.003 |
| Status (indigenous) | 0.0080 ± 0.0277 | 0.290 | 0.779 |
|  | F_(1,8)_ = 0.08, R^2^ = 0.0, _ML_λ = 0.00 | |  |
| **Desiccation** |  |  |  |
| **Log_10_ Time to death** |  |  |  |
| Intercept | 2.265 ± 0.154 | 14.686 | < 0.0001 |
| Status (indigenous) | -0.601 ± 0.244 | -2.464 | 0.039 |
|  | F_(1,8)_ = 6.07, R^2^ = 0.360, _ML_λ = 0.00 | |  |
| **Development rate** |  |  |  |
| **Slope** |  |  |  |
| Intercept | 0.0049 ± 0.0005 | 10.131 | < 0.0001 |
| Status (indigenous) | -0.0008 ± 0.0010 | -0.863 | 0.422 |
|  | F_(1,6)_ = 0.774, R^2^ = 0.0, _ML_λ = 0.00 | |  |
|  |  |  |  |
| **Slope (eV)** |  |  |  |
| Intercept | 0.754 ± 0.052 | 14.613 | < 0.0001 |
| Status (indigenous) | 0.140 ± 0.103 | 1.360 | 0.223 |
|  | F_(1,6)_ = 1.850, R^2^ = 0.108, _ML_λ = 0.00 | |  |
| ***T_opt_*** |  |  |  |
| Intercept | 23.333 ± 1.128 | 20.679 | < 0.0001 |
| Status (indigenous) | -5.833 ± 2.257 | -2.585 | 0.041 |
|  | F_(1,6)_ = 6.682, R^2^ = 0.448, _ML_λ = 0.00 | |  |
| ***U_max_*** |  |  |  |
| Intercept | 0.103 ± 0.011 | 9.621 | < 0.0001 |
| Status (indigenous) | -0.040 ± 0.021 | -1.878 | 0.109 |
|  | F_(1,6)_ = 3.527, R^2^ = 0.265, _ML_λ = 0.00 | |  |
| **Hatching success (ULT50)** |  |  |  |
| Intercept | 23.567 ± 0.943 | 24.989 | < 0.0001 |
| Status (indigenous) | -5.617 ± 1.886 | -2.978 | 0.025 |
|  | F_(1,6)_ = 8.867, R^2^ = 0.529, _ML_λ = 0.00 | |  |

*CT_max_*: critical thermal maximum; *CT_min_*: critical thermal minimum; *CT_range_*: mean *CT_max_* minus mean *CT_min_*; *T_opt_*: optimum temperature: *U_max_*: development rate at the optimum temperature; S.E.: standard error.

**Table 4:** Outcomes of a linear model examining the effects on *CT_max_* and *CT_min_* of week-long acclimation treatments of 5°C, 10°C, 15°C, one hour at -5°C per day (with a background temperature of 10°C), and one hour per day at 25°C (with a background temperature of 10°C). The full model outcome is shown, along with Tukey HSD contrasts for the -5°C extreme vs. 5°C and the 25°C extreme vs. 15°C (for boxplots see Supplementary Figures S4 and S5). The error values are standard error.

| **Species** | ***CT_max_*** |  |  |
| --- | --- | --- | --- |
| **Alien** | **-5°C extreme vs. 5°C** | **25°C extreme vs. 15°C** | **Full model statistics** |
| *C. denticulata* | -0.4 ± 0.1°C, t = -2.8, p = 0.050 | -0.2 ± 0.1°C, t = -1.4, p = 0.650 | F_(4,186)_ = 2.73, p = 0.030 |
| *H. purpurescens* | 0.1 ± 0.1°C, t = 1.4, p = 0.612 | 0.3 ± 0.1°C, t = 3.0, p = 0.026 | F_(4,180)_ = 7.20, p < 0.0001 |
| *H. viatica* | -0.02 ± 0.1°C, t = -0.1, p = 1.0 | 0.1 ± 0.1°C, t = 1.3, p = 0.682 | F_(4,157)_ = 1.63, p = 0.168 |
| *L.* nr. *violaceus* | -0.4 ± 0.1°C, t = -2.9, p = 0.037 | 0.5 ± 0.1°C, t = 3.6, p = 0.003 | F_(4,156)_ = 18.68, p < 0.0001 |
| *P. notabilis* | -0.1 ± 0.1°C, t = -1.6, p = 0.523 | -0.01 ± 0.1°C, t = -0.1, p = 1.0 | F_(4,185)_ = 0.62, p = 0.648 |
| *P. fimata* | 0.3 ± 0.1°C, t = 2.9, p = 0.038 | 0.2 ± 0.1°C, t = 1.5, p = 0.540 | F_(4,186)_ = 5.49, p = 0.001 |
| *Proisotoma* sp. | -0.7 ± 0.2°C, t = -4.3, p < 0.001 | -0.4 ± 0.2°C, t = -2.3, p = 0.158 | F_(4,192)_ = 10.52, p < 0.0001 |
| **Indigenous** |  |  |  |
| *M. caeca* | 0.4 ± 0.1°C, t = 3.0, p = 0.026 | -0.3 ± 0.1°C, t = -2.3, p = 0.159 | F_(4,195)_ = 16.22, p < 0.0001 |
| *P. insularis* | 0.2 ± 0.1°C, t = 1.5, p = 0.568 | 0.2 ± 0.1°C, t = 1.7, p = 0.450 | F_(4,154)_ = 5.83, p < 0.001 |
| *T. bisetosa* | -0.3 ± 0.2°C, t = -1.3, p = 0.686 | 0.1 ± 0.2°C, t = 0.3, p = 0.998 | F_(4,154)_ = 1.67, p = 0.159 |
| **Species** | ***CT_min_*** |  |  |
| **Alien** | **-5°C extreme vs. 5°C** | **25°C extreme vs. 15°C** |  |
| *C. denticulata* | 0.3 ± 0.2°C, t = 1.3, p = 0.711 | -0.1 ± 0.2°C, t = -0.6, p = 0.972 | F_(4,176)_ = 5.65, p < 0.001 |
| *H. purpurescens* | 0.02 ± 0.2°C, t = 0.1, p = 1.0 | -0.3 ± 0.2°C, t = -1.7, p = 0.469 | F_(4,173)_ = 0.84, p = 0.503 |
| *H. viatica* | 0.3 ± 0.2°C, t = 1.5, p = 0.576 | -0.3 ± 0.2°C, t = -1.6, p = 0.513 | F_(4,158)_ = 2.45, p = 0.048 |
| *L.* nr. *violaceus* | 0.4 ± 0.1°C, t = 2.6, p = 0.085 | -0.4 ± 0.2°C, t = -2.7, p = 0.059 | F_(4,155)_ = 17.83, p < 0.0001 |
| *P. notabilis* | -0.6 ± 0.1°C, t = -5.6, p < 0.0001 | -0.4 ± 0.1°C, t = -4.1, p < 0.001 | F_(4,179)_ = 28.3, p < 0.0001 |
| *P. fimata* | 0.3 ± 0.1°C, t = 2.5, p = 0.104 | -0.4 ± 0.1°C, t = -3.0, p = 0.026 | F_(4,181)_ = 11.01, p < 0.0001 |
| *Proisotoma* sp. | -0.1 ± 0.1°C, t = -1.2, p =0.742 | 0.2 ± 0.1°C, t = 1.5, p = 0.582 | F_(4,196)_ = 6.21, p = 0.0001 |
| **Indigenous** |  |  |  |
| *M. caeca* | 0.7 ± 0.1°C, t = 5.6, p < 0.0001 | -0.1 ± 0.1°C, t = -1.0, p = 0.847 | F_(4,190)_ = 25.36, p < 0.0001 |
| *P. insularis* | -0.3 ± 0.1°C, t = -2.6, p = 0.089 | -0.2 ± 0.1°C, t = -1.3, p = 0.699 | F_(4,155)_ = 5.40, p < 0.001 |
| *T. bisetosa* | 0.6 ± 0.2°C, t = 3.1, p = 0.020 | 0.1 ± 0.2°C, t = 0.5, p = 0.985 | F_(4,148)_ = 4.77, p = 0.001 |

**Table 5:** Outcome of a generalised linear model (quasipoisson distribution, log link) comparing individual time to death following desiccation among the alien and indigenous assemblages for the F0 generation trial

|  | **Estimate ± S.E.** | **t** | **p** |
| --- | --- | --- | --- |
| Intercept | 9.748 ± 0.512 | 19.030 | < 0.0001 |
| Status (indigenous) | -0.892 ± 0.136 | -6.537 | < 0.0001 |
| Log_10_ dry mass | 2.265 ± 0.299 | 7.579 | < 0.0001 |
| Residual deviance 63376; df = 323; quasipoisson dispersion parameter = 236.2617 | | |  |

**Table 6:** Mean time to death under desiccating conditions of 76% humidity at 10°C after acclimation for one week at 10°C for the springtail species investigated here. Data for the F0 generation are shown with the exception of two species (marked (F2)).

| **Species** | **n** | **Mean ± S.D.** | **Median** | **Range** |
| --- | --- | --- | --- | --- |
| **Alien** |  |  |  |  |
| *Ceratophysella denticulata* | 30 | 205 ± 85 | 180 | 100 – 380 |
| *Desoria tigrina* | 26 | 116 ± 27 | 120 | 40 – 160 |
| *Hypogastrura purpurescens* (F2) | 40 | 1052 ± 203 | 1005 | 690 – 1420 |
| *Hypogastrura viatica* | 36 | 1064 ± 402 | 970 | 420 – 1740 |
| *Lepidocyrtus* sp. nr. *violaceus* | 21 | 233 ± 111 | 260 | 40 – 410 |
| *Parisotoma notabilis* (F2) | 36 | 20 ± 8 | 20 | 10 – 40 |
| *Protaphorura fimata* | 32 | 132 ± 35 | 135 | 50 – 190 |
| *Proisotoma sp.* | 38 | 80 ± 32 | 80 | 30 – 170 |
| **Indigenous** |  |  |  |  |
| *Mucrosomia caeca* | 34 | 149 ± 34 | 140 | 90 – 220 |
| *Parisotoma insularis* | 23 | 24 ± 9 | 20 | 10 – 40 |
| *Sminthurinus* cf. *tuberculatus* | 30 | 285 ± 103 | 255 | 150 – 440 |
| *Tullbergia bisetosa* | 26 | 47 ± 13 | 40 | 30 - 70 |

**Table 7:** Outcomes of the generalized linear models (quasipoisson distribution, log link) estimating the effects of acclimation and treatment temperature on time to death (as a measure of desiccation resistance) in each of the species examined in this study.

| **Species** | **Estimate ± S.E.** | **t** | **p** |
| --- | --- | --- | --- |
| **Alien** |  |  |  |
| *Ceratophysella denticulata* |  |  |  |
| Intercept | 5.245 ± 0.049 | 107.72 | < 0.0001 |
| Acclimation (20) | 0.371 ± 0.064 | 5.812 | <0.0001 |
| Test temperature (20) | -0.379 ± 0.077 | -4.899 | < 0.0001 |
| Acclimation:Test | -0.566 ± 0.116 | -4.874 | < 0.0001 |
| Residual deviance 3702.8; df = 171; quasipoisson dispersion parameter = 21.57 | | | |
| *Hypogastrura purpurescens* |  |  |  |
| Intercept | 6.958 ± 0.029 | 241.82 | < 0.0001 |
| Acclimation (20) | -0.047 ± 0.042 | -1.122 | 0.264 |
| Test temperature (20) | -1.211 ± 0.061 | -19.964 | < 0.0001 |
| Acclimation:Test | 0.330 ± 0.082 | 4.020 | < 0.0001 |
| Residual deviance 5442.4; df = 152; quasipoisson dispersion parameter = 34.82 | | | |
| *Parisotoma notabilis* |  |  |  |
| Intercept | 2.996 ± 0.064 | 46.94 | < 0.0001 |
| Acclimation (20) | 0.353 ± 0.079 | 4.449 | < 0.0001 |
| Test temperature (20) | -0.553 ± 0.102 | -5.414 | <0.0001 |
| Acclimation:Test | -0.393 ± 0.140 | -2.803 | 0.006 |
| Residual deviance 448.0; df = 156; quasipoisson dispersion parameter = 2.932 | | | |
| *Protaphorura fimata* |  |  |  |
| Intercept | 4.919 ± 0.031 | 160.73 | < 0.0001 |
| Acclimation (20) | 0.002 ± 0.043 | 0.040 | 0.968 |
| Test temperature (20) | -0.556 ± 0.050 | -11.142 | < 0.0001 |
| Acclimation:Test | -0.008 ± 0.070 | -0.115 | 0.908 |
| Residual deviance 754.9; df = 155; quasipoisson dispersion parameter = 4.87 | | | |
| *Proisotoma* sp. |  |  |  |
| Intercept | 4.603 ± 0.038 | 120.16 | < 0.0001 |
| Acclimation (20) | 0.036 ± 0.053 | 0.681 | 0.497 |
| Test temperature (20) | -1.152 ± 0.081 | -14.192 | < 0.0001 |
| Acclimation:Test | 0.547 ± 0.103 | 5.299 | < 0.0001 |
| Residual deviance 1046.2; df = 165; quasipoisson dispersion parameter = 6.294 | | | |
| **Indigenous** |  |  |  |
| *Mucrosomia caeca* |  |  |  |
| Intercept | 4.985 ± 0.035 | 144.55 | < 0.0001 |
| Acclimation (20) | -0.033 ± 0.049 | -0.673 | 0.502 |
| Test temperature (20) | -1.303 ± 0.078 | -16.78 | < 0.0001 |
| Acclimation:Test | 0.757 ± 0.097 | 7.796 | < 0.0001 |
| Residual deviance 1168.7; df = 159; quasipoisson dispersion parameter = 7.30 | | | |
| *Parisotoma insularis* |  |  |  |
| Intercept | 3.209 ± 0.056 | 57.27 | < 0.0001 |
| Acclimation (20) | 0.0001 ± 0.0001 | 0.0 | 1.0 |
| Test temperature (20) | -0.253 ± 0.085 | -2.957 | 0.004 |
| Acclimation:Test | 0.002 ± 0.121 | 0.097 | 0.923 |
| Residual deviance 479.9; df = 152; quasipoisson dispersion parameter = 3.107 | | | |

**Table 8:** Performance curve statistics for egg development rate (1/days to hatching): slope of the linear part of the curve (estimate ± s.e. (n)), the slope given as electron volts from the equation ln rate vs 1/kT (eV), the temperature of the fastest rate recorded (*T_opt_*), the development rate at that temperature (*U_max_*) (mean ± S.D. (n)), and the upper temperature where hatching success declined to 50% (HS ULT50) in springtails from Macquarie Island.

| **Species** | **Slope ± S.E. (n)** | **eV** | ***T_opt_*** | ***U_max_* ± S.D.** | **HS ULT50 ± S.E.** |
| --- | --- | --- | --- | --- | --- |
| **Alien** |  |  |  |  |  |
| *Ceratophysella denticulata* | 0.00595 ± 0.00019 (5) | 0.742 | 25 | 0.13266 ± 0.01027 (36) | 24.5 ± 0.8 |
| *Hypogastrura purpurescens* | 0.00266 ± 0.00026 (5) | 0.744 | 20 | 0.05716 ± 0.00323 (31) | 19.4 ± 0.5 |
| *Hypogastrura viatica* | 0.00530 ± 0.00010 (3) | 0.560 | 25 | 0.10304 ± 0.00783 (45) | 26.3 ± 0.5 |
| *Parisotoma notabilis* | 0.00607 ± 0.00027 (3) | 0.721 | 25 | 0.13516 ± 0.01454 (33) | 25.0 ± 0.5 |
| *Protaphorura fimata* | 0.00445 ± 0.00027 (5) | 0.826 | 25 | 0.09641 ± 0.00717 (42) | 22.8 ± 0.7 |
| *Proisotoma* sp. | 0.00549 ± 0.00031 (4) | 0.928 | 20 | 0.09441 ± 0.00578 (46) | 23.5 ± 0.5 |
| **Indigenous** |  |  |  |  |  |
| *Mucrosomia caeca* | 0.00359 ± 0.00024 (4) | 0.791 | 20 | 0.06440 ± 0.00717 (34) | 19.4 ± 0.5 |
| *Parisotoma insularis* | 0.00468 ± 0.00003 (3) | 0.997 | 15 | 0.06135 ± 0.00206 (44) | 16.5 ± 0.4 |

**Figure legends**

**Figure 1:** Density plots of thermal tolerance in individuals. A. *CT_max_* and B. *CT_min_* for the indigenous and alien assemblages of springtails from Macquarie Island measured in the F0 generation after seven days at 10°C acclimation.

**Figure 2:** Relationship between time to death (log10, minutes) and dry body mass (log10, mg) for individuals of the F0 generation of springtail species from Macquarie Island subject to desiccation trials, indicating substantially greater desiccation resistance, on average, of the alien over the indigenous species. The fitted lines are from a linear model fitted in ggplot2.

**Figure 3:** Boxplots illustrating the effects of different acclimation treatments (10°C (black) or 20°C (orange) for one week) on desiccation resistance (provided here as log_10_ time to death in minutes) measured under 76% relative humidity at a test temperature of either 10°C or 20°C. Summary data are available in Supplementary Table S9.

**Figure 4:** Mean egg development rate (1/days to hatching) between 0°C and 30°C for each of the species investigated here (indigenous species are *M. caeca* and *P. insularis*, the remainder are alien). Where values are zero, this typically indicates no development, or very low hatching success with some development in the case of the values at 0°C. Summary data in Supplementary Table S10.

Figure 1


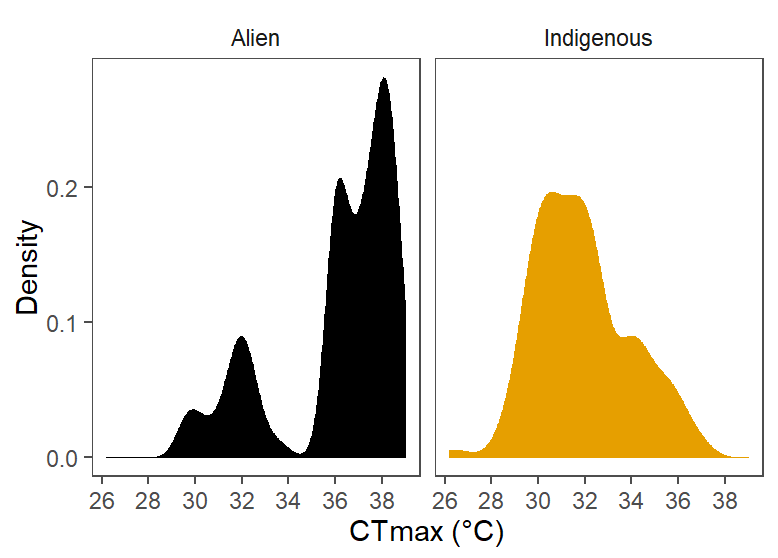


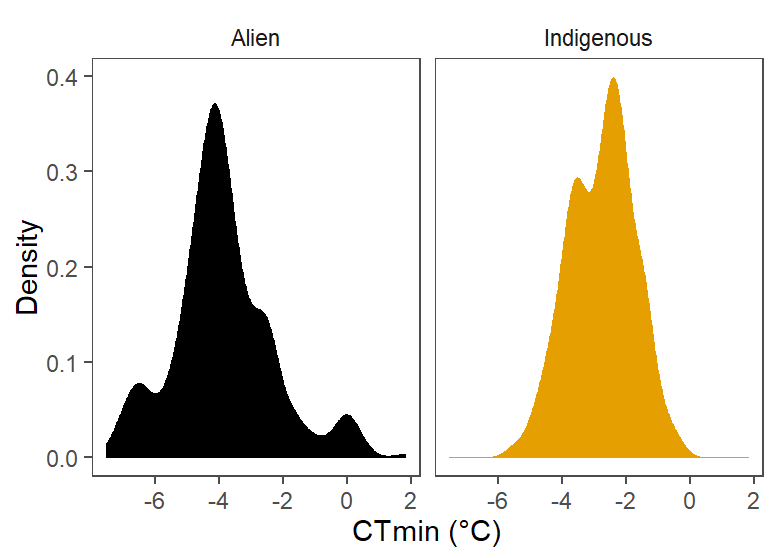


Figure 2


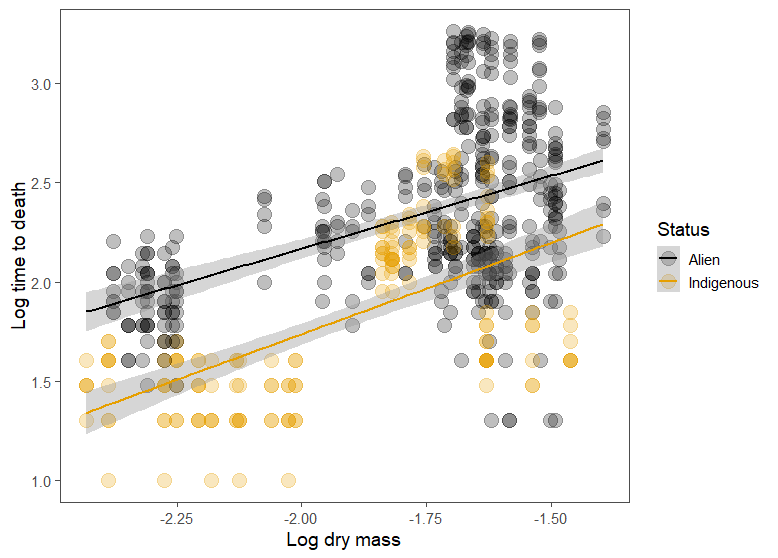


Figure 3


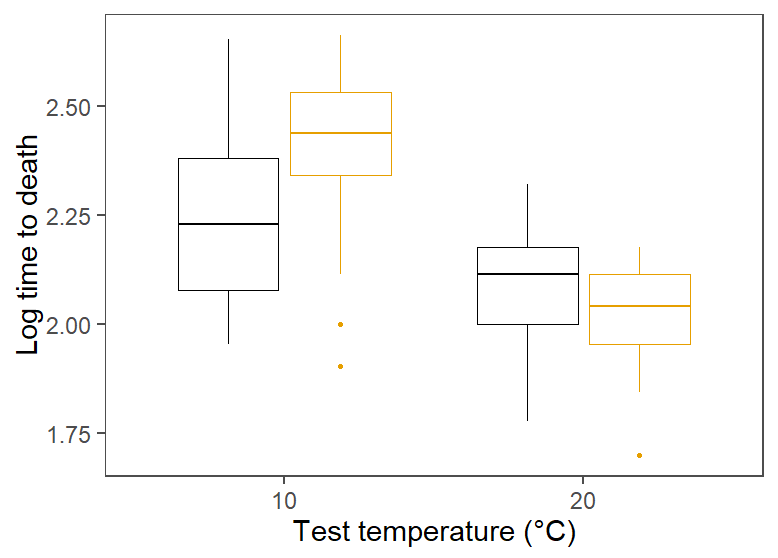
A. *Ceratophysella denticulata* (A) B. *Hypogastrura purpurescens* (A)


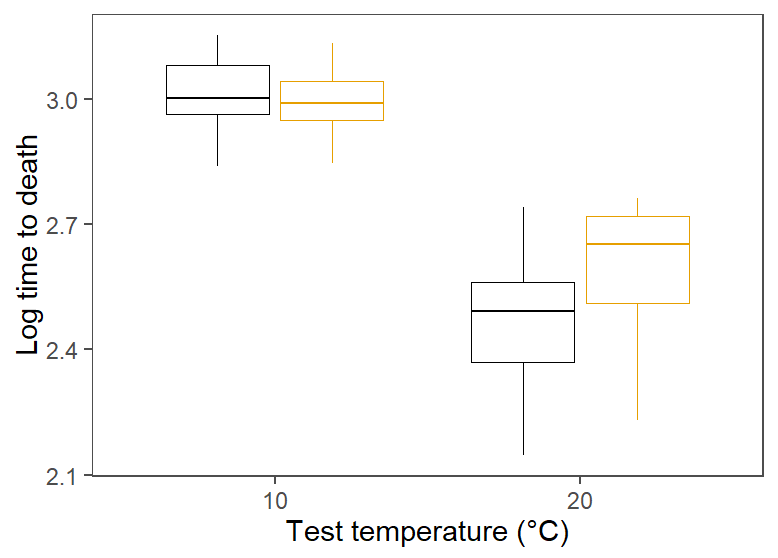


C. *Parisotoma notabilis* (A) D. *Protaphorura fimata* (A)


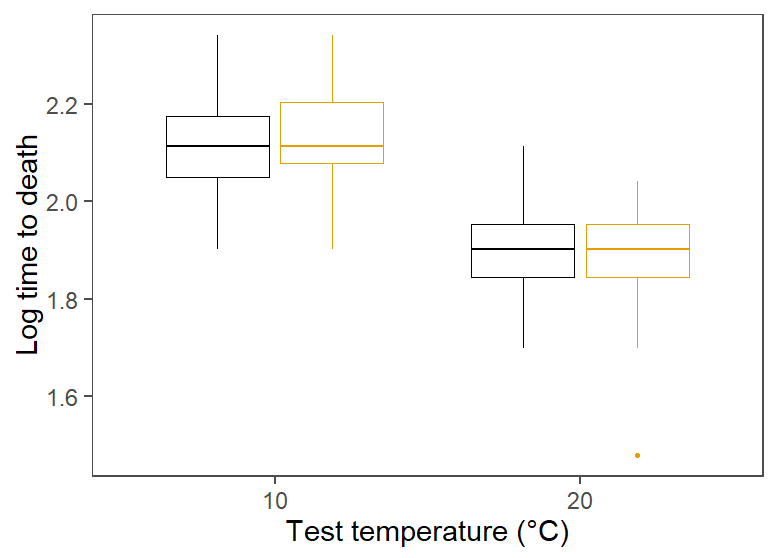

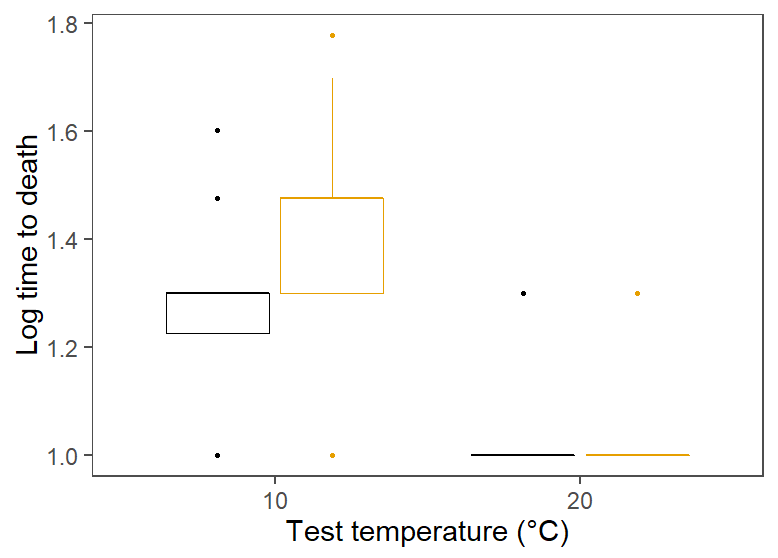


E. *Proisotoma* sp. (A) F. *Mucrosomia caeca* (I)


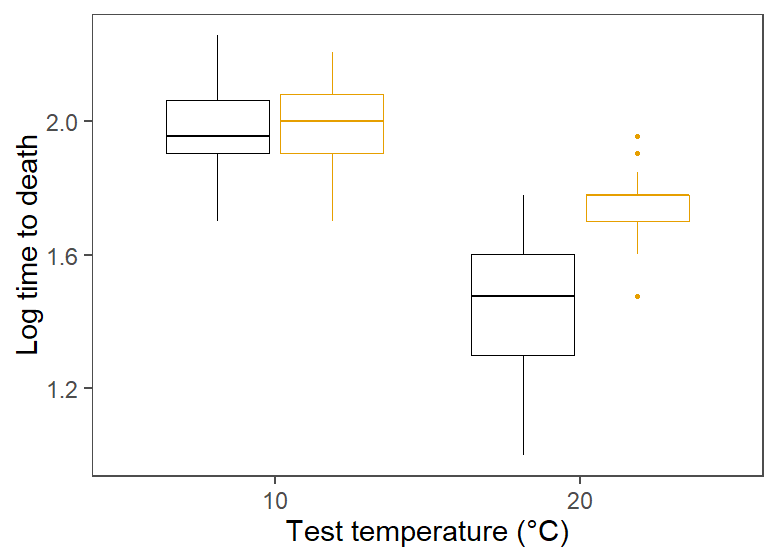


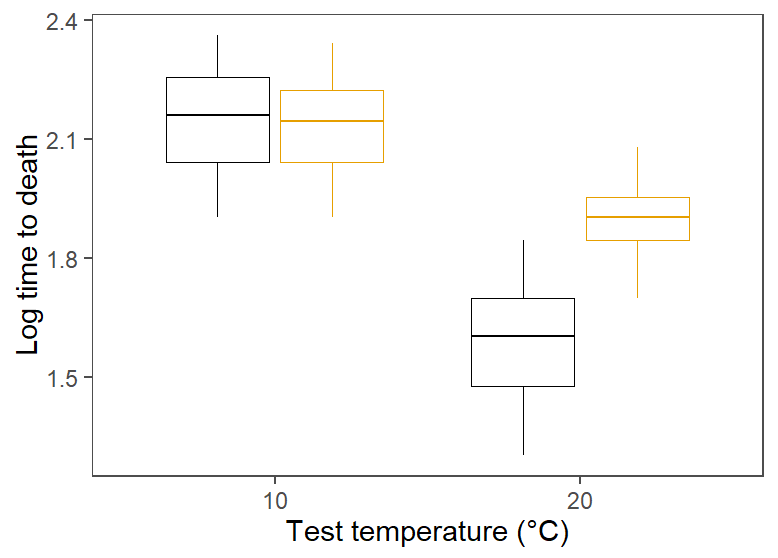


G. *Parisotoma insularis* (I)


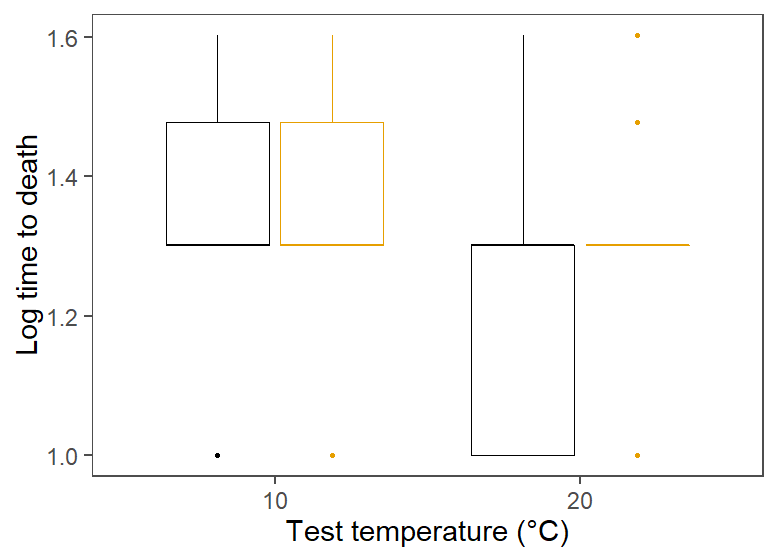


Figure 4


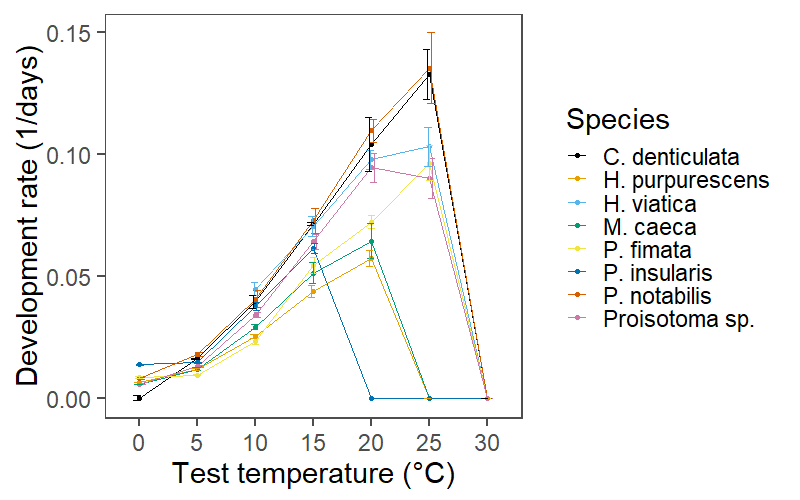

Supplement: suppl_data_coaa049 [file suppl_data_coaa049.zip › Phillips_et_al._ms_rev_marked.docx]
