# Supplementary material for: Basal tolerance but not plasticity gives invasive springtails the advantage in an assemblage setting
Source: Conserv Physiol. 2020 Jun 15;8(1):coaa049. doi: 10.1093/conphys/coaa049 (PMC7294889; doi:10.1093/conphys/coaa049)

**Supplementary Tables and Figures**

**Physiological trait variation among indigenous and invasive species: higher basal tolerance but no difference in plasticity at the assemblage level**

**Laura M. Phillips^1^, Ian Aitkenhead^1^, Charlene Janion-Scheepers^2,3^, Catherine K. King^4^, Melodie A. McGeoch^1^, Uffe N. Nielsen^5^ Aleks Terauds^4^, W.P. Amy Liu^1^, Steven L. Chown^1^**

^1^School of Biological Sciences, Monash University, Victoria 3800, Australia

^2^Iziko South African Museum, Cape Town, 8001, South Africa

^3^Department of Biological Sciences, University of Cape Town, Rondebosch, Cape Town, 7700, South Africa

^4^Australian Antarctic Division, Department of the Environment and Energy, 203 Channel Highway, Kingston, Tasmania 7050, Australia

^4^ Hawkesbury Institute for the Environment, Western Sydney University, Locked Bag 1797, Penrith 2751, New South Wales, Australia

Corresponding author: School of Biological Sciences, Monash University, Victoria 3800, Australia. Tel: +61 3 99050097. Email: [steven.chown@monash.edu](mailto:steven.chown@monash.edu)

**Supplementary Table S1:** Details for samples collected at Macquarie Island during 2016 and 2017.

| **Date** | **Site** | **Latitude** | **Longitude** | **Elevation** | **Sample type** |
| --- | --- | --- | --- | --- | --- |
| 31/3/16 | Green Gorge | -54.6313 | 158.8983 | 28 | Turf x 2, Aspiration |
| 31/3/16 | Green Gorge | -54.6313 | 158.8964 | 28 | Turf x 2, Aspiration |
| 31/3/16 | Green Gorge | -54.6309 | 158.897 | 13 | Turf x 2, Aspiration |
| 1/4/16 | Waterfall Bay | -54.6925 | 158.8647 | 5 | Turf x 2 |
| 1/4/16 | Waterfall Bay | -54.6926 | 158.8644 | 10 | Turf x 2 |
| 1/4/16 | Waterfall Bay | -54.6788 | 158.8715 | 21 | Turf x 2 |
| 2/4/16 | Overland Track | -54.556 | 158.9001 | 215 | Turf x 2 |
| 2/4/16 | Overland Track | -54.539 | 158.915 | 332 | Turf x 2 |
| 3/4/16 | Bauer Bay | -54.5516 | 158.8749 | 31 | Turf x 2 |
| 3/4/16 | Bauer Bay | -54.5526 | 158.8742 | 26 | Turf x 2, Aspiration |
| 3/4/16 | Bauer Bay | -54.5552 | 158.8763 | 35 | Turf x 2, Aspiration |
| 3/4/16 | Sandy Bay | -54.5652 | 158.916 | 19 | Turf x 2 |
| 3/4/16 | Brothers Point | -54.5737 | 158.9182 | 26 | Turf x 2, Aspiration |
| 4/4/16 | East Coast | -54.5508 | 158.9255 | 12 | Turf x 2 |
| 4/4/16 | Nuggets | -54.5297 | 158.9353 | 12 | Turf x 2 |
| 5/4/16 | Lambing Gully | -54.4963 | 158.9422 | 80 | Turf x 2 |
| 3/4/16 | Razorback | -54.5035 | 158.9319 | 11 | Turf x 2 |
| 3/4/16 | Razorback | -54.5032 | 158.9325 | 16 | Turf x 2 |
| 3/4/16 | Hasselborough Bay | -54.501 | 158.9328 | 9 | Turf x 2 |
| 3/4/16 | Razorback | -54.503 | 158.9326 | 6 | Aspiration |
| 3/4/16 | North Head | -54.4973 | 158.9395 | 2 | Turf x 2 |
| 5/4/16 | Lambing Gully | -54.4963 | 158.9422 | 80 | Turf x 2 |
| 5/4/16 | Lambing Gully | -55.2617 | 158.9422 | 80 | Turf x 2 |
| 18/3/17 | Brothers Hut | -54.55557 | 158.87668 | 4 | Turf x 1, Aspiration |
| 19/3/17 | Green Gorge | -54.6304 | 158.89777 | 8 | Aspiration |
| 19/3/17 | Green Gorge | -54.63035 | 158.89774 | 8 | Turf x 2 |
| 19/3/17 | Green Gorge | -54.63186 | 158.89841 | 26 | Turf x 1 |
| 19/3/17 | Waterfall Bay | -54.67879 | 158.87112 | 28 | Turf x 2 |
| 19/3/17 | Waterfall Bay | -54.67919 | 158.8714 | 27 | Aspiration |
| 19/3/17 | Waterfall Bay | -54.69193 | 158.86386 | 19 | Turf x 3 |
| 20/3/17 | Waterfall Bay | -54.69202 | 158.86394 | NA | Turf x 1 |
| 20/3/17 | Waterfall Bay | -54.69201 | 158.86421 | NA | Aspiration |
| 20/3/17 | Green Gorge | -54.62929 | 158.89789 | 13 | Turf x 1, Aspiration |
| 21/2/17 | Bauer Bay | -54.55283 | 158.87624 | 11 | Turf x 2, Aspiration |
| 21/2/17 | Bauer Bay | -54.55527 | 158.8761 | 10 | Turf x 3, Aspiration |
| 22/3/17 | Garden Cove | -54.49889 | 158.94019 | 11 | Aspiration |
| 23/3/17 | Razorback Ridge | -54.50357 | 158.93243 | 13 | Turf x 3, Aspiration |
| 23/3/17 | Razorback Ridge | -54.50444 | 158.9321 | 9 | Turf x 1 |
| 23/3/17 | Razorback Ridge | -54.50459 | 158.93185 | 18 | Turf x 2, Aspiration |
| 21/2/17 | Bauer Bay | -54.55579 | 158.87715 | 28 | Aspiration |
| 21/2/17 | Sandy Bay | -54.56234 | 158.91997 | 9 | Pot |
| 21/2/17 | Sandy Bay | -54.562237 | 158.91986 | 12 | Turf x 1 |
| 21/2/17 | Sandy Bay | -54.5634 | 158.91919 | 20 | Turf x 1, Aspiration |
| 22/3/17 | Island Lake Track Plateau | -54.54042 | 158.88226 | 201 | Turf x 1 |
| 22/3/17 | Island Lake | -54.53089 | 158.88867 | 205 | Turf x 1, Aspiration |
| 22/3/17 | Garden Cove | -54.49892 | 158.94157 | 5 | Turf x 1, Aspiration |
| 22/3/17 | Garden Cove | -54.49855 | 158.94138 | 4 | Turf x 1 |
| 22/3/17 | Garden Cove | -54.49889 | 158.94019 | 11 | Turf x 1 |
| 22/3/17 | Lambing Gully | -54.49767 | 158.94328 | 20 | Turf x 2 |
| 22/3/17 | North Head | -54.49772 | 158.94331 | 25 | Turf x 2 |

**Supplementary Table S2:** List of Macquarie Island springtail species used in experiments, including designated status (indigenous or alien), process ID and BIN numbers from individuals that underwent DNA sequencing at the Biodiversity Institute of Ontario, University of Guelph, Canada. Species locality data is from publicly available information at BOLD.

| **Species** | **Process ID** | **BIN** | **Collections outside of Macquarie Island (from BOLD)** | **Status** |
| --- | --- | --- | --- | --- |
| **Order: Poduromorpha** |  |  |  |  |
| **Family: Onychiuridae** |  |  |  |  |
| *Protaphorura fimata* | COLMU661-16  COLMU662-16  COLMU663-16  COLMU664-16  COLMU665-16  COLMU672-16  COLMU673-16  COLMU674-16  COLMU675-16  COLMU676-16  COLMU677-16 | BOLD:ABV8971  BOLD:ADD5880 BOLD:ABV8970 | France  Norway | Alien |
| **Family: Tullbergiidae** |  |  |  |  |
| *Tullbergia bisetosa* | COLMU577-16  COLMU578-16  COLMU579-16  COLMU580-16  COLMU581-16  COLMU582-16 | BOLD:AAA6992 | South Georgia and the South Sandwich Islands  Marion Island  Tierra del Fuego, Chile | Indigenous |
| **Family: Hypogastruridae** |  |  |  |  |
| *Ceratophysella denticulata* | COLMU589-16  COLMU590-16  COLMU592-16  COLMU593-16  COLMU594-16 | BOLD:AAA9007 BOLD:AAA4803 | Canada  New Zealand  Tasmania | Alien |
| *Hypogastrura viatica* | COLMU601-16  COLMU603-16  COLMU604-16  COLMU605-16  COLMU606-16 | BOLD:AAA4806 | New Zealand  Canada  Norway  Denmark  South Africa  Chile  France  South Georgia and the South Sandwich Islands | Alien |
| *Hypogastrura purpurescens* | COLMU667-16  COLMU669-16  COLMU670-16  COLMU671-16 | BOLD:AAA4804 | New Zealand  Australia  Chile  Argentina  Norway  United Kingdom | Alien |
| **Order: Entomobryomorpha** |  |  |  |  |
| **Family: Isotomidae** |  |  |  |  |
| *Proisotoma* sp. | COLMU649-16  COLMU650-16  COLMU651-16  COLMU652-16  COLMU654-16  COLMU1317-17  COLMU1318-17  COLMU1319-17 | BOLD:AAB4543 | South Africa  New Zealand | Alien |
| *Mucrosomia caeca* | COLMU1299-17  COLMU1300-17  COLMU1301-17  COLMU1302-17  COLMU1303-17  COLMU1304-17 | BOLD:ADI7122 | None | Indigenous |
| *Parisotoma notabilis* | COLMU1320-17  COLMU1321-17  COLMU1322-17 | BOLD:AAA4157 | France  Canada  Spain  Algeria  Australia  Moldova  Italy  South Africa  Denmark  Germany  Russia  Serbia  Turkey  Ukraine | Alien |
| *Parisotoma insularis* | COLMU637-16  COLMU639-16  COLMU640-16  COLMU641-16  COLMU642-16 | BOLD:AAA5791 | New Zealand | Indigenous |
| *Desoria tingria* | COLMU571-16  COLMU572-16  COLMU573-16  COLMU574-16  COLMU575-16  COLMU576-16 | BOLD:AAA5794 | New Zealand | Alien |
| *Folsomotoma punctata* | COLMU625-16  COLMU626-16  COLMU627-16  COLMU628-16  COLMU629-16  COLMU630-16 | BOLD:AAA5795 | South Georgia and the South Sandwich Islands  Marion Island  Heard Island  Diego Ramirez | Indigenous |
| **Family: Entomobryidae** |  |  |  |  |
| *Lepidocyrtus* sp. nr. *violaceus* | COLMU613-16  COLMU614-16  COLMU615-16  COLMU616-16  COLMU617-16  COLMU618-16 | BOLD:AAC0113 BOLD:ADD4535 BOLD:ADD5898 BOLD:ABZ5802 | Canada  United Kingdom  France | Alien |
| *Lepidobrya mawsoni* | COLMU619-16  COLMU620-16  COLMU621-16  COLMU622-16  COLMU623-16  COLMU624-16 | BOLD:AAD3856 | None | Indigenous |
| **Order: Symphypleona** |  |  |  |  |
| **Family: Sminthurididae** |  |  |  |  |
| *Katianna banzarei* | COLMU643-16  COLMU644-16  COLMU645-16  COLMU646-16  COLMU647-16  COLMU648-16 | BOLD:AAC4228 | None | Indigenous |
| *Sminthurinus* cf. *tuberculatus* | COLMU631-16  COLMU632-16  COLMU633-16  COLMU634-16  COLMU636-16 | BOLD:AAD3362 | None | Indigenous |
| **Order: Neelipleona** |  |  |  |  |
| **Family: Neelidae** |  |  |  |  |
| *Megalothorax* sp. | COLMU610-16  COLMU611-16  COLMU1140-17 | BOLD:AAC0897 | France  Belgium  Canada  South Africa  Marion Island | Alien |

**Supplementary Table S3:** Sample sizes for species within each experiment and also a mean body mass (from n = 50 individuals) for each species. Gen. = generation number (where F0 = field caught individuals). Accl. Temp = acclimation temperature (°C) (10 ↓ = 10°C with a temperature drop to -5°C for one hour each day, 10 ↑ = 10°C with a temperature spike to 25°C for one hour each day). (A) = alien, (I) = indigenous. * = species used for development time experiments

| Species |  | *CT_max_* | | | | |  | *CT_min_* | | | | | | Desiccation | | | | | | | | Body  Mass (mg, mean ± sd) |
| --- | --- | --- | --- | --- | --- | --- | --- | --- | --- | --- | --- | --- | --- | --- | --- | --- | --- | --- | --- | --- | --- | --- |
|  | Gen. | F0 | F2 |  |  |  |  | F0 | F2 |  |  |  |  | F0 |  |  |  | F2 |  |  |  |  |
|  | Accl. Temp | 10 | 5 | 10 | 15 | 10 ↓ | 10 ↑ | 10 | 5 | 10 | 15 | 10 ↓ | 10 ↑ | 10 | 20 | 10 | 20 | 10 | 20 | 10 | 20 |  |
|  | Test Temp |  |  |  |  |  |  |  |  |  |  |  |  | 10 | 10 | 20 | 20 | 10 | 10 | 20 | 20 |  |
| *P. fimata* (A) * |  | 33 | 36 | 30 | 30 | 48 | 47 | 33 | 30 | 34 | 31 | 45 | 46 | 32 | 30 |  |  | 38 | 40 | 41 | 40 | 0.1221 ± 0.0268 |
| *T. bisetosa* (I) |  | 31 | 32 | 30 | 31 | 31 | 35 | 30 | 30 | 30 | 30 | 31 | 32 | 26 |  |  |  |  |  |  |  | 0.0764 ± 0.0136 |
| *C. denticulata* (A) * |  | 31 | 37 | 40 | 35 | 37 | 42 | 30 | 43 | 33 | 37 | 32 | 36 | 30 | 21 |  |  | 48 | 46 | 46 | 35 | 0.0571 ± 0.0221 |
| *H. viatica* (A) * |  | 42 | 32 | 32 | 33 | 32 | 33 | 51 | 32 | 33 | 34 | 32 | 32 | 36 | 49 | 40 | 40 |  |  |  |  | 0.1299 ± 0.03884 |
| *H. purpurescens* (A) * |  | 31 | 32 | 30 | 31 | 43 | 49 | 31 | 36 | 31 | 33 | 35 | 43 |  |  |  |  | 40 | 39 | 38 | 39 | 0.2113 ± 0.0613 |
| *Proisotoma* sp. (A) * |  | 32 | 38 | 43 | 31 | 44 | 41 | 40 | 34 | 37 | 35 | 43 | 52 | 38 | 34 |  |  | 43 | 39 | 47 | 40 | 0.0176 ± 0.0036 |
| *M. caeca* (I) * |  | 31 | 32 | 44 | 40 | 40 | 44 | 30 | 36 | 31 | 39 | 43 | 46 | 34 |  |  |  | 42 | 38 | 42 | 41 | 0.0730 ± 0.0205 |
| *P. insularis* (I) * |  | 33 | 30 | 32 | 34 | 31 | 32 | 34 | 33 | 31 | 32 | 31 | 33 | 23 | 24 |  |  | 40 | 39 | 40 | 37 | 0.0376 ± 0.0115 |
| *P. notabilis* (A) * |  | 31 | 39 | 32 | 48 | 33 | 38 | 30 | 36 | 35 | 38 | 33 | 42 |  |  |  |  | 36 | 40 | 46 | 38 | 0.0235 ± 0.0053 |
| *D. tingria* (A) |  | 34 |  |  |  |  |  | 35 |  |  |  |  |  | 26 |  |  |  |  |  |  |  | 0.1388 ± 0.0351 |
| *F. punctata* (I) |  | 35 |  |  |  |  |  | 35 |  |  |  |  |  | 30 |  |  |  |  |  |  |  | 0.0197 ± 0.0053 |
| *L.* sp. nr*. violaceus* (A) |  | 30 | 33 | 35 | 30 | 31 | 32 | 31 | 34 | 32 | 31 | 32 | 31 | 21 | 23 |  |  |  |  |  |  | 0.0674 ± 0.0201 |
| *L. mawsoni* (I) |  | 28 |  |  |  |  |  | 33 |  |  |  |  |  |  |  |  |  |  |  |  |  | 0.3031 ± 0.0916 |
| *K. banzarei* (I) |  | 32 |  |  |  |  |  | 29 |  |  |  |  |  |  |  |  |  |  |  |  |  | 0.1379 ± 0.0270 |
| *S. cf. tuberculatus* (I) |  | 31 |  |  |  |  |  | 30 |  |  |  |  |  | 30 |  |  |  |  |  |  |  | 0.0617 ± 0.0174 |
| *Megalothorax* sp. (A) |  | 29 |  |  |  |  |  | 28 |  |  |  |  |  |  |  |  |  |  |  |  |  | N/A** |

** Could not estimate mass for *Megalothorax* sp. due to small size

**Supplementary Table S4:** Acclimation temperature treatments for springtails in critical thermal limit, desiccation resistance, and development time experiments. Temperature data (°C, [mean ± s.d.]) was recorded with iButton Hygrochron® data loggers (Maxim Integrated, San Jose, USA).

| Experiment | Acclimation Temperatures |
| --- | --- |
| Critical Thermal Limits | 5°C [4.85°C ± 0.24°C]  10°C [10.15°C ± 0.23°C]  15°C [15.09°C ± 0.14°C]  10°C, to 25°C for one hour a day [10: 10.13°C ± 0.18°C; 25: 25.06°C ± 0.21°C]  10°C, to -5°C for one hour a day [10: 9.95°C ± 0.23°C;  -5: -4.7°C ± 0.25°C] |
| Desiccation Resistance | 10°C [10.15°C ± 0.23°C]  20°C [20.02°C ± 0.51°C] |
| Development Time | 0°C [0.02°C ± 0.41°C]  5°C [4.85°C ± 0.24°C]  10°C [10.15°C ± 0.23°C]  15°C [15.09°C ± 0.14°C]  20°C [20.02°C ± 0.51°C]  25°C [24.92°C ± 0.32°C]  30°C [30.09°C ± 0.34°C] |

**Supplementary Table S5:** Summary statistics for species fresh body mass (mg). s.d. = standard deviation; min = minimum; max = maximum.

| **Species** | **n** | **Mean ± s.d.** | **Min** | **Max** |  |
| --- | --- | --- | --- | --- | --- |
| **Alien** |  |  |  |  |  |
| *Ceratophysella denticulata* | 50 | 0.0571 ± 0.02211 | 0.0226 | 0.1149 |  |
| *Desoria tigrina* | 50 | 0.1388 ± 0.03508 | 0.0135 | 0.1988 |  |
| *Hypogastrura purpurescens* | 50 | 0.2113 ± 0.06135 | 0.0898 | 0.3458 |  |
| *Hypogastrura viatica* | 50 | 0.1299 ± 0.03884 | 0.0604 | 0.2204 |  |
| *Lepidocyrtus* sp. nr. *violaceus* | 50 | 0.0674 ± 0.02008 | 0.0282 | 0.1175 |  |
| *Parisotoma notabilis* | 50 | 0.0236 ± 0.00534 | 0.0112 | 0.0342 |  |
| *Protaphorura fimata* | 50 | 0.1221 ± 0.02678 | 0.0745 | 0.1917 |  |
| *Proisotoma* sp. | 50 | 0.0177 ± 0.00362 | 0.0067 | 0.0243 |  |
|  |  |  |  |  |  |
| **Indigenous** |  |  |  |  |  |
| *Folsomatoma punctata* | 50 | 0.0197 ± 0.00530 | 0.0112 | 0.0304 |  |
| *Katianna banzarei* | 50 | 0.1379 ± 0.02699 | 0.0765 | 0.1864 |  |
| *Lepidobrya mawsonii* | 50 | 0.3031 ± 0.09158 | 0.1537 | 0.5310 |  |
| *Mucrosomia caeca* | 50 | 0.0730 ± 0.02045 | 0.0428 | 0.1201 |  |
| *Parisotoma insularis* | 50 | 0.0376 ± 0.01146 | 0.0170 | 0.0633 |  |
| *Sminthurinus cf. tuberculatus* | 50 | 0.0617 ± 0.01743 | 0.0256 | 0.0878 |  |
| *Tullbergia bisetosa* | 50 | 0.0764 ± 0.01360 | 0.0507 | 0.1045 |  |

**Supplementary Table S6:** Summary data (mean ± standard deviation (s.d.); minimum (Min), maximum (Max)) for the effects of constant temperature acclimations (°C) on *CT_max_* (°C) and *CT_min_* (°C).

| **Species** | **Acclimation** | **n** | **Mean ± s.d.** | **Min** | **Max** |
| --- | --- | --- | --- | --- | --- |
| ***CT_max_*** |  |  |  |  |  |
| **Alien** |  |  |  |  |  |
| *Ceratophysella denticulata* | 5 | 37 | 37.9 ± 0.6 | 36.6 | 38.9 |
|  | 10 | 40 | 37.7 ± 0.6 | 36.5 | 39.0 |
|  | 15 | 35 | 37.9 ± 0.5 | 36.5 | 39.1 |
| *Hypogastrura purpurescens* | 5 | 32 | 35.8 ± 0.4 | 35.1 | 36.7 |
|  | 10 | 30 | 36.0 ± 0.3 | 35.4 | 36.5 |
|  | 15 | 31 | 36.0 ± 0.3 | 35.1 | 36.5 |
| *Hypogastrura viatica* | 5 | 32 | 38.3 ± 0.5 | 37.3 | 39.3 |
|  | 10 | 32 | 38.3 ± 0.5 | 37.5 | 39.2 |
|  | 15 | 33 | 38.4 ± 0.4 | 37.4 | 39.4 |
| *Lepidocyrtus* sp. nr. *violaceus* | 5 | 33 | 37.3 ± 0.8 | 35.7 | 38.3 |
|  | 10 | 35 | 37.6 ± 0.8 | 36.5 | 38.5 |
|  | 15 | 30 | 37.6 ± 0.5 | 36.4 | 38.5 |
| *Parisotoma notabilis* | 5 | 39 | 36.1 ± 0.5 | 35.3 | 37.1 |
|  | 10 | 32 | 36.0 ± 0.4 | 35.1 | 37.1 |
|  | 15 | 48 | 36.1 ± 0.4 | 35.3 | 36.8 |
| *Protaphorura fimata* | 5 | 36 | 37.1 ± 0.5 | 36.3 | 37.9 |
|  | 10 | 30 | 37.2 ± 0.6 | 36.2 | 38.4 |
|  | 15 | 30 | 37.4 ± 0.6 | 36.4 | 38.7 |
| *Proisotoma* sp. | 5 | 38 | 36.8 ± 0.7 | 35.3 | 37.8 |
|  | 10 | 43 | 37.0 ± 0.7 | 35.4 | 38.4 |
|  | 15 | 31 | 36.6 ± 0.7 | 35.5 | 38.0 |
| **Indigenous** |  |  |  |  |  |
| *Mucrosomia caeca* | 5 | 32 | 32.8 ± 0.5 | 31.8 | 33.6 |
|  | 10 | 44 | 33.2 ± 0.6 | 31.8 | 34.0 |
|  | 15 | 40 | 33.8 ± 0.6 | 32.4 | 35.2 |
| *Parisotoma insularis* | 5 | 30 | 31.1 ± 0.6 | 29.4 | 32.0 |
|  | 10 | 32 | 31.5 ± 0.6 | 30.5 | 32.1 |
|  | 15 | 34 | 31.5 ± 0.4 | 35.3 | 32.5 |
| *Tullbergia bisetosa* | 5 | 32 | 31.2 ± 1.2 | 28.8 | 32.9 |
|  | 10 | 30 | 31.0 ± 0.9 | 28.8 | 32.3 |
|  | 15 | 31 | 31.3 ± 1.0 | 28.6 | 32.7 |

| ***CT_min_*** |  |  |  |  |  |
| --- | --- | --- | --- | --- | --- |
| **Alien** |  |  |  |  |  |
| *Ceratophysella denticulata* | 5 | 43 | -5.3 ± 0.9 | -6.7 | -3.6 |
|  | 10 | 33 | -4.9 ± 0.8 | -6.1 | -3.3 |
|  | 15 | 37 | -4.5 ± 1.1 | -6.9 | -1.5 |
| *Hypogastrura purpurescens* | 5 | 36 | -4.5 ± 0.7 | -6.3 | -3.6 |
|  | 10 | 31 | -4.6 ± 0.8 | -5.9 | -3.4 |
|  | 15 | 33 | -4.2 ± 0.7 | -5.8 | -3.4 |
| *Hypogastrura viatica* | 5 | 32 | -5.8 ± 0.8 | -7.1 | -4.3 |
|  | 10 | 33 | -5.6 ± 0.9 | -7.1 | -3.9 |
|  | 15 | 34 | -5.2 ± 0.8 | -6.9 | -3.7 |
| *Lepidocyrtus* sp. nr. *violaceus* | 5 | 34 | -4.7 ± 0.5 | -5.8 | -3.7 |
|  | 10 | 32 | -4.0 ± 0.6 | -5.5 | -2.9 |
|  | 15 | 31 | -3.5 ± 0.7 | -4.7 | -2.4 |
| *Parisotoma notabilis* | 5 | 36 | -3.6 ± 0.6 | -4.7 | -2.0 |
|  | 10 | 35 | -3.4 ± 0.5 | -4.4 | -2.3 |
|  | 15 | 38 | -3.1 ± 0.5 | -3.9 | -1.9 |
| *Protaphorura fimata* | 5 | 30 | -3.6 ± 0.5 | -4.7 | -2.5 |
|  | 10 | 34 | -3.5 ± 0.5 | -4.4 | -2.3 |
|  | 15 | 31 | -2.8 ± 0.5 | -3.7 | -1.8 |
| *Proisotoma* sp. | 5 | 34 | -3.3 ± 0.5 | -4.1 | -2.4 |
|  | 10 | 37 | -3.5 ± 0.6 | -4.6 | -2.5 |
|  | 15 | 35 | -3.2 ± 0.5 | -4.4 | -2.2 |
| **Indigenous** |  |  |  |  |  |
| *Mucrosomia caeca* | 5 | 36 | -3.4 ± 0.4 | -4.3 | -2.5 |
|  | 10 | 31 | -2.4 ± 0.6 | -3.7 | -1.1 |
|  | 15 | 39 | -2.2 ± 0.6 | -3.4 | -0.6 |
| *Parisotoma insularis* | 5 | 33 | -3.3 ± 0.7 | -4.6 | -2.1 |
|  | 10 | 31 | -3.2 ± 0.4 | -3.9 | -2.3 |
|  | 15 | 32 | -3.0 ± 0.5 | -4.2 | -1.5 |
| *Tullbergia bisetosa* | 5 | 30 | -3.7 ± 0.9 | -5.2 | -2.0 |
|  | 10 | 30 | -3.2 ± 0.7 | -4.3 | -1.7 |
|  | 15 | 30 | -3.1 ± 0.7 | -4.1 | -1.5 |

**Supplementary Table S7:** Outcomes of a generalized linear model (quasipoisson distribution, log link) estimating the effects of acclimation, treatment temperature and status (alien or indigenous) on time to death (as a measure of desiccation resistance) in each of the species examined in this study. Note the absence of interactions with status, indicating no differences among groups in acclimation effects.

| Species | Estimate ± s.e. | t | p |
| --- | --- | --- | --- |
| Intercept | 5.702 ± 0.062 | 92.151 | < 0.0001 |
| Acclimation (20) | -0.042 ± 0.087 | -0.486 | 0.627 |
| Test temperature (20) | -0.976 ± 0.119 | -8.236 | < 0.0001 |
| Status indigenous | -1.236 ± 0.192 | -6.446 | < 0.0001 |
| Acclimation:Test | 0.215 ± 0.164 | 1.313 | 0.190 |
| Acclimation:Status | 0.014 ± 0.273 | 0.051 | 0.960 |
| Test:Status | -0.110 ± 0.389 | -0.284 | 0.776 |
| Test:Acclimation:Status | 0.391 ± 0.505 | 0.774 | 0.439 |
| Residual deviance 213201; df = 1130; quasipoisson dispersion parameter = 234.96 | | | |

**Supplementary Table S8:** Outcomes of the linear models for the linear part of the egg development rate-temperature relationship for each of the species examined in this study. In each case the lower developmental threshold (LDT, as -intercept/slope) and the sum of effective temperatures (as 1/slope) (see Jarošík et al., 2015) are also provided. s.e. = standard error; LDT = lower developmental threshold (°C); SET = sum of effective temperatures (degree days).

| Species | Estimate ± s.e. | t | p | LDT | SET |
| --- | --- | --- | --- | --- | --- |
| **Alien** |  |  |  |  |  |
| *Ceratophysella denticulata* |  |  |  |  |  |
| Intercept | -0.0164 ± 0.0031 | -5.539 | 0.0127 | 2.78 | 168.05 |
| Temperature | 0.0060 ± 0.0001 | 32.055 | < 0.0001 |  |  |
| F_(1,3)_ = 1028, R^2^ = 0.996, p < 0.0001 | | | |  |  |
| *Hypogastrura purpurescens* |  |  |  |  |  |
| Intercept | 0.0023 ± 0.0032 | 0.724 | 0.522 | -0.87 | 375.47 |
| Temperature | 0.0027 ± 0.0003 | 10.236 | 0.0020 |  |  |
| F_(1,3)_ = 104.8, R^2^ = 0.963, p = 0.002 | | | |  |  |
| *Hypogastrura viatica* |  |  |  |  |  |
| Intercept | -0.0085 ± 0.0016 | -5.341 | 0.118 | 1.61 | 188.87 |
| Temperature | 0.0053± 0.0001 | 51.634 | 0.0123 |  |  |
| F_(1,1)_ = 2666, R^2^ = 0.999, p = 0.0123 | |  |  |  |  |
| *Parisotoma notabilis* |  |  |  | 2.60 | 164.83 |
| Intercept | -0.0158 ± 0.044 | -3.581 | 0.037 |  |  |
| Temperature | 0.0061 ± 0.0003 | 22.833 | 0.0002 |  |  |
| F_(1,3)_ = 521.3, R^2^ = 0.992, p = 0.0002 | | | |  |  |
| *Protaphorura fimata* |  |  |  |  |  |
| Intercept | -0.0156 ± 0.044 | -3.156 | 0.039 | 3.50 | 224.54 |
| Temperature | 0.0045 ± 0.0003 | 16.641 | 0.0005 |  |  |
| F_(1,3)_ = 276.9, R^2^ = 0.986, p = 0.0004 | | | |  |  |
| *Proisotoma* sp. |  |  |  |  |  |
| Intercept | -0.0172 ± 0.0043 | -4.104 | 0.0568 | 3.13 | 182.29 |
| Temperature | 0.0055 ± 0.0003 | 17.564 | = 0.0032 |  |  |
| F_(1,2)_ = 308.5, R^2^ = 0.990, p = 0.0032 | | | |  |  |
| **Indigenous** |  |  |  |  |  |
| *Mucrosomia caeca* |  |  |  |  |  |
| Intercept | -0.0057 ± 0.0033 | -1.725 | 0.227 | 1.58 | 278.51 |
| Temperature | 0.0036 ± 0.0002 | 15.004 | 0.004 |  |  |
| F_(1,2)_ = 225.1, R^2^ = 0.987, p = 0.004 | | | |  |  |
| *Parisotoma insularis* |  |  |  |  |  |
| Intercept | -0.0090 ± 0.0004 | -25.15 | 0.0253 | 1.92 | 213.54 |
| Test temperature (20) | 0.0047 ± 0.00003 | 141.5 | 0.005 |  |  |
| F_(1,1)_ = 2000, R^2^ = 0.999, p = 0.005 | | | |  |  |

**Supplementary Table S9:** Outcomes of the acclimation treatments (7 days at the acclimation treatment) on desiccation resistance (in minutes) at each of two test temperatures. s.d. = standard deviation; min = minimum; max = maximum.

| **Species** | **Test temperature (°C)** | **Acclimation temperature (°C)** | **n** | **Mean** | **s.d.** | **Median** | **Min** | **Max** |
| --- | --- | --- | --- | --- | --- | --- | --- | --- |
| **Alien** |  |  |  |  |  |  |  |  |
| *Ceratophysella denticulata* | 10 | 10 | 48 | 189.6 | 83.7 | 170 | 90 | 450 |
|  | 10 | 20 | 46 | 274.8 | 90.1 | 275 | 80 | 460 |
|  | 20 | 10 | 46 | 129.8 | 34.7 | 130 | 60 | 210 |
|  | 20 | 20 | 35 | 106.9 | 25.5 | 110 | 50 | 150 |
| *Hypogastrura purpurescens* | 10 | 10 | 40 | 1051.5 | 202.6 | 1005 | 690 | 1420 |
|  | 10 | 20 | 38 | 1003.4 | 168.1 | 975 | 700 | 1360 |
|  | 20 | 10 | 39 | 313.3 | 101.0 | 310 | 140 | 550 |
|  | 20 | 20 | 39 | 415.90 | 127.8 | 450 | 170 | 580 |
| *Parisotoma notabilis* | 10 | 10 | 36 | 20.0 | 7.6 | 20 | 10 | 40 |
|  | 10 | 20 | 46 | 28.5 | 13.3 | 30 | 10 | 60 |
|  | 20 | 10 | 40 | 11.5 | 3.6 | 10 | 10 | 20 |
|  | 20 | 20 | 38 | 11.1 | 3.1 | 10 | 10 | 20 |
| *Protaphorura fimata* | 10 | 10 | 38 | 136.8 | 30.9 | 130 | 80 | 220 |
|  | 10 | 20 | 41 | 137.1 | 28.7 | 130 | 80 | 220 |
|  | 20 | 10 | 40 | 78.5 | 16.3 | 80 | 50 | 130 |
|  | 20 | 20 | 40 | 78.0 | 15.7 | 80 | 30 | 110 |
| *Proisotoma* sp. | 10 | 10 | 43 | 99.8 | 27.7 | 90 | 50 | 180 |
|  | 10 | 20 | 47 | 103.4 | 31.0 | 100 | 50 | 160 |
|  | 20 | 10 | 39 | 31.5 | 12.9 | 30 | 10 | 60 |
|  | 20 | 20 | 40 | 56.5 | 11.2 | 60 | 30 | 90 |
| **Indigenous** |  |  |  |  |  |  |  |  |
| *Mucrosomia caeca* | 10 | 10 | 42 | 146.2 | 41.6 | 145 | 80 | 230 |
|  | 10 | 20 | 42 | 141.4 | 36.7 | 140 | 80 | 220 |
|  | 20 | 10 | 38 | 39.7 | 12.8 | 40 | 20 | 70 |
|  | 20 | 20 | 41 | 82.0 | 16.5 | 80 | 50 | 120 |
| *Parisotoma insularis* | 10 | 10 | 40 | 24.8 | 8.8 | 20 | 10 | 40 |
|  | 10 | 20 | 40 | 24.8 | 10.1 | 20 | 10 | 40 |
|  | 20 | 10 | 39 | 19.2 | 7.4 | 20 | 10 | 40 |
|  | 20 | 20 | 37 | 19.5 | 6.6 | 20 | 10 | 40 |

**Supplementary Table S10:** Egg development rate (1/days to hatching) at the test temperatures for each of the eight springtail species investigated in this study. s.d. = standard deviation; min = minimum; max = maximum.

| **Species** | **Temperature (°C)** | **n** | **Mean** | **s.d.** | **Median** | **Min** | **Max** |
| --- | --- | --- | --- | --- | --- | --- | --- |
| **Indigenous** |  |  |  |  |  |  |  |
| *Ceratophysella denticulata* | 0 | 50 | 0.00014599 | 0.001032 | 0.00000000 | 0.00000000 | 0.00729927 |
|  | 5 | 39 | 0.01620776 | 0.000290 | 0.01612903 | 0.01562500 | 0.01666667 |
|  | 10 | 40 | 0.03949140 | 0.002761 | 0.03846154 | 0.03571429 | 0.05000000 |
|  | 15 | 49 | 0.07133139 | 0.000680 | 0.07142857 | 0.06666667 | 0.07142857 |
|  | 20 | 39 | 0.10411440 | 0.010977 | 0.10000000 | 0.09090909 | 0.14285714 |
|  | 25 | 36 | 0.13266093 | 0.010273 | 0.12500000 | 0.11111111 | 0.14285714 |
|  | 30 | 50 | NA | NA | NA | NA | NA |
| *Hypogastrura purpurescens* | 0 | 37 | 0.00658714 | 0.000089 | 0.00662252 | 0.00645161 | 0.00671141 |
|  | 5 | 47 | 0.01179359 | 0.000313 | 0.01190476 | 0.01098901 | 0.01250000 |
|  | 10 | 45 | 0.02535471 | 0.000874 | 0.02564103 | 0.02272727 | 0.02631579 |
|  | 15 | 43 | 0.04380869 | 0.002502 | 0.04545455 | 0.03703704 | 0.04545455 |
|  | 20 | 31 | 0.05716222 | 0.003234 | 0.05555556 | 0.05000000 | 0.06250000 |
|  | 25 | 50 | NA | NA | NA | NA | NA |
|  | 30 | 50 | NA | NA | NA | NA | NA |
| *Hypogastrura viatica* | 10 | 50 | 0.04472854 | 0.002766 | 0.04545455 | 0.04000000 | 0.05263158 |
|  | 15 | 48 | 0.07031345 | 0.004162 | 0.07142857 | 0.06250000 | 0.07692308 |
|  | 20 | 43 | 0.09767442 | 0.004013 | 0.10000000 | 0.09090909 | 0.10000000 |
|  | 25 | 45 | 0.10303591 | 0.007827 | 0.10000000 | 0.09090909 | 0.12500000 |
|  | 30 | 50 | NA | NA | NA | NA | NA |
| *Parisotoma notabilis* | 0 | 30 | 0.00821201 | 0.000301 | 0.00833333 | 0.00769231 | 0.00854701 |
|  | 5 | 44 | 0.01796758 | 0.000740 | 0.01818182 | 0.01639344 | 0.01923077 |
|  | 10 | 47 | 0.04067736 | 0.003401 | 0.04000000 | 0.03333333 | 0.05000000 |
|  | 15 | 50 | 0.07267757 | 0.005179 | 0.07142857 | 0.06250000 | 0.09090909 |
|  | 20 | 43 | 0.10962532 | 0.004786 | 0.11111111 | 0.10000000 | 0.12500000 |
|  | 25 | 33 | 0.13516114 | 0.014544 | 0.14285714 | 0.11111111 | 0.16666667 |
|  | 30 | 50 | NA | NA | NA | NA | NA |
| *Protaphorura fimata* | 0 | 30 | 0.00859199 | 0.000276 | 0.00851079 | 0.00833333 | 0.00917431 |
|  | 5 | 46 | 0.00957022 | 0.000326 | 0.00952381 | 0.00909091 | 0.01030928 |
|  | 10 | 46 | 0.02324498 | 0.001295 | 0.02325581 | 0.02040816 | 0.02564103 |
|  | 15 | 49 | 0.05451651 | 0.003254 | 0.05555556 | 0.04761905 | 0.06250000 |
|  | 20 | 43 | 0.07225062 | 0.002842 | 0.07142857 | 0.06666667 | 0.08333333 |
|  | 25 | 42 | 0.09640452 | 0.007168 | 0.10000000 | 0.08333333 | 0.11111111 |
|  | 30 | 50 | NA | NA | NA | NA | NA |
| *Proisotoma* sp. | 0 | 7 | 0.00558241 | 0.000042 | 0.00558659 | 0.00552486 | 0.00561798 |
|  | 5 | 46 | 0.01296545 | 0.000482 | 0.01307245 | 0.01098901 | 0.01369863 |
|  | 10 | 47 | 0.03414528 | 0.001090 | 0.03448276 | 0.02857143 | 0.03571429 |
|  | 15 | 47 | 0.06410108 | 0.003159 | 0.06250000 | 0.05263158 | 0.07142857 |
|  | 20 | 46 | 0.09441151 | 0.005780 | 0.09090909 | 0.08333333 | 0.11111111 |
|  | 25 | 19 | 0.08991535 | 0.008208 | 0.09090909 | 0.07692308 | 0.10000000 |
|  | 30 | 50 | NA | NA | NA | NA | NA |
| *Mucrosomia caeca* | 0 | 8 | 0.00561815 | 0.000034 | 0.00561798 | 0.00555556 | 0.00564972 |
|  | 5 | 42 | 0.01193590 | 0.000639 | 0.01176471 | 0.01075269 | 0.01408451 |
|  | 10 | 44 | 0.02921867 | 0.001121 | 0.02899160 | 0.02777778 | 0.03225807 |
|  | 15 | 41 | 0.05136935 | 0.004261 | 0.05000000 | 0.03846154 | 0.05882353 |
|  | 20 | 34 | 0.06439609 | 0.007170 | 0.06666667 | 0.04545455 | 0.07142857 |
|  | 25 | 50 | NA | NA | NA | NA | NA |
|  | 30 | 50 | NA | NA | NA | NA | NA |
| *Parisotoma insularis* | 0 | 45 | 0.01379749 | 0.000179 | 0.01388889 | 0.01333333 | 0.01408451 |
|  | 5 | 47 | 0.01452087 | 0.000136 | 0.01449275 | 0.01428571 | 0.01470588 |
|  | 10 | 49 | 0.03764917 | 0.001661 | 0.03703704 | 0.03448276 | 0.04000000 |
|  | 15 | 44 | 0.06135064 | 0.002064 | 0.06250000 | 0.05555556 | 0.06666667 |
|  | 20 | 50 | NA | NA | NA | NA | NA |
|  | 25 | 50 | NA | NA | NA | NA | NA |
|  | 30 | 50 | NA | NA | NA | NA | NA |

**Supplementary Figure S1:** The phylogenetic tree used for phylogenetic generalised least squares analyses in this study. Where smaller groups of species were assessed, the tree was appropriately trimmed and branch lengths recalculated. Alien species: Cdent *Ceratophysella denticulata*; Dtigr *Desoria tigrina*; Hpurp *Hypogastrura purpurescens*; Hviat *Hypogastrura viatica*; Lviol *Lepidocyrtus sp. nr. violaceus*; Mmini *Megalothorax nr. minimus*; Pnota *Parisotoma notabilis*; Pfima *Protaphorura fimata*; Prois *Proisotoma* sp.. Indigenous species: Fpunc *Folsomatoma punctata*; Kbanz *Katianna banzarei*; Lmaws *Lepidobrya mawsonii*; Mcaec *Mucrosomia caeca*; Pinsu *Parisotoma insularis*; Stube *Sminthurinus cf. tuberculatus*; Tbise *Tullbergia bisetosa*.


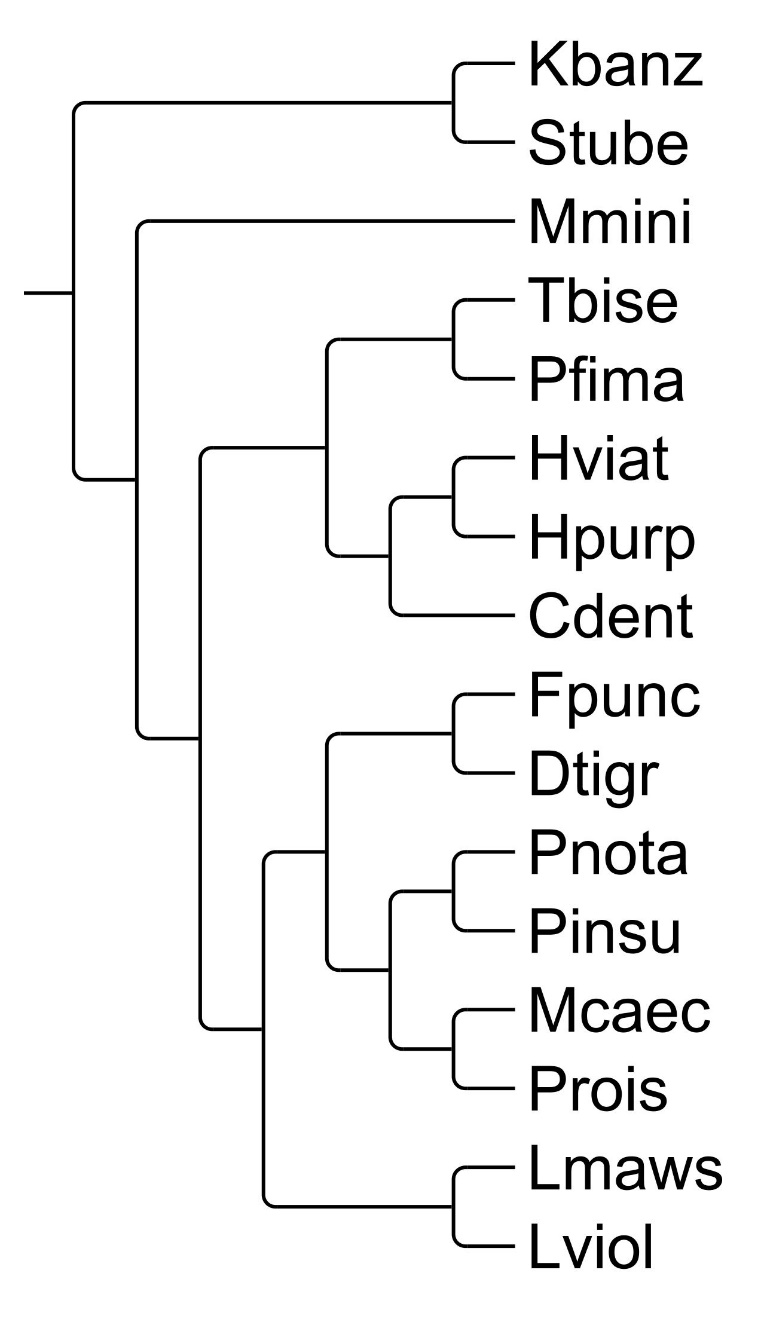


**Supplementary Figure S2:** Frequency distribution of time to death for the desiccation trials indicating the bounded and right-skewed form of the data.


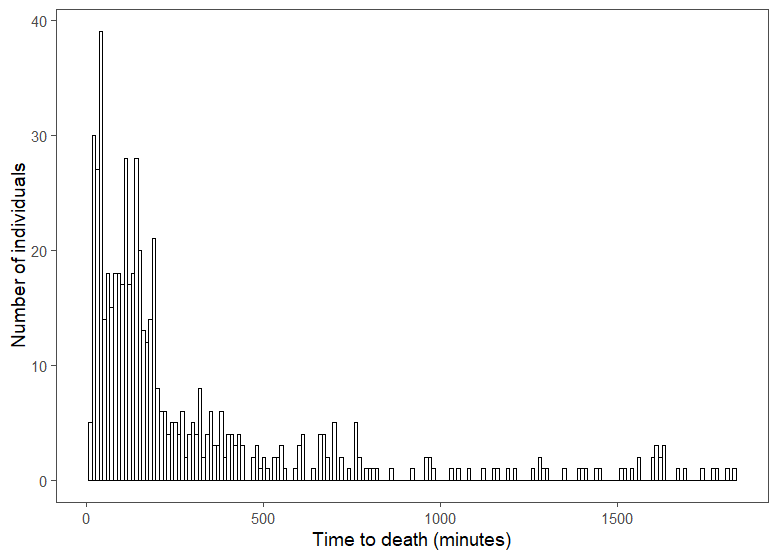


**Supplementary Figure S3:** Density plots for individual data for each of the 16 species examined for A. *CT_max_* and B. *CT_min_* for the indigenous (green) and alien (red) assemblages measured in the F0 generation after seven days at 10°C acclimation.

A.

B.

**Supplementary Figure S4:** Boxplots illustrating the effects of different acclimation treatments on critical thermal maximum (*CT_max_*). Acclimation treatments were one week at constant 5°C, 10°C, or 15°C; or extreme acclimation treatments of -5°C for one hour per day against a background of constant 10°C (105), or 25°C for one hour per day against a background of constant 10°C (1025). A= alien, I = indigenous.

A. *Ceratophysella denticulata* (A) B. *Hypogastrura purpurescens* (A)


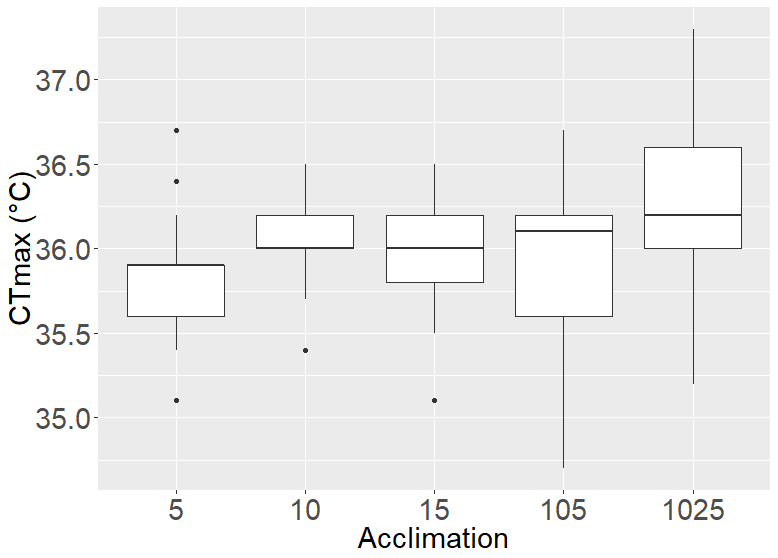

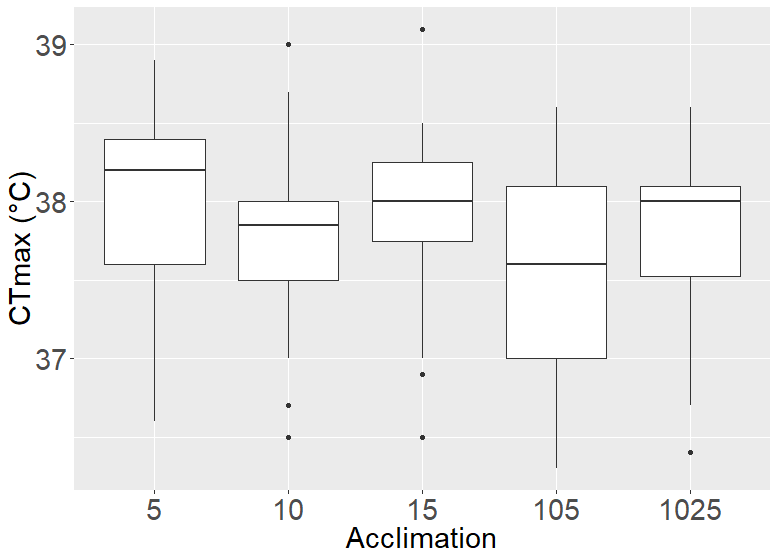


C. *Hypogastrura viatica* (A) D. *Lepidocyrtus sp. nr violaceus* (A)


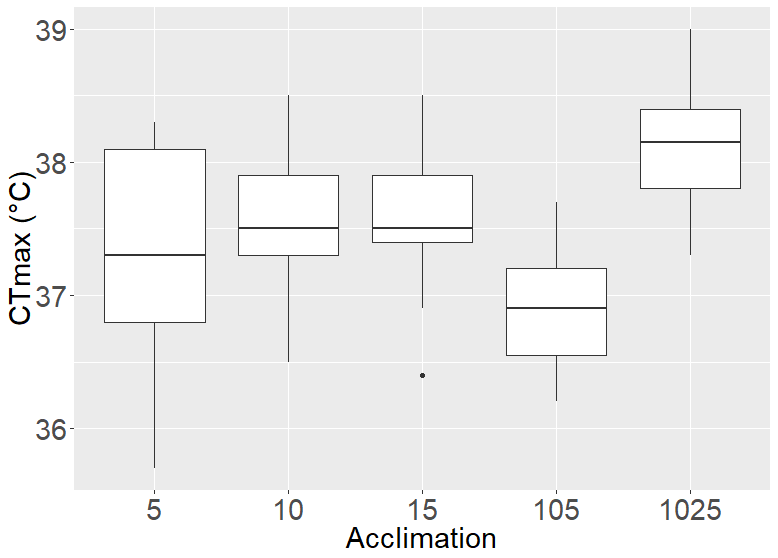

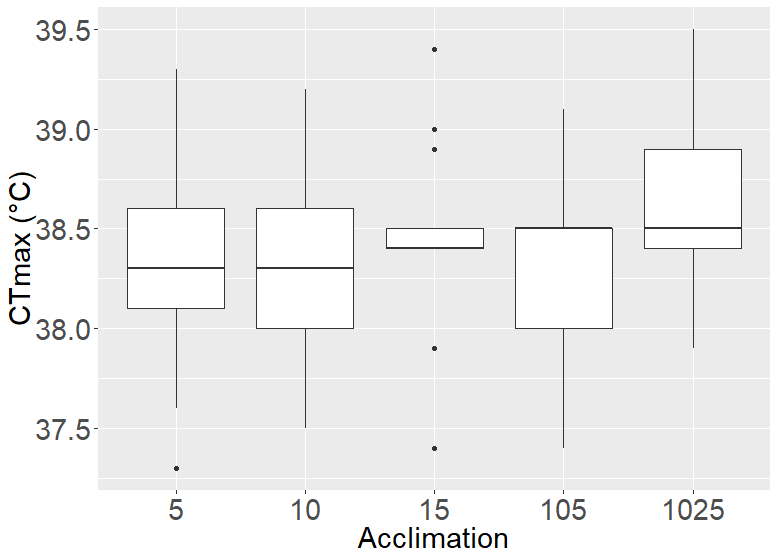


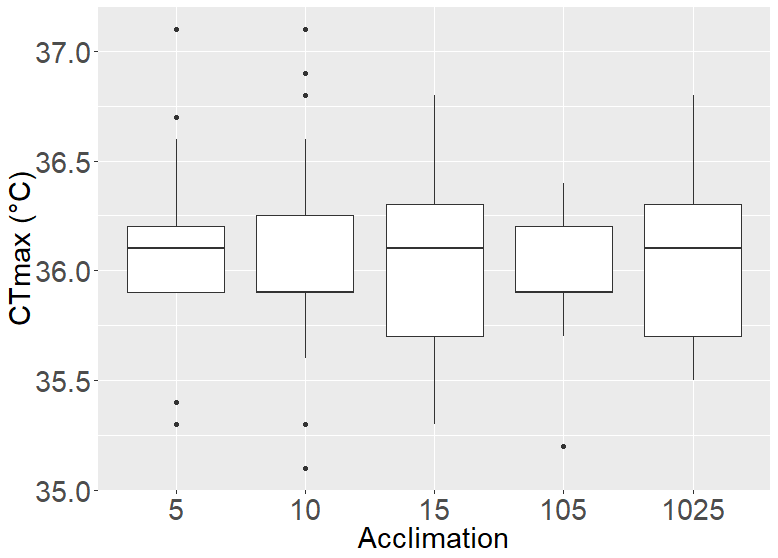
E. *Parisotoma notabilis* (A) F. *Protaphorura fimata* (A)


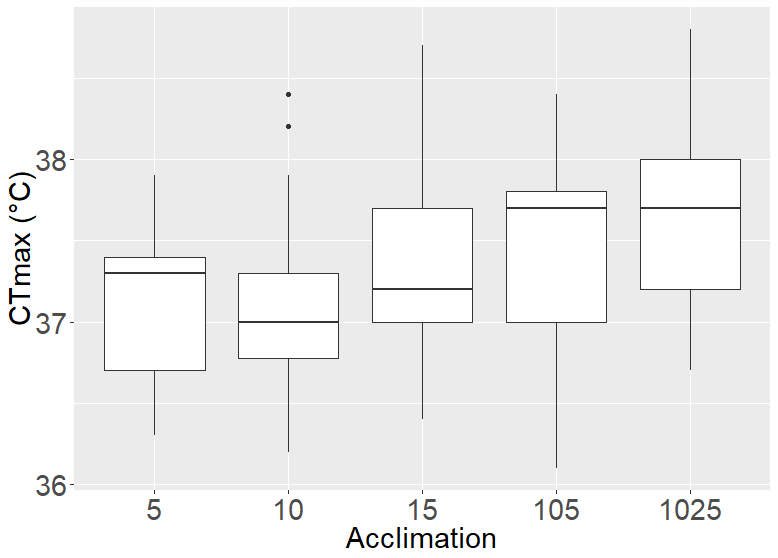


G. *Proisotoma* sp. (A) H. *Mucrosomia caeca* (I)


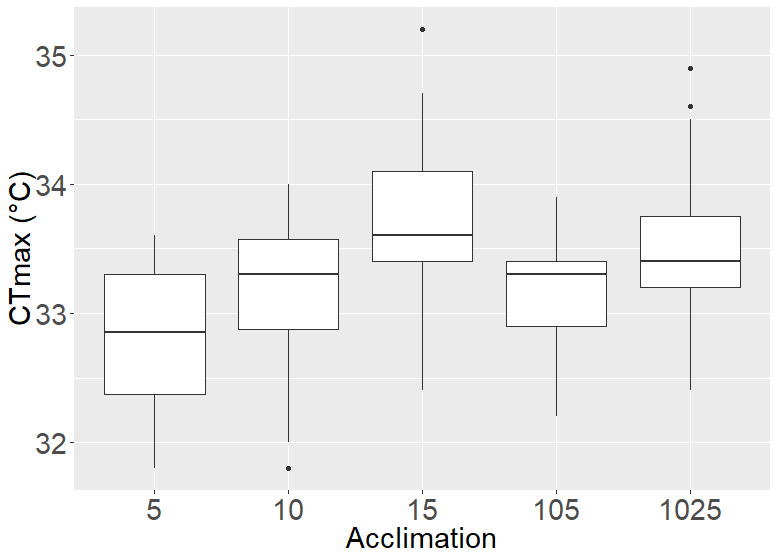

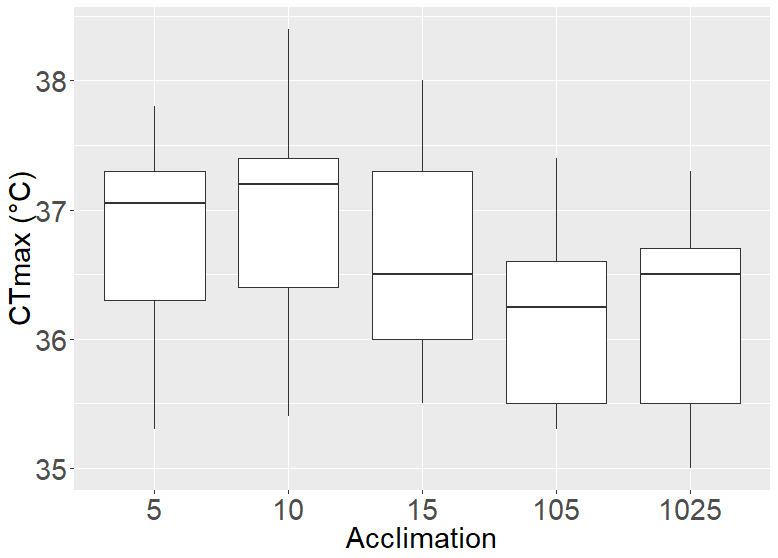


I. *Parisotoma insularis* (I) J. *Tullbergia bisetosa* (I)


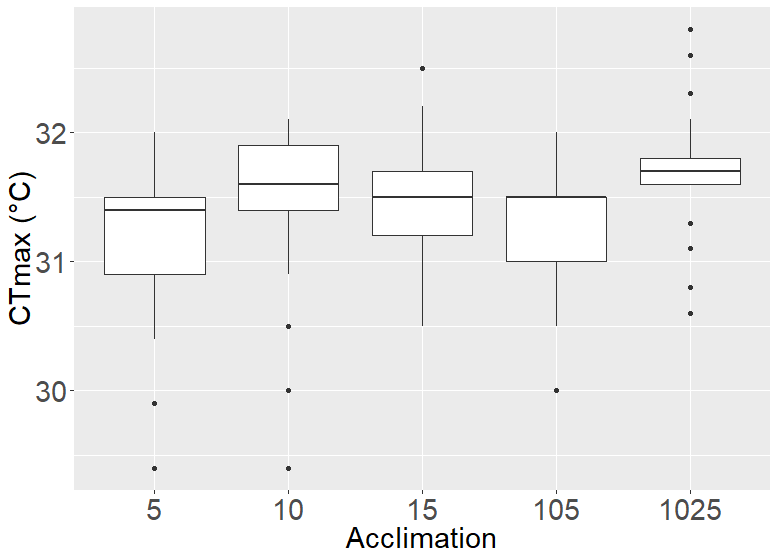


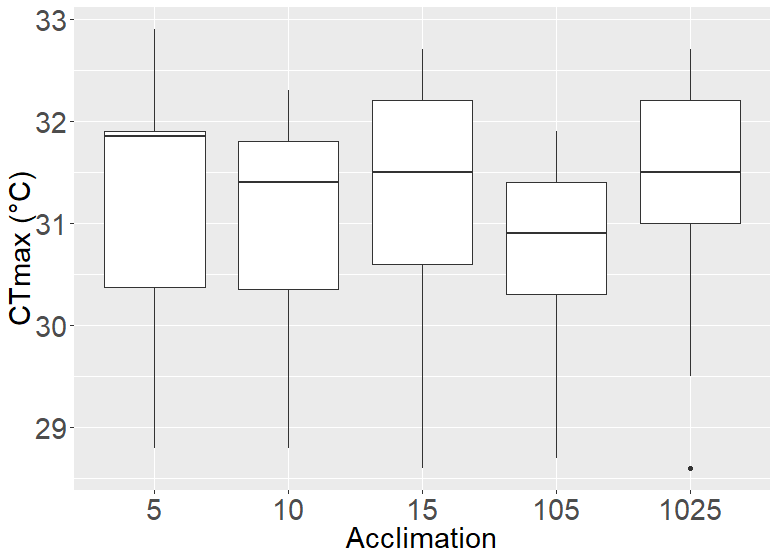


**Supplementary Figure S5:** Boxplots illustrating the effects of different acclimation treatments on critical thermal minimum (*CT_min_*). Acclimation treatments were one week at constant 5°C, 10°C, or 15°C; or extreme acclimation treatments of -5°C for one hour per day against a background of constant 10°C (105), or 25°C for one hour per day against a background of constant 10°C (1025). A= alien, I = indigenous.

A. *Ceratophysella denticulata* (A) B. *Hypogastrura purpurescens* (A)


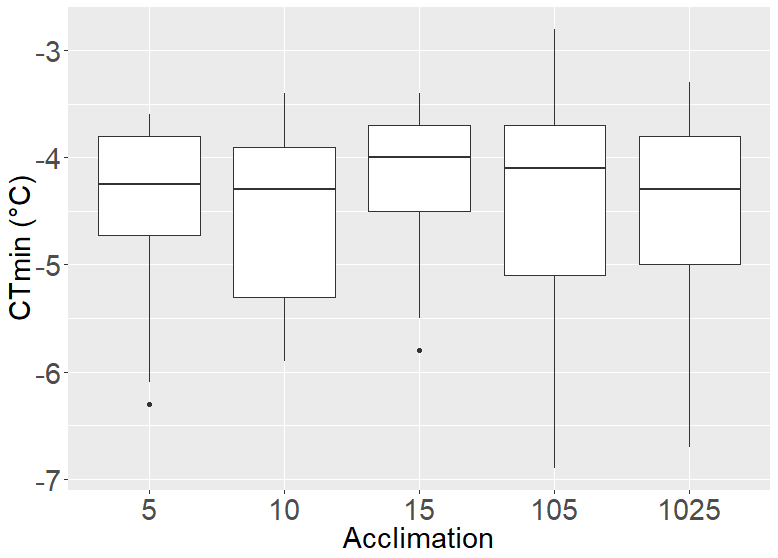

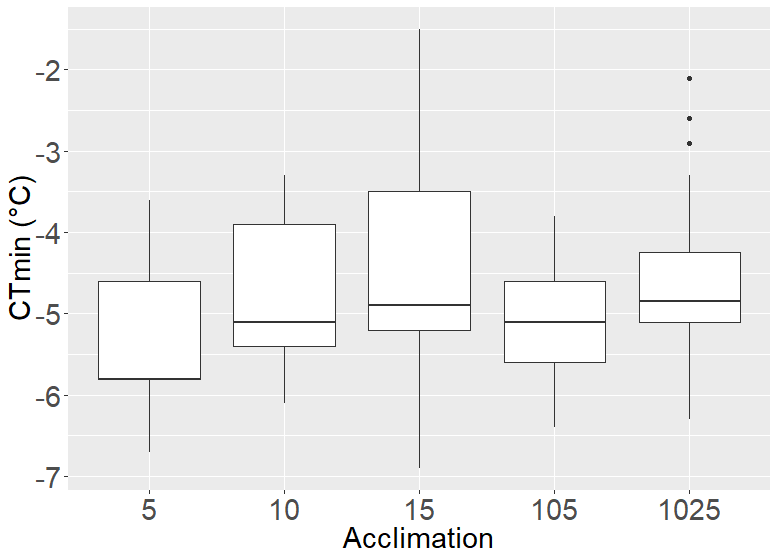


C. *Hypogastrura viatica* (A) D. *Lepidocyrtus* sp. nr. *violaceus* (A)


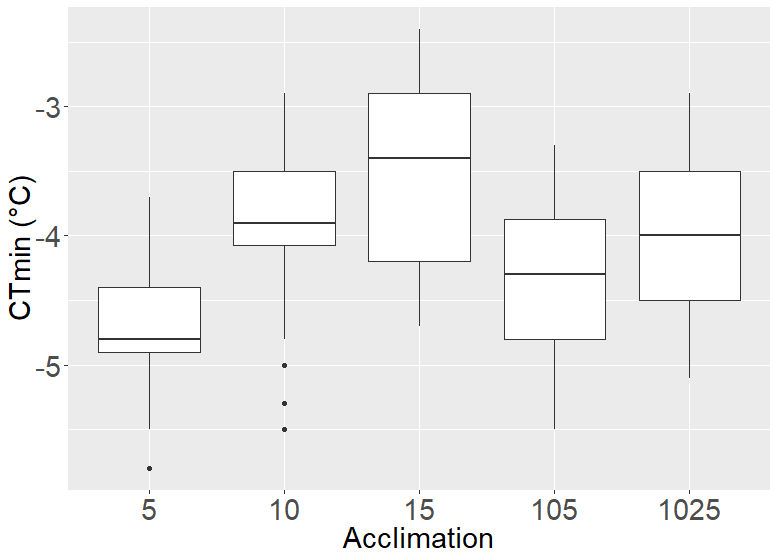

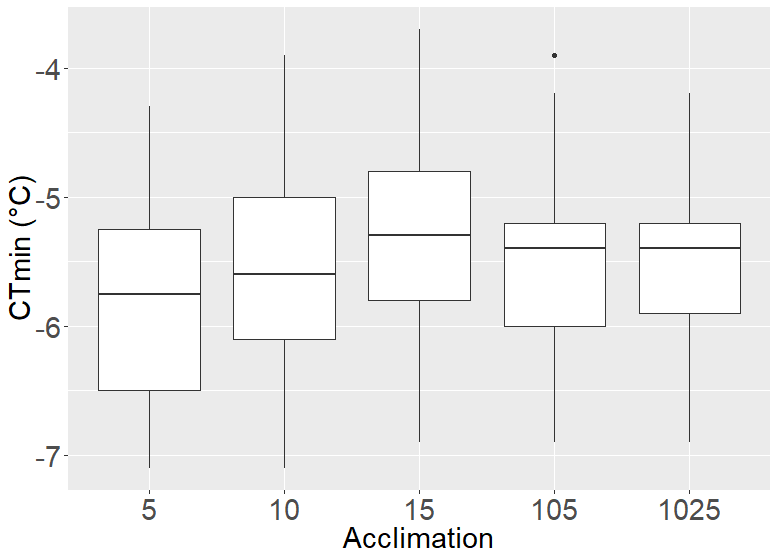


E. *Parisotoma notabilis* (A) F. *Protaphorura fimata* (A)


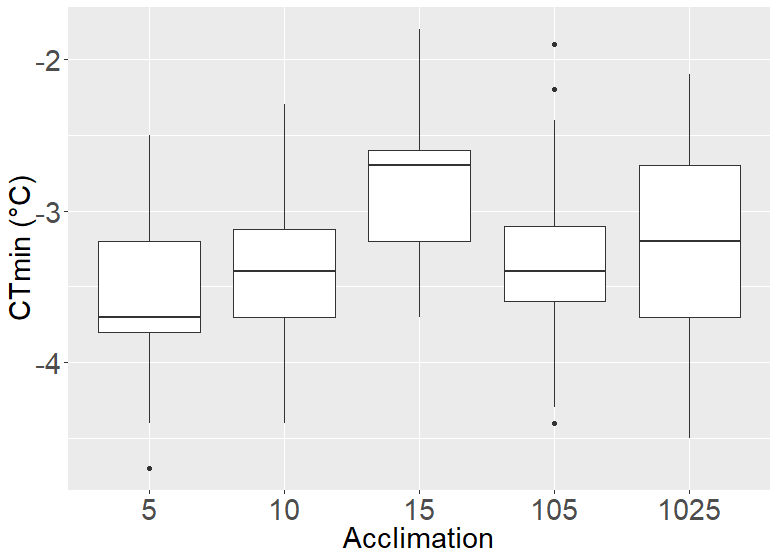

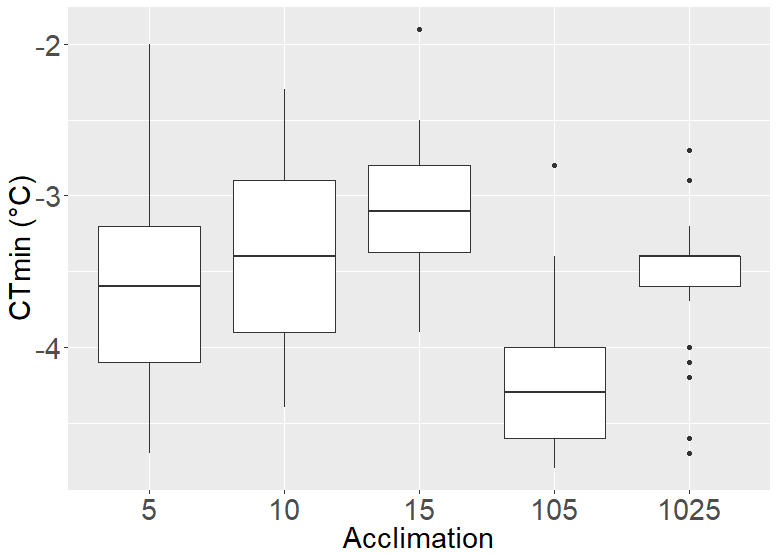


G. *Proisotoma* sp. (A) H. *Mucrosomia caeca* (I)


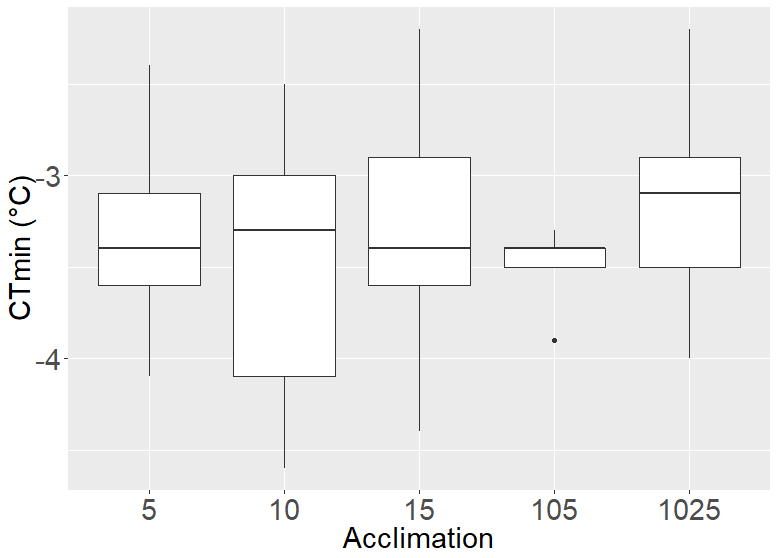


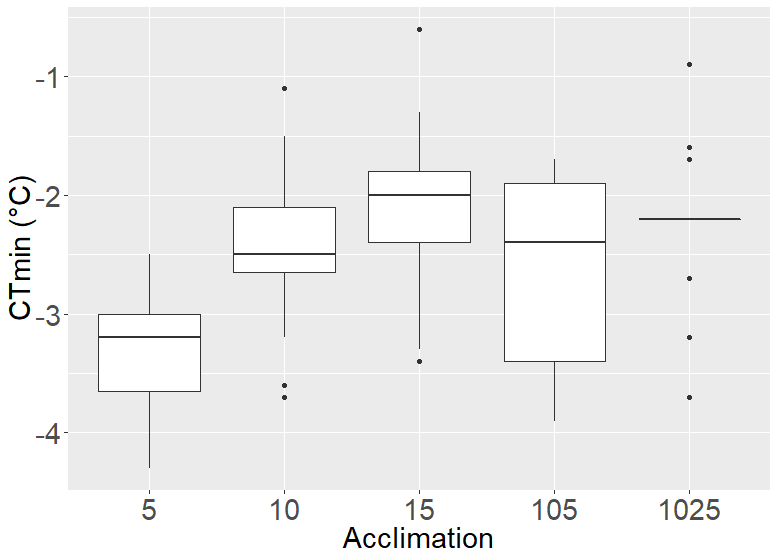


I. *Parisotoma insularis* (I) J. *Tullbergia bisetosa* (I)


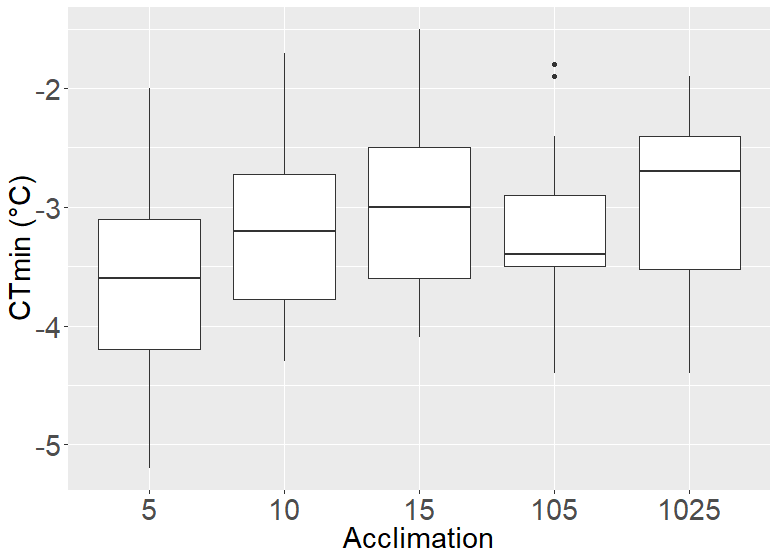

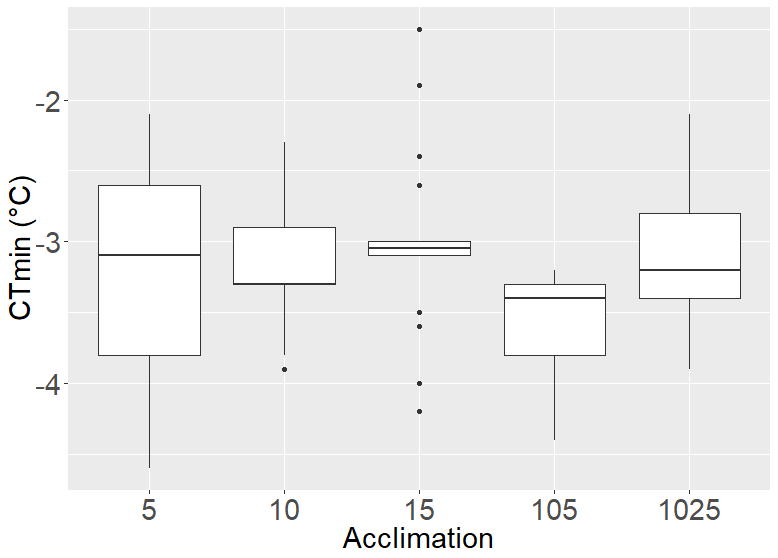

Supplement: suppl_data_coaa049 [file suppl_data_coaa049.zip › Phillips_et_al._Supplementary_Tables_and_Figures.docx]
